# β-hydroxybutyrate serves as a regulator in ketone body metabolism through lysine β-hydroxybutyrylation

**DOI:** 10.1016/j.jbc.2025.108475

**Published:** 2025-04-02

**Authors:** Jie Fang, Zhenghui Hu, Ting Luo, Shiyin Chen, Jie Li, Huaping Yang, Xia Sheng, Xinji Zhang, Ziyu Zhang, Caifeng Xie

**Affiliations:** 1 School of Basic Medical Sciences, Jiangxi Medical College, Nanchang University, Nanchang, Jiangxi, PR China; 2 Department of Pathology, Jiangxi Maternal & Child Health Hospital, Nanchang, Jiangxi, PR China; 3 Department of Endocrinology, The Third Affiliated Hospital of Nanchang University, Nanchang, Jiangxi, PR China; 4 Department of Urology, The Second Affiliated Hospital of Nanchang University, Nanchang, Jiangxi, PR China

**Keywords:** ketone body, β-HB, OXCT1, HMGCS2, β-hydroxybutyrylation

## Abstract

**β**-hydroxybutyrate (**β**-HB; 3-hydroxybutyric acid) may serve as a signaling metabolite in many physiological processes beyond a fuel source for tissues. However, whether and how it is involved in ketone body metabolism is still unknown. The present study aims to investigate the role of lysine **β**-hydroxybutyrylation (Kbhb) modification mediated by **β**-HB in regulating ketone body metabolic homeostasis both *in vivo* and *in vitro*. The starvation ketosis and type 1 diabetes mouse models were introduced to evaluate the influence of **β**-HB on Kbhb modification in mice. The Kbhb modifications of 3-oxoacid CoA-transferase 1 (OXCT1) and HMG-CoA synthase 2, two rate-limiting enzymes involved in ketogenesis and utilization, showed a positive correlation with the level of **β**-HB both *in vitro* and *in vivo*. The modification levels of the enzymes increased during fasting but decreased after refeeding. However, the Kbhb modification level in all detected tissues showed minor change since the blood ketone body increased nonsignificantly in the type 1 diabetes mouse model. The *in vitro* experiments further indicated that mutation at the Kbhb modification site significantly inhibited the enzymatic activity of OXCT1 but not HMG-CoA synthase 2. Sirtuin 1 (SIRT1) and CREB-binding protein (CBP) were identified both *in vitro* and *in vivo* as potential Kbhb dehydrogenase and transferase for OXCT1, respectively. Kbhb modification at lysine 421 of OXCT1 increases its enzyme activity during **β**-HB accumulation, accelerating the utilization of the ketone body and finally maintaining metabolism homeostasis. Our present study proposes a new ketone body metabolic regulatory mode primarily mediated by Kbhb modifications of OXCT1 during **β**-HB accumulation.

Ketone bodies, including β-hydroxybutyrate (β-HB, 3-hydroxybutyric acid, 70%), acetoacetate (AcAc, 30%), and acetone, are primarily used as an energy source by the liver and as an alternative to glucose for extrahepatic tissues (1). Under physiological conditions, the plasma concentration of ketone bodies in humans is usually around 0.05 to 0.1 mM, but in the conditions of enhanced ketone body production caused by prolonged exercise, starvation, carbohydrate restriction/ketogenic diet, or insulin deficiency, their level can reach 5 to 7 mM and even 20 mM, which indicates a concentration of diabetic ketoacidosis (DKA) (2). Although ketoacidosis is a pathological condition, mild ketosis induced by ketogenic diets, intermittent fasting, or caloric restriction has been demonstrated to be beneficial in animal models, with improved metabolism, extended lifespan, and improved neurological responses. In humans, the ketogenic diet may help alleviate neurological dysfunction (3). However, persistent mild ketogenic diet–induced elevated levels of low-density lipoprotein may increase the risk of cardiovascular disease (4, 5, 6). Ketone bodies can be used as an alternative energy and a metabolic signal molecule to affect the occurrence and development of many diseases, whereas the study on the regulatory mechanism underlying the ketone body metabolism has not been fully addressed.

HMG-CoA synthase 2 (HMGCS2) is a key enzyme in ketone body synthesis, located at the center of the ketone body synthesis regulatory network, and its activity is regulated by succinylation and acetylation after translation, by mitochondrial desuccinylation enzyme Sirt5 (7) and deacetylase Sirt3 (8), respectively. 3-Oxoacid CoA-transferase 1 (OXCT1, SCOT) is a key enzyme in extrahepatic ketone body utilization, which is widely distributed in the heart, brain, kidney, and other extrahepatic tissues (9). However, whether and how the HMGCS2 and OXCT1 are involved in the regulatory mechanism of ketone body metabolism is still largely unknown.

In recent studies, β-HB was found to be an important substrate in mediating a novel protein post-translational modification (lysine β-hydroxybutyrylation [Kbhb]) *via* its activated thioester form, β-hydroxybutyryl-CoA (10). Kbhb occurs not only on histones but also in cytoplasmic and mitochondrial proteins (11). Therefore, Kbhb may be involved in the regulation of mitochondrial metabolism. In addition, it has been reported that Kbhb occurs in the rate-limiting methionine cycle enzyme *S*-adenosyl-l-homocysteine hydrolase (AHCY), which affects the activity of AHCY and the methionine cycle (12). As important enzymes involved in ketone body metabolism, HMGCS2 and OXCT1 were reasonably proposed to be modified by Kbhb. Consistent with our hypothesis, a mass spectrometry database search suggested that HMGCS2 and OXCT1 may undergo β-HB-mediated Kbhb after translation (13, 14). However, the molecular mechanism underlying this novel modification of HMGCS2 and OXCT1 is still unknown as well as the role of Kbhb modification of HMGCS2 and OXCT1 in ketone body metabolism.

In the present study, the starvation ketosis mice model was established and used to evaluate the Kbhb modification levels of OXCT1 and HMGCS2 in different tissues and organs *in vivo*. The Kbhb modification sites were confirmed with mutants of OXCT1 and HMGCS2 in cells, and the enzyme activities were determined at various concentrations of β-HB. In addition, the modification processes mediated by lysine acetyltransferases (KATs) and histone deacetylases were further explored both *in vitro* and *in vivo*.

## Results

### OXCT1 and HMGCS2 undergo Kbhb modification in a β-HB concentration–dependent manner *in vitro*

**Figure 1:**
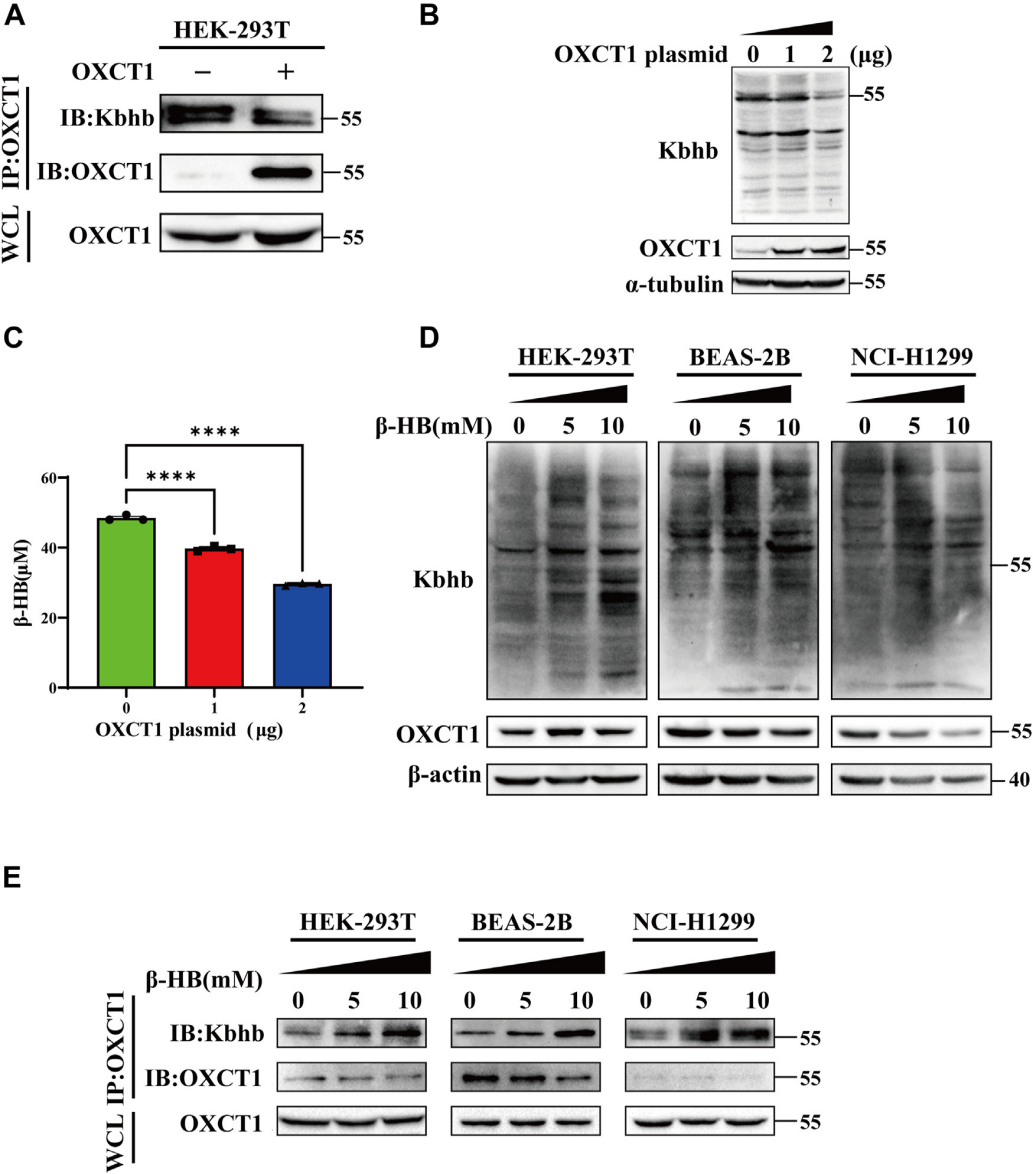
**OXCT1 undergoes Kbhb modification in a β-HB concentration–dependent manner in different types of cells.***A*, transfection of OXCT1 plasmids (1 μg) into HEK-293T cells, followed by detection of Kbhb modification levels through immunoprecipitation with antibodies against OXCT1, respectively. *B*, detection of global Kbhb modification level after transfecting different amounts of the OXCT1 plasmid (1 and 2 μg) into HEK-293T cells. *C*, measurement of β-HB content in HEK-293T cells transfected with different amounts of OXCT1 plasmid (0, 1, and 2 μg) (n = 3 biological replicates). *D*, detection of global Kbhb modification levels in HEK-293T cells, normal lung epithelial BEAS-2B cells, and non–small cell lung cancer NCI-H1299 cells treated with varying concentrations of β-HB (0, 5, and 10 mM). *E*, the OXCT1-specific Kbhb modification levels in HEK-293T cells, BEAS-2B cells, and NCI-H1299 cells were detected when treated with different concentrations of β-HB using coimmunoprecipitation assay. Data are expressed as mean ± SEM, and ∗∗∗∗*p* < 0.0001. β-HB, β-hydroxybutyrate; HEK-293T, human embryonic kidney-293T cell line; IB, immunoblotting; IP, immunoprecipitation; Kbhb, lysine β-hydroxybutyrylation; OXCT1, 3-oxoacid CoA-transferase 1; WCL, whole cell lysate.

For the whole ketone body metabolic pathway, HMGCS2 and OXCT1 serve as rate-limiting enzymes in ketone body production and utilization, respectively. Therefore, the expression and enzyme activity of HMGCS2 and OXCT1 may be key factors regulating ketone body metabolism. The mass spectrometry data of Luo *et al.* (13) and Huang *et al.* (14) indicated that HMGCS2 and OXCT1 would be extensively modified by Kbhb. In the present study, we further demonstrated that OXCT1 and HMGCS2 would undergo Kbhb modification in a β-HB concentration–dependent manner (Fig. 1).

The level of Kbhb modification in whole-cell lysis and OXCT1-specific Kbhb modification gradually decreased with the increasing OXCT1 expression (Fig. 1, *A* and *B*). This led us to suspect that the overexpression of OXCT1 reduces intracellular β-HB levels, thereby affecting overall Kbhb modification. To verify this hypothesis, changes in total β-HB were detected in human embryonic kidney-293T (HEK-293T) cells. A decrease in intracellular β-HB content was observed with increasing OXCT1 expression (Fig. 1*C*). To further support this idea, we treated cells with different concentrations of β-HB and examined changes in both global and OXCT1-specific Kbhb levels (*left panel*, Fig. 1, *D* and *E*). The results showed that both OXCT1-specific and global Kbhb modification levels increased with higher concentrations of β-HB treatment. In addition, β-HB would cause global Kbhb modification in different types of cells. The Kbhb levels in non–small cell lung cancer NCI-H1299 cells and normal lung epithelial cells (BEAS-2B) were also elevated with increasing concentrations of β-HB (*right panel*, Fig. 1, *D* and *E*). The results indicated that β-HB modulates global Kbhb levels in a concentration-dependent manner across various cell types.

**Figure 2:**
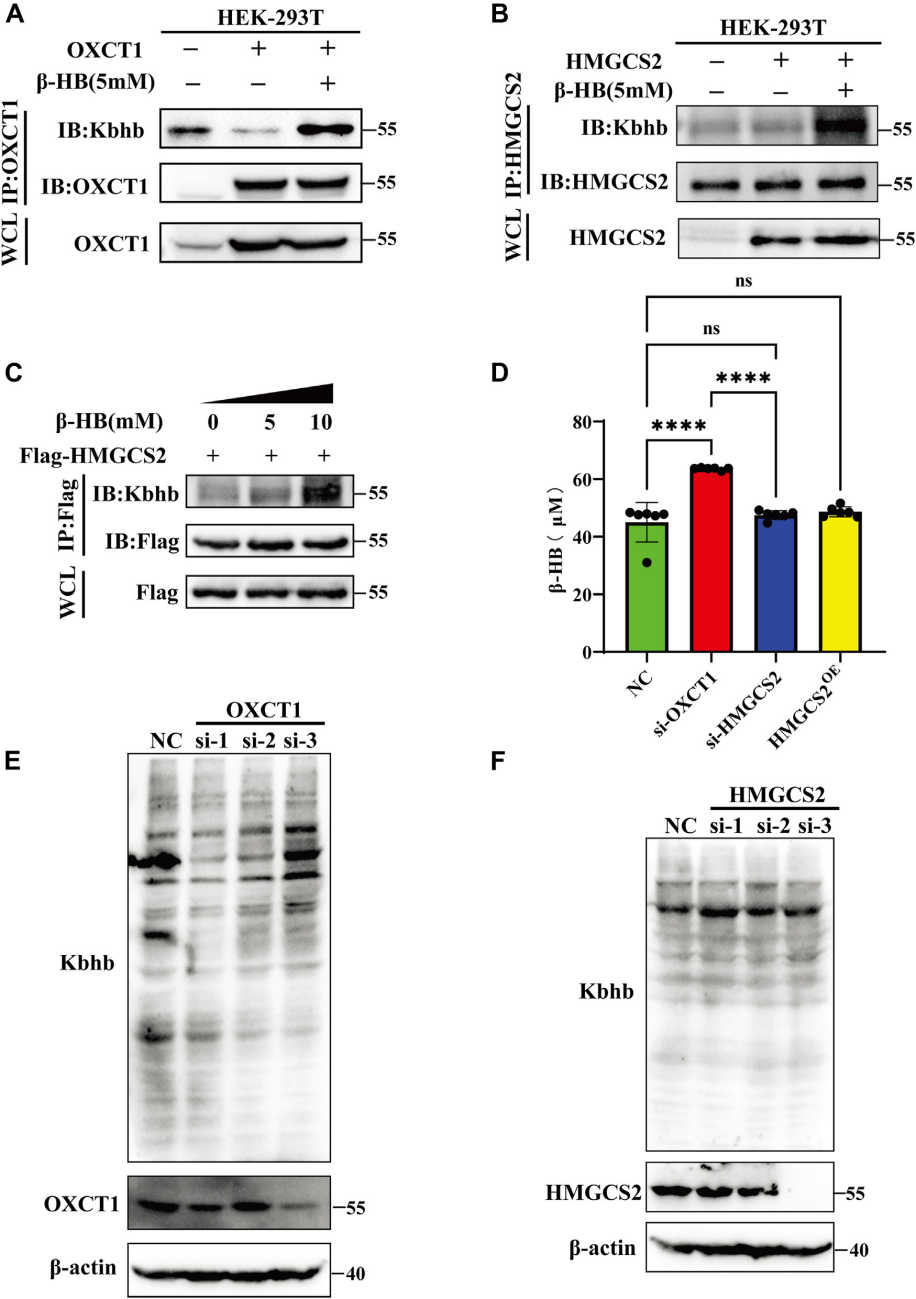
**OXCT1, not HMGCS2, was identified as the key regulator of the global Kbhb level by modulating the intracellular β-HB concentration**. *A*, transfection of the OXCT1 plasmid into HEK-293T cells treated with 5 mM β-HB for 24 h simultaneously, the Kbhb modifications in OXCT1 were detected. *B*, transfection of HMGCS2 plasmids (1 μg) into HEK-293T cells, followed by detection of Kbhb modification levels through immunoprecipitation with antibodies against HMGCS2, respectively. *C*, the Kbhb modification level changes in HMGCS2 when treated with different concentrations of β-HB were determined by coimmunoprecipitation assay. *D*, intracellular β-HB concentrations were detected after HEK-293T cells transfected with siRNA of human OXCT1 and HMGCS2 or HMGCS2 overexpression plasmid (HMGCS2^OE^). *E*, HEK-293T cells were transfected with negative control (NC) siRNA or siRNA (siRNA1, siRNA2, and siRNA3) of human OXCT1, and the global Kbhb level was detected by Western blot. *F*, HEK-293T cells were transfected with negative control (NC) siRNA or siRNA (siRNA1, siRNA2, and siRNA3) of human HMGCS2, and the global Kbhb level was detected by Western blot. Data are expressed as mean ± SEM. ∗∗∗∗*p* < 0.0001, ns, not significant. β-HB, β-hydroxybutyrate; HEK-293T, human embryonic kidney-293T cell line; HMGCS2, HMG-CoA synthase 2; Kbhb, lysine β-hydroxybutyrylation; OXCT1, 3-oxoacid CoA-transferase 1.

To further clarify the role of OXCT1 and HMGCS2 in Kbhb modification, exogenous gene expression and RNA interference experiments were conducted. When cells were supplemented with β-HB, the decrease in the Kbhb modification level caused by overexpression of OXCT1 was restored (Fig. 2*A*). However, HMGCS2's Kbhb modification was not affected by HMGCS2 overexpression; it only increased with the presence of exogenous β-HB (Fig. 2, *B* and *C*). In addition, RNA interference experiments further demonstrated that intracellular β-HB levels in OXCT1 knockdown cells were significantly increased, with a corresponding elevation in Kbhb modification (Fig. 2, *D* and *E*). In contrast, the knockdown and overexpression of HMGCS2 appeared to have no obvious effect on either intracellular β-HB or Kbhb modification levels (Fig. 2, *D* and *F*). These results strongly indicated that OXCT1 but not HMGCS2 was involved in the global Kbhb modification process by regulating the intracellular β-HB concentration.

### The Kbhb levels of HMGCS2 and OXCT1 in starvation ketosis mice model

**Figure 3:**
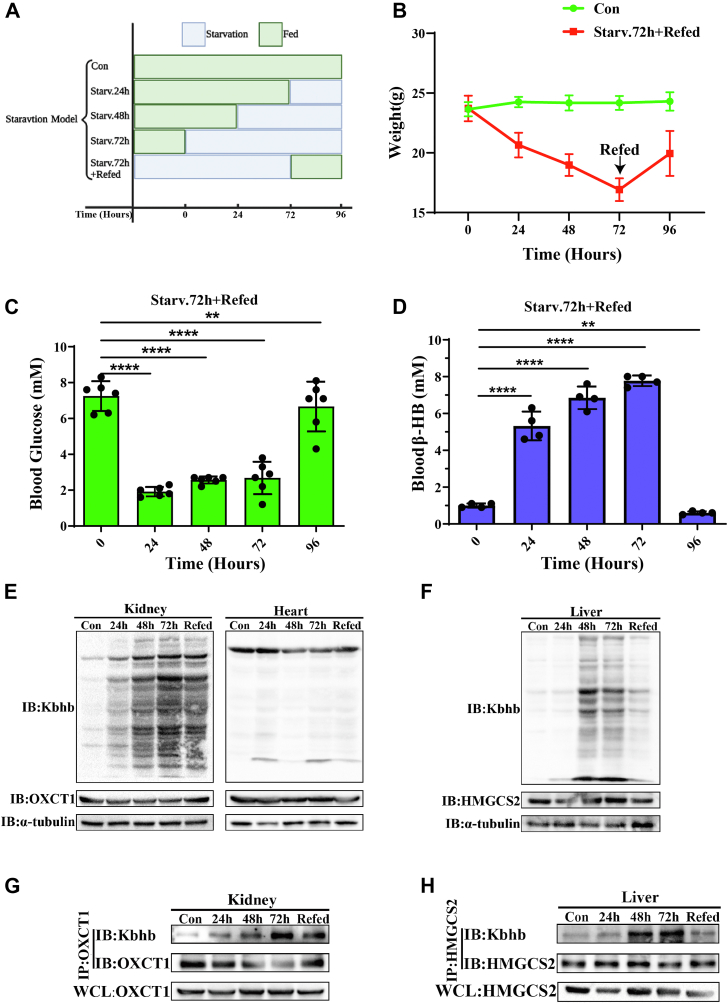
**The Kbhb levels of HMGCS2 and OXCT1 in starvation ketosis mice model.***A*, the schematic diagram of the starvation-induced ketosis mice model. *B*, bodyweight changes were monitored daily in the control mice and refed mice groups. *C* and *D*, blood glucose (n = 6) and blood β-HB (n = 4) levels were monitored daily in the refed mice group. *E*, expression levels of global Kbhb modifications in kidney and heart tissues of different experimental groups. *F*, levels of Kbhb modification in liver tissue of different experimental groups. *G* and *H*, Kbhb modification levels of OXCT1 in the kidney and HMGCS2 in the liver of mice were measured in different experimental groups. Data are expressed as mean ± SEM. ∗∗*p* < 0.01 and ∗∗∗∗*p* < 0.0001. β-HB, β-hydroxybutyrate; HMGCS2, HMG-CoA synthase 2; Kbhb, lysine β-hydroxybutyrylation; OXCT1, 3-oxoacid CoA-transferase 1.

To further explore the Kbhb modifications of OXCT1 and HMGCS2 *in vivo*, a starvation-induced ketosis mice model was established by inducing ketogenesis through fasting treatment. Mice were divided into five groups as described in Figure 3*A*: group 1 (control group, without fasting), group 2 (fasting for 24 h, 1 day), group 3 (fasting for 48 h, 2 days), group 4 (fasting for 72 h, 3 days), and group 5 (refeeding for 24 h after fasting for 72 h, 4 days). The body weight in the control and refed group mice was determined daily. The results showed that the weight of the control group mice increased slightly, whereas the weight of the refed group mice gradually decreased with increasing duration of fasting but recovered after resuming feeding (Fig. 3*B*). Blood glucose and ketone body (β-HB) levels in the refed groups were also detected daily (Fig. 3, *C* and *D*). A decrease in blood glucose was detected during fasting but recovered after refeeding. At the same time, blood ketone body levels increased during fasting but dramatically decreased after refeeding.

The Kbhb modification levels of OXCT1 and HMGCS2 in various organs were further determined. Four main ketone body utilization tissues (kidney, brain, heart, and skeletal muscle) and the main ketone body production tissue (liver) were collected, and the global and OXCT1-specific/HMGCS2-specific Kbhb levels were further detected (Fig. 3, *E*–*H*). The results revealed that the kidney would be the most sensitive tissue to Kbhb modification as well as the tissue with the highest concentration of β-HB among the four main ketone body utilization tissues (Figs. 3*E* and S1*A*). The Kbhb levels in mice kidney and liver tissues increased with the duration of fasting but decreased after refeeding, which showed similar characteristics with the changes of blood β-HB levels (Fig. 3, *E* and *F*). For the Kbhb modification of OXCT1 in the kidney and HMGCS2 in liver tissue, the modification levels also changed with the duration of fasting but decreased after refeeding (Fig. 3, *G* and *H*). In addition, the Kbhb levels of OXCT1 exhibited similar changes characteristic as those in other keto-utilizing organs (Fig. S1*B*).

### The Kbhb levels of HMGCS2 and OXCT1 in streptozotocin-induced type 1 diabetes mellitus mice model

**Figure 4:**
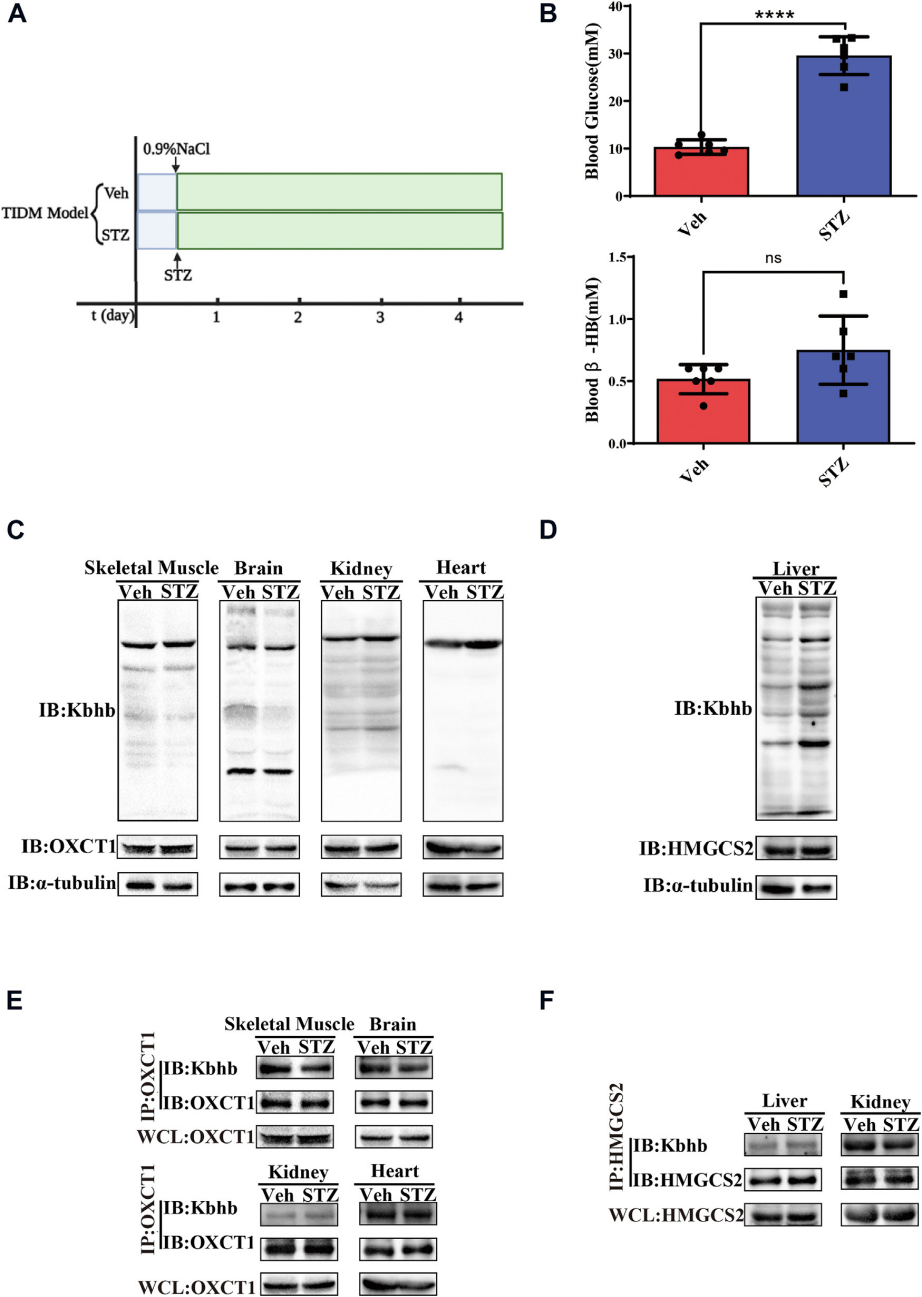
**The Kbhb levels of HMGCS2 and OXCT1 in streptozotocin (STZ)-induced T1DM mice model.***A*, the schematic diagram of the STZ-induced T1DM mice model. Vel, vehicle control. *B*, blood glucose (*upper panel*) and β-HB (*lower panel*) levels in mice after saline or STZ injection (n = 6). *C*, global Kbhb modification levels and OXCT1 expression in various organs (skeletal muscle, heart, kidney, and heart) of mice after saline or STZ injection. *D*, HMGCS2 expression in the liver of mice after saline or STZ injection. *E*, Kbhb modification levels of OXCT1 in various organs (skeletal muscle, heart, kidney, and heart) of mice after saline or STZ injection. The whole cell lysate (WCL) sample used is identical to that in *C*, and the coimmunoprecipitation assay was performed using this sample simultaneously. Therefore, the bands of OXCT1 in *C* were reused in this panel. *F*, Kbhb modification levels of HMGCS2 in mouse liver and kidney after saline or STZ injection. The WCL sample used is identical to that in *D*, and the coimmunoprecipitation assay was performed using this sample simultaneously. Therefore, the bands of HMGCS2 in *D* were reused in this panel. Data are expressed as mean ± SEM. ∗∗∗∗*p* < 0.0001, ns, not significant. β-HB, β-hydroxybutyrate; HMGCS2, HMG-CoA synthase 2; Kbhb, lysine β-hydroxybutyrylation; OXCT1, 3-oxoacid CoA-transferase 1; T1DM, type 1 diabetes mellitus.

In the mouse starvation model mentioned previously, we observed an increase in blood ketone levels accompanied by a decrease in blood glucose. To explore the influence of blood glucose on Kbhb modification, we induced a type 1 diabetes model in mice using streptozotocin (STZ) and determined the changes in Kbhb levels in various tissues. After fasting for 12 h, mice were intraperitoneally injected with STZ or normal saline (control group) (Fig. 4*A*). Blood glucose was measured after 4 days of treatment, and mice with blood glucose levels higher than 16.8 mM were considered successful. Compared with the control group, mice treated with STZ exhibited significantly higher blood glucose levels, whereas the blood ketone body (β-HB) level of mice found no significant change (Fig. 4*B*). Subsequent analysis of Kbhb levels in different organs revealed that consistent with the unchanged blood ketone body level, there was no significant alteration in Kbhb modification across ketone body utilization tissues between control mice and STZ-treated mice (Fig. 4*C*), and the Kbhb levels seem slightly increased in liver tissue (Fig. 4*D*). In addition, the Kbhb levels of OXCT1 and HMGCS2 showed no significant changes compared with the control group (Fig. 4, *E* and *F*). Therefore, these results further indicate that the Kbhb modification level is mainly regulated by blood ketone body concentration but not glucose.

### The level of Kbhb modulated the enzyme activities of OXCT1

**Figure 5:**
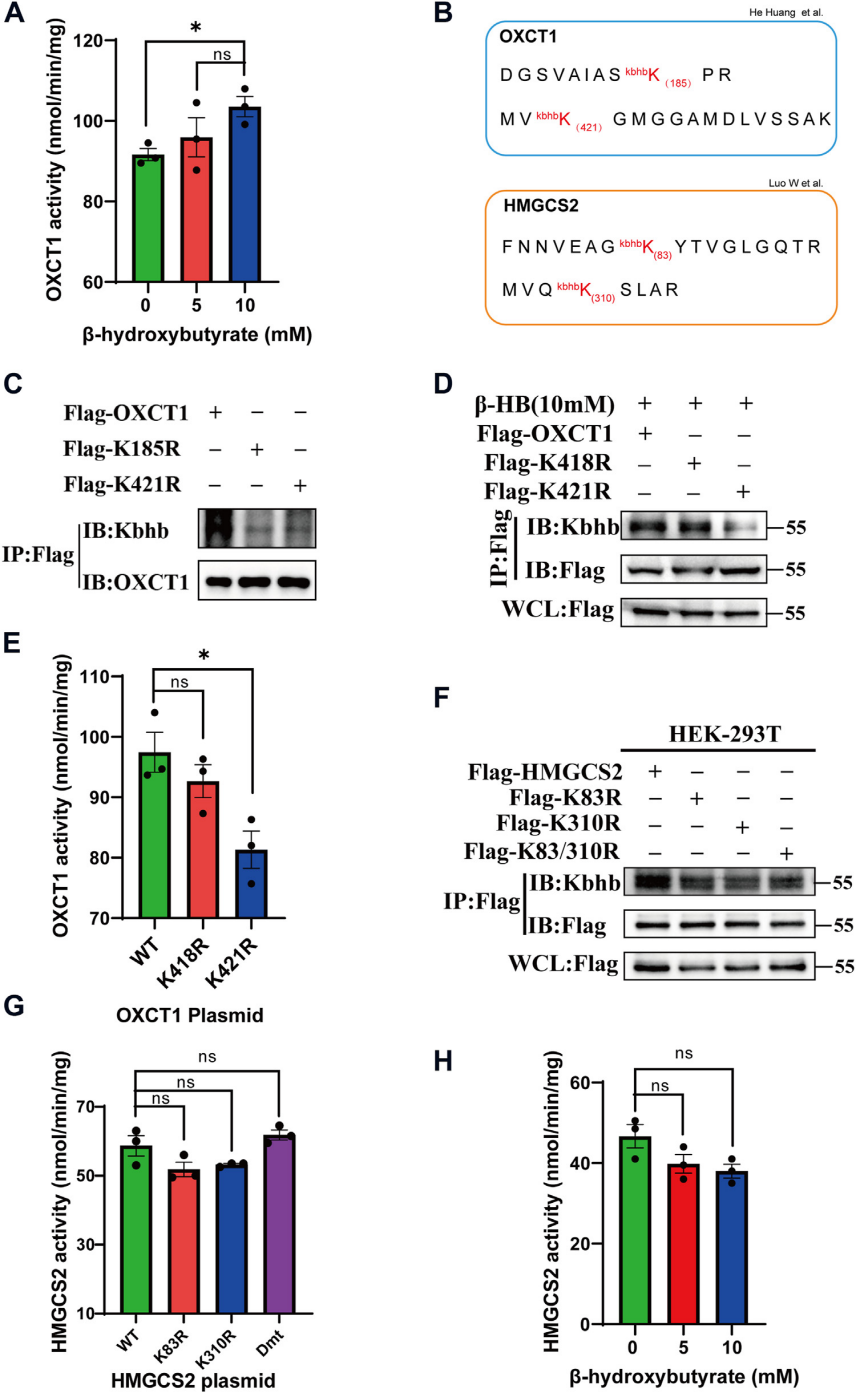
**The level of Kbhb modulated the enzyme activities of OXCT1.***A*, enzymatic activity of OXCT1 in HEK-293T cells treated with different concentrations of β-HB (5 and 10 mM). *B*, mass spectrometry detection of Kbhb-modified peptides of HMGCS2 and OXCT1. *C*, FLAG-tagged WT, K185R, and K421R mutant OXCT1 were transfected into HEK-293T to detect changes in Kbhb modification. *D* and *E*, FLAG-tagged WT, K418R, and K421R mutant OXCT1 were transfected into HEK-293T to detect changes in Kbhb modification (with 10 mM β-HB) and enzymatic activity. *F* and *G*, FLAG-tagged WT, K83R, K310R, and K83/310R mutant HMGCS2 (Dmt, double-site mutant) were transfected into HEK-293T to detect changes in Kbhb modifications and enzyme activities. *H*, enzymatic activity of HMGCS2 in HEK-293T cells treated with different concentrations of β-HB (0, 5, and 10 mM). Data are expressed as mean ± SEM. ∗*p* < 0.05, ns, not significant, n = 3. β-HB, β-hydroxybutyrate; HEK-293T, human embryonic kidney-293T cell line; HMGCS2, HMG-CoA synthase 2; Kbhb, lysine β-hydroxybutyrylation; OXCT1, 3-oxoacid CoA-transferase 1.

To explore the impact of Kbhb on the regulation of OXCT1 and HMGCS2, we initially investigated the enzymatic activity of OXCT1 by treating HEK-293T cells with varying concentrations of β-HB (Fig. 5*A*). The original data were collected every 2 min and lasted for 50 min to show the whole enzyme activity curve of OXCT1 (Fig. S2*A*). The results showed that the enzyme activity of OXCT1 was elevated with the increasing concentrations of β-HB, indicating that the Kbhb modification of OXCT1 would promote its catalytic activity.

As shown in Figure 5*B*, mass spectrometry data indicated that OXCT1 (K185 and K421) and HMGCS2 (K83 and K310) would be modified by Kbhb (13, 14). To further elucidate the specific Kbhb modification sites of OXCT1, the amino acids K185 and K421 were further demonstrated as Kbhb modification sites through immunoprecipitation (Fig. 5*C*). The OXCT1^K185R^ and OXCT1^K421R^ mutants were constructed, and the results have shown that the mutation at K185 and K421 decreased the Kbhb modification of OXCT1. As E344 and G424 are known to be active binding sites for succinyl-CoA (15), the amino acid K421, which is closer to the active binding sites, is suspected to be a potential site that would affect the enzymatic activity of OXCT1. Therefore, the role of the K421 site in OXCT1 was further investigated by the OXCT1^K421R^ mutant. As shown in Figure 5*D*, the addition of β-HB could not restore the decrease of the Kbhb level of OXCT1 with K421 site mutation. The K418R mutation was set as a negative control to avoid possible side effects of amino acid mutation. The results revealed no discernible impact on the level of Kbhb modification after introducing the K418 mutation (Fig. 5*D*). Subsequent examination of enzyme activity before and after the OXCT1 mutation showed a significant decrease in enzyme activity following the K421 mutation, whereas no significant change was observed after the K418 mutation (Fig. 5*E*). Meanwhile, the whole enzyme activity curve is shown in Fig. S2*B*. These results indicate that K421 would be an important Kbhb modification site of OXCT1 protein, and the Kbhb modification at this amino acid residue would elevate the enzymatic activity of OXCT1.

Similarly, the influence of Kbhb modification on the ketogenesis pathway was further explored through the detection of HMGCS2's Kbhb modification site (Fig. 5*F*). Mutation at this site resulted in a slight decrease in HMGCS2's Kbhb modification level when targeting residues K83 and K310. Subsequent assessment of enzyme activity before and after mutation did not show any significant changes (Fig. 5*G*). In addition, the enzymatic activity of HMGCS2 exhibited no significant change under different β-HB concentrations (Fig. 5*H*), indicating that Kbhb modification may not be a major regulatory factor of the enzymatic activity of HMGCS2. The original data were collected every 2 min and lasted for 80 min to show the whole enzyme activity curve of HMGCS2 (Fig. S2, *C* and *D*).

### The Kbhb-transferase and Kbhb-removing enzyme of HMGCS2 and OXCT1

**Figure 6:**
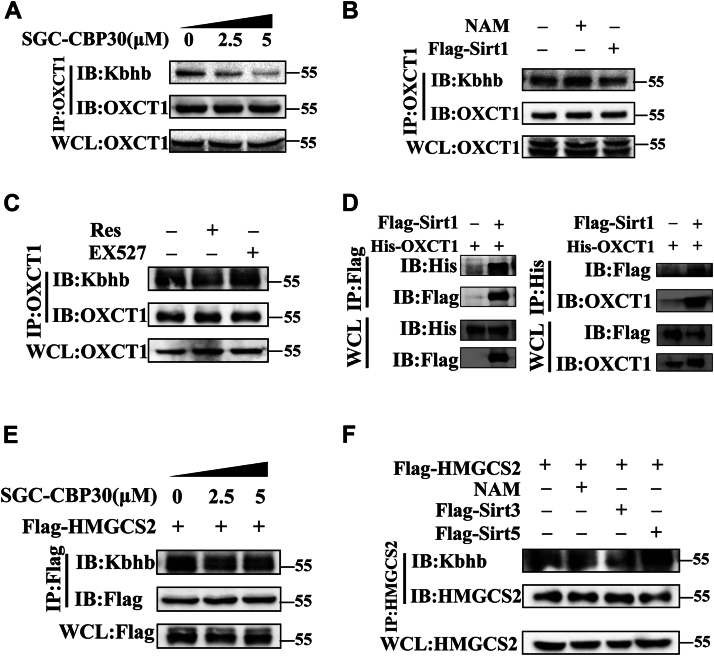
**The Kbhb-transferase and Kbhb-removing enzyme of HMGCS2 and OXCT1 *in vitro*.***A*, Kbhb modification levels in HEK-293T cells treated with varying concentrations of SGC-CBP30 (2.5 and 5 μM). *B*, Kbhb modification in HEK-293T cells transfected with FLAG-tagged SIRT1 plasmid or treated with the SIRT family inhibitor nicotinamide (NAM; 10 mM). *C*, Kbhb modification of OXCT1 in HEK-293T cells treated with the SIRT1 activator (Res, 10 μM) and inhibitor (EX527, 1 μM). *D*, protein interaction between OXCT1 and SIRT1 was detected by coimmunoprecipitation in HEK-293T cells. *E*, Kbhb modification levels of HMGCS2 in HEK-293T cells treated with different concentrations of SGC-CBP30 (2.5 and 5 μM). *F*, detection of Kbhb modification in HEK-293T transfected with FLAG-tagged SIRT3 and SIRT5 plasmids or treated with SIRTs' inhibitor (NAM). HEK-293T, human embryonic kidney-293T cell line; HMGCS2, HMG-CoA synthase 2; Kbhb, lysine β-hydroxybutyrylation; OXCT1, 3-oxoacid CoA-transferase 1; Res, resveratrol.

It has been reported that the CREB-binding protein (CBP) is one of the key protein KATs that catalyzed the process of Kbhb modification (14). To further elucidate the regulatory mechanism of Kbhb modification of OXCT1, HEK-293T cells were treated with varying concentrations of the CBP selective inhibitor (SGC-CBP30) (Fig. 6*A*). The results indicated that the Kbhb modification level of OXCT1 decreased in an SGC-CBP30 concentration–dependent manner, suggesting that CBP may mediate the Kbhb modification of OXCT1.

For the de-Kbhb process, sirtuin 1 (SIRT1) has been reported to catalyze de-Kbhb modification (14). The present results showed that Kbhb modification of OXCT1 was enhanced when HEK-293T cells were treated with nicotinamide, an inhibitor of the SIRT family. Meanwhile, the overexpression of SIRT1 decreased the Kbhb modification of OXCT1 (Fig. 6*B*). These results suggest that SIRT1 may function as a Kbhb-removing enzyme for OXCT1.

To further clarify the effect of SIRT1 on OXCT1 Kbhb modification, HEK-293T cells were treated with the SIRT1 activator resveratrol (Res) and the inhibitor Selisista (EX527) (Figs. 6*C* and S3*A*). The results showed that Res inhibited Kbhb modification of OXCT1, whereas EX527 enhanced its modification. Furthermore, a protein interaction between OXCT1 and SIRT1 was confirmed through coimmunoprecipitation experiments (Fig. 6*D*), indicating that SIRT1 is a Kbhb-removing enzyme of OXCT1.

As shown in Figure 6*E*, CBP also mediated the process of Kbhb modification of HMGCS2. Meanwhile, previous reports indicated the potential interactions between HMGCS2 and SIRTs, which led to the acetylation and succinylation of HMGCS2 (7, 8). The present overexpression assay revealed that both SIRT3 and SIRT5 have an impact on Kbhb modifications in HMGCS2 (Figs. 6*F* and S3*B*). Specifically, overexpression of SIRT3 led to inhibition of HMGCS2's Kbhb modification levels, whereas SIRT5 increased that modification, suggesting that SIRT3 may serve as a Kbhb-removing enzyme for HMGCS2.

**Figure 7:**
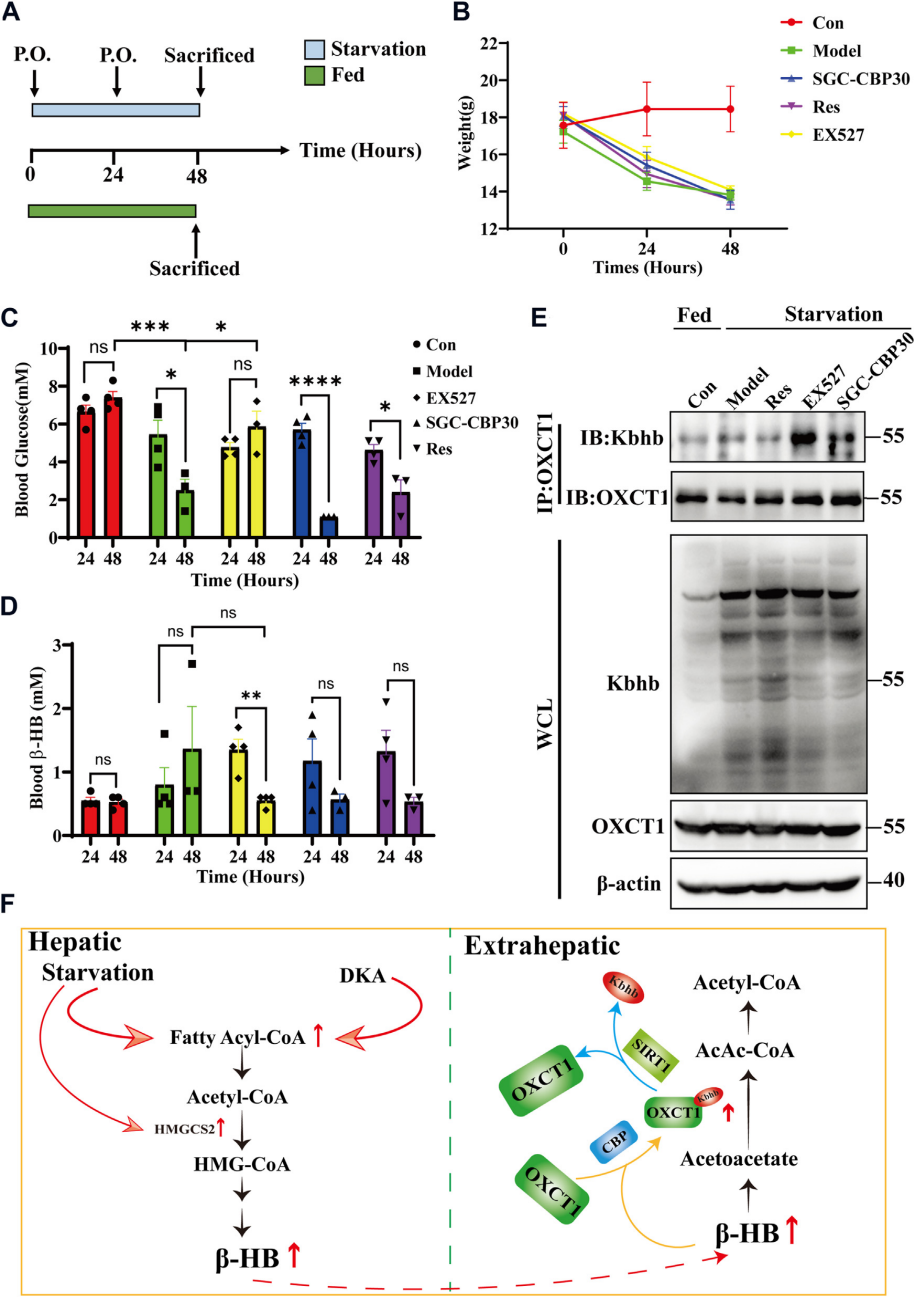
**The Kbhb-transferase and Kbhb-removing enzyme of HMGCS2 and OXCT1 *in vivo*.***A*, the schematic diagram of the inhibitor treatment mice model. P.O.: oral gavage. *B*, bodyweight changes of mice were monitored daily in all groups. *C* and *D*, blood glucose and β-HB were detected at 24 and 48 h in all groups (n = 4 at 24 h, n = 3 at 48 h). *E*, global and OXCT1-specific Kbhb modification levels were detected by coimmunoprecipitation assay in all groups (n = 3). *F*, the schematic diagram of Kbhb modification function in keeping ketone body metabolic homeostasis under physiological or pathological conditions. Data are expressed as mean ± SEM. ∗*p* < 0.05, ∗∗*p* < 0.01, ∗∗∗*p* < 0.001, and ∗∗∗∗*p* < 0.0001. β-HB, β-hydroxybutyrate; HMGCS2, HMG-CoA synthase 2; Kbhb, lysine β-hydroxybutyrylation; ns, not significant; OXCT1, 3-oxoacid CoA-transferase 1.

The inhibitor treatment experiments were conducted based on the starvation-induced mice ketosis model to further clarify the “writer” and “eraser” of Kbhb of OXCT1 *in vivo*. Mice were fasted and treated with KAT CBP selective inhibitor SGC-CBP30 (P.O., 20 mg/kg, daily), SIRT1 inhibitor Ex527 (P.O., 20 mg/kg, daily), SIRT1 activator Res (P.O., 50 mg/kg, daily), and solvent (P.O., model group, daily) for 48 h, respectively. Mice in the control group were fed for 48 h. The schematic diagram of the experiment process is shown in Figure 7*A*. The body weight was recorded during the experiment and shown in Figure 7*B*. After fasting for 24 and 48 h, the blood glucose was decreased in model group mice as well as that in SGC-CBP30 and Res (Fig. 7*C*). Meanwhile, blood β-HB levels were increased in model mice but decreased in SGC-CBP30, Ex527, and Res-treated mice (Fig. 7*D*). Kidney tissues were collected, and the global and OXCT1-specific Kbhb levels were determined by Western blotting and coimmunoprecipitation assay (Figs. 7*E* and S3, *C* and *D*). Global Kbhb modification was increased after fasting in the model group, whereas that was decreased in SGC-CBP30 and Ex527-treated mice (*upper panel* of Figs. 7*E* and S3*C*). Notably, the OXCT-specific Kbhb level was significantly elevated in Ex527-treated mice but decreased in Res-treated mice (*lower panel* of Figs. 7*E* and S3*D*). Since CBP and SIRTs serve as “writer” and “eraser,” respectively, for many post-translational modification processes and proteins, it seems not surprising that the changes are different between global and OXCT-specific Kbhb levels. These results indicated that CBP facilitates Kbhb modifications in both OXCT1 and HMGCS2, and SIRT1 and SIRT3 may serve as transferases for OXCT1 and HMGCS2, respectively. The main conclusion of the present study is summarized in Figure 7*F*.

## Discussion

Kbhb is a novel type of protein post-translational modification mediated by β-HB, which occurs not only on histones but also widely in cytoplasmic and mitochondrial proteins. As a result, Kbhb impacts metabolism in the nucleus and participates in cytoplasmic and mitochondrial metabolism, including fatty acid metabolism (16), tricarboxylic acid cycle, ketone body, and one-carbon metabolism (12, 17). Currently, research on Kbhb modification primarily focuses on histone proteins, with an incomplete understanding of Kbhb modification on non-histone proteins. In the present study, we aim to figure out the underlying mechanism of β-HB-mediated Kbhb of OXCT1 in regulating ketone body metabolism. HMGCS2 and OXCT1 are pivotal enzymes in ketone body synthesis and catabolism regulation, and our results demonstrated that β-HB mediates the Kbhb of HMGCS2 and OXCT1 at both cellular and systemic levels. The sites of Kbhb modification on HMGCS2 and OXCT1, and their respective transferase and removing enzymes for Kbhb modifications, were further identified. In addition, the effect of Kbhb levels on the enzyme activities of HMGCS2 and OXCT1 protein was explored. Our work may shed light on the potential function of Kbhb modification in maintaining ketone body metabolic homeostasis.

Metabolite often represents the initial alteration in various physiological or pathological conditions, such as hunger, movement, ketogenic diet, and diabetes (18, 19). Under these conditions, the body tends to alter its metabolic profile to enhance the adaptation to changes. The regulation of metabolic pathways is primarily governed by the expression and activity of key enzymes, with gene expression regulation often exhibiting a sluggish and delayed response. In this case, rapid regulation of enzyme activity may be crucial for maintaining metabolic homeostasis (20). It has been reported that β-HB can act as a signaling mediator to play a role in physiological activities, such as oxidative stress gene expression (21), sympathetic nerve activity (22, 23), and release of nonesterified fatty acids (24, 25, 26, 27). In ketone body metabolism, β-HB has been found to inhibit ketone body production by suppressing fatty acid release from adipocytes (1). Meanwhile, our present study further revealed that an increase in the level of Kbhb modification enhances OXCT1 enzyme activity while having no significant effect on HMGCS2 activity. Furthermore, we observed an increase of HMGCS2′s expression in the liver tissue during fasting, whereas there was no significant change in OXCT1′s expression. Our study suggests that β-HB utilizes a bidirectional regulation mechanism to maintain its metabolic homeostasis when cells or mice are under enhanced lipolysis and ketogenesis conditions. β-HB inhibits the production of the ketone body in liver tissue by suppressing fatty acid release and promotes the utilization of the ketone body in extrahepatic tissues by increasing the OXCT1 enzyme activity mediated with Kbhb modification. These processes finally help to maintain ketone body metabolic homeostasis in the body.

Generally, it is an important regulatory mode for cell metabolism through protein post-translational modifications mediated by metabolites, such as acetylation, glycosylation, and succinylation (28, 29, 30). Previous studies have shown that succinylation inhibits HMGCS2 enzyme activity (7). It suggests that activation of the ketogenic pathway during fasting may be related to decreased succinylation levels alongside increased expression levels for HMGCS2. Meanwhile, our present study showed that activation of the ketone body utilization pathway is mainly mediated by increased levels of Kbhb modification for OXTC1. Therefore, the crosstalk between succinylation and Kbhb needs further exploration in ketone body metabolism.

As a key enzyme in ketone body metabolism, OXCT1 is primarily responsible for facilitating the transfer of CoA from succinyl-CoA to AcAc to generate acetoacetyl-CoA (9, 31). A molecular docking study indicated that the amino acid G424 of OXCT1 would serve as a potential binding site to its substrate (succinyl-CoA) (18). Since K421 is near the potential binding site G424, it is proposed that Kbhb modification at residue K421 of OXCT1 could play a more pivotal role in regulating its enzymatic activity in our present study. As expected, we found a significant inhibition, which was positively associated with the K421R mutant but not the K418R mutant. The findings suggest that amino acid residue K421 of OXCT1 functions as a crucial regulatory locus governing its enzyme activity and Kbhb modification. Furthermore, a very recent study on the OXCT1 succinylation modification also emphasized the importance of the K421 site for OXCT1 function (32).

In the STZ-induced type 1 diabetes mellitus (T1DM) mice model, a rapid increase in blood glucose levels was observed. However, the blood ketone body levels showed no significant alteration. These findings imply that neither the lipolysis nor the ketone body synthesis pathway was prominently activated in the STZ-induced mice. Notably, the raw data of our ongoing study on rat DKA model indicated that the Kbhb modification level of OXCT1 was increased obviously with the blood β-HB concentration in DKA rats (data not shown). The difference between the STZ-induced T1DM and DKA model is the usage of a high-fat diet. In the DKA model, rats were injected with STZ and followed by feeding a high-fat diet. Hence, it can be inferred that blood glucose is not the sole trigger for lipolysis and ketogenesis. Previous reports have indicated that lipid production requires glucose and glutamine synergistic action (33). We suspected the unaffected ketone body level may be due to the out of sync between blood glucose and lipolysis, but the exact mechanism underlying these needs further investigation.

Our results indicated that CBP would be the potential KAT for Kbhb modification of OXCT1. However, whether CBP is the only or critical KAT for OXCT1's Kbhb process remains unknown. A recent study reported that OXCT1 can be succinylated after incubation of succinyl-CoA, suggesting that OXCT1 would undergo succinylation without KAT (32). Whereas, whether OXCT1 can be hydroxybutyrylated without specific KATs also needs to be further explored. Besides, in the inhibitor treatment experiment, mice suffered more injury with the oral gavage of the CBP and SIRT1 inhibitor, even the solvent. Therefore, the mice were treated for only 48 h, not 72 h. Blood β-HB levels were decreased at 48 h in all compound-treated mice compared with those at 24 h; we suspected that would be the result of a dramatic decrease of lipid oxidation.

In conclusion, our study highlights a novel role of β-HB in maintaining ketone body metabolism. β-HB accumulation under physiological and pathological conditions may serve as a sensor and regulator to modulate the activity of enzymes involved in ketone body metabolism. When a rapid escalation in circulating β-HB levels is caused, this metabolite can enhance the catabolic activity of OXCT1 *via* Kbhb modification at amino acid K421, thereby maintaining the homeostatic balance within the overall ketone body metabolism.

## Experimental procedures

### Antibodies

The rabbit polyclonal antibodies α-tubulin (11224-1-AP, 1:2000 dilution), the mouse monoclonal antibodies against OXCT1 (67836-1-Ig, 1:5000 dilution), DYKDDDDK tag (FLAG) (66008-1-Ig, 1:5000 dilution), and β-actin (66009-1-Ig, 1:5000 dilution) were purchased from Proteintech Group, Inc. The anti-β-hydroxybutyryllysine rabbit polyclonal antibody (Kbhb) (PTM-1201) was ordered from PTM Biolab. The mouse monoclonal antibody against the mHMCGS (HMGCS2) monoclonal antibody (sc-393256, 1:1000 dilution) was obtained from Santa Cruz Biotechnology, Inc.

### Reagents

AcAc-CoA (A1625), Ac-CoA (A2181), dithionitrobenzoic acid (8130), succinyl-CoA (S1129), iodoacetamide (A3221), AcAc (A8509), and nicotinamide (47865-U) were purchased from Sigma. β-HB (HY-113378), PEG300, and Tween-80 were ordered from MedChemExpress. SGC-CBP30 (M9141), Selisistat (Ex527) (M1708), and Res (M2267) were obtained from AbMole BioScience.

### Plasmids

PCR-amplified OXCT1, SIRT1, SIRT3, and SIRT5 were cloned into the FLAG-pCDNA3.1 plasmid vector. OXCT1 (K418R) and OXCT1 (K421R) mutation plasmids were generated using the Fast MultiSite Mutagenesis System (FM201-01). HMGCS2 (wildtype), HMGCS2 (K83R), HMGCS2 (K310R), and HMGCS2 double-site (K83/310R) mutation plasmids were purchased from the Public Protein/Plasmid Library.

### Cell culture and transfection

The human lung epithelial cell line BEAS-2B, LUAD cell lines (NCI-H1299), and human HEK-293T cell line were purchased from the National Collection of Authenticated Cell Cultures. All cell lines were validated by using short tandem repeat profiling and were free from mycoplasma contamination. Cells were cultured in a Dulbecco's modified Eagle's medium (Gibco, C11995500BT) or RPMI1640 medium (Gibco, C22400500BT) supplemented with 10% fetal bovine serum (SORFA). All cells were cultured under an atmosphere of 5% CO_2_ at 37 °C. For gene overexpression assay, cells at approximately 90% confluence were transfected with the indicated plasmids using a transfection reagent kit (OriGene; TT210003). For interfering RNAs, the targeted human OXCT1 and HMGCS2 siRNA (hOXCT1 and hHMGCS2) and negative control siRNA were synthesized by Guangzhou RiboBio Co, Ltd. The details of siRNA sequences are listed in Table S1. Cells at 60 to 80% confluence were transfected with indicated siRNA by using the Transfection System (RiboBio) according to the manufacturer's protocol.

### Animals

All animal experiments conformed to the Guide for the Care and Use of Laboratory Animals and were approved by the Institutional Committee of Laboratory Animal Experimentation of Nanchang University (approval no.: NCULAE-20220624016). Male BALB/c mice (6–8 weeks old, obtained from Hunan SLAC Laboratory Animal Co, Ltd; certificate number: SYXK2019-0004) were group housed under a 12 h light, 12 h dark cycle at room temperature and fed ab libitum with normal chow.

For starvation-induced mice ketosis experiments, 30 mice were divided into five groups randomly. Among them, four groups of mice were fasted for 0, 24, 48, and 72 h, and another group refed for 24 h after fasting for 72 h (total 96 h). Glucose and β-HB of mice were monitored daily. Tail blood samples were taken from mice and immediately dropped onto test strips for detection using the Blood Glucose and Ketone Monitoring System (FreeStyle Optium Neo; Abbott). At the end of the experiment, blood measurements and tissue were harvested.

For T1DM experiments, 12 mice were divided into two groups randomly. The model group mice were treated with 200 mg/kg STZ (Sigma; 572201) by i.p., whereas the control group mice received the same volume of saline instead. Blood measurements and tissue were collected at 4 days after injections when the induction of T1DM was evident.

For the inhibitor treatment experiments, 20 mice were divided into five groups randomly (control, model [solvent], SGC-CBP30 [20 mg/kg], Ex527 [20 mg/kg], and Res [50 mg/kg]). The control group mice had food and water available ad libitum. Mice in the other four groups were fasted and received daily oral gavage of SGC-CBP30 (20 mg/kg) (34), Ex527 (20 mg/kg) (35), Res (50 mg/kg) (36), or the solvent (10% dimethyl sulfoxide + 40% PEG300 + 5% Tween-80 + 45% saline) for 48 h. Blood glucose and β-HB of mice were monitored daily as described previously. At the end of the experiment, blood measurements and tissue were harvested.

### Coimmunoprecipitation assay

Cells were washed with PBS three times in 5 min and lysed with NP-40 buffer (150 mM NaCl, 20 mM β-glycerol phosphate, 1 mM sodium orthovanadate, 20 mM NaF, 0.5% Nonidet P-40, 20 mM Hepes, pH 7.4) with PMSF for 30 min at 4 °C. Then, the cell lysates were collected, and Protein G-Agarose (Roche; catalog no.: 11243233001) was added along with the indicated antibodies, with overnight incubation at 4 °C. The samples were washed with PBS three times and centrifuged at 3000*g* for 3 min at 4 °C between each wash. The samples were added to 2× loading buffer and boiled for 10 min.

### Western blotting

The samples were added to 10% SDS-PAGE and transferred to polyvinylidene difluoride membranes (Millipore; IPVH00010) and then blocked with 5% skim milk (Solarbio; D8340) for 1 h at room temperature. After incubation with the indicated primary antibodies, the membranes were washed with Tris-buffered saline with Tween-20 three times in 10 min and incubated with secondary antibodies for 1 h at room temperature. The membranes were washed three times with Tris-buffered saline with Tween-20 and stained with an ECL detection reagent (TIANGEN; PA112-01). Digital gel image analysis (TANON5500) was used to visualize the proteins.

For the tissue samples isolated from mice, an equal weight of tissue (50 mg) was obtained from three mice in the same group, and a total amount of 150 mg tissue was used for protein extraction and Western blotting. All the Western blot results for the animal experiments were obtained using tissue protein isolated from three mice in the inhibitor treatment experiment.

### Measurement of OXCT1 activity

Enzyme activities were measured as previously described with slight modifications (37, 38). Briefly, supernatant fractions of whole-cell lysis were collected, and OXCT1 enzymatic activity was measured by monitoring the conversion of AcAc and succinyl-CoA to AcAc-CoA. Assay mixtures (100 μl) contained 50 mM Tris–HCl (pH 8.5), 0.5 mM succinyl-CoA, 4 mM iodoacetamide, 10 mM MgCl_2_, and 10 mM AcAc. The reaction was started with the addition of AcAc at 30 °C. Microplate assays were performed using a SpectraMax 96, and the rate of acetoacetyl-CoA formation was monitored by the absorbance at 313 nm every 2 min. The original data were collected every 2 min and lasted for 50 min. The absorbance value at the time point of 10 min was used for enzymatic activity determination. Whole-cell lysis protein quantification was detected by bicinchoninic acid assay. OXCT1 catalytic activity was calculated as nmol acetoacetyl-CoA formed/minute/milligram whole-cell lysis protein.

### Measurement of HMGCS2 activity

Mitochondria isolation and HMGCS2 enzymatic activity were performed as previously described with slight modifications (39, 40). To assay HMGCS2 activity, mitochondria were obtained from cells transfected with HMGCS2 wildtype or mutant plasmid using the mitochondrial fractionation kit (Proteintech Group, Inc). The enzyme activity was determined by monitoring the conversion of Ac-CoA and AcAc-CoA to 3-hydroxy-3-methylglutaryl-CoA. Assay mixtures (200 μl) contained 100 mM Tris–HCl (pH 8.0), 130 μM dithionitrobenzoic acid, 500 μM acetyl-CoA, and 10 μM acetoacetyl-CoA. Microplate assays were performed using a SpectraMax 96, and the absorbance at 412 nm was recorded every 2 min. The original data were collected every 2 min and lasted for 80 min. The absorbance value at the time point of 10 min was used for enzymatic activity determination. Mitochondrial matrix protein quantification was detected by bicinchoninic acid assay. HMGCS2 catalytic activity was calculated as nmol 3-hydroxy-3-methylglutaryl-CoA formed/minute/milligram mitochondrial matrix protein.

### Measurement of β-HB in cells

The levels of β-HB in cells were measured using the beta-hydroxybutyrate A Assay Kit (Colorimetric) (Abcam; ab83390) according to the manufacturer's protocol. Briefly, the lysates of 2 × 10^6^ cells were obtained and subsequently centrifuged at 12,000 rpm for 5 min at a temperature of 4 °C. The resulting supernatant was collected. A total of 50 μl of Reaction Mix (consisting of β-HB assay buffer, β-HB enzyme mix, and β-HB substrate mix in a ratio of 28:1:1) was added to the tested sample (50 μl), followed by incubation at room temperature for 30 min in the absence of light. The absorbance value at a wavelength of 450 nm was measured, and the concentration of β-HB in the cells was determined using a standard curve.

### Statistical analysis

Experimental data are expressed as mean ± SEM. A two-group comparison was conducted using the unpaired two-tailed Student's *t* test. Multiple groups and/or multiple condition comparisons were performed using one-way or two-way ANOVA. GraphPad Prism software (GraphPad Software, Inc) was used for statistical analysis. A *p* value <0.05 was considered statistically significant. ImageJ software was used for the quantification of the immunoblotting data.

## Data availability

All the data described in this study are contained within the article and supporting information.

## Supporting information

This article contains supporting information (Table S1 and Figs. S1–S3; original images for Western blotting data).

## Conflict of interest

The authors declare that they have no conflicts of interest with the contents of this article.

## Supporting information

Original images for Western blotting data
