# Supplementary material for: β-hydroxybutyrate serves as a regulator in ketone body metabolism through lysine β-hydroxybutyrylation
Source: J Biol Chem. 2025 Apr 2;301(5):108475. doi: 10.1016/j.jbc.2025.108475 (PMC12147175; doi:10.1016/j.jbc.2025.108475)

Original images for Western blotting data

Fig 1A

IP:OXCT1 IB:Kbhb

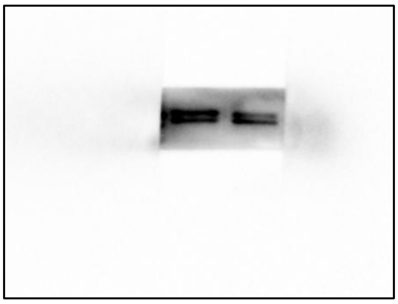

IP:OXCT1 IB:OXCT1

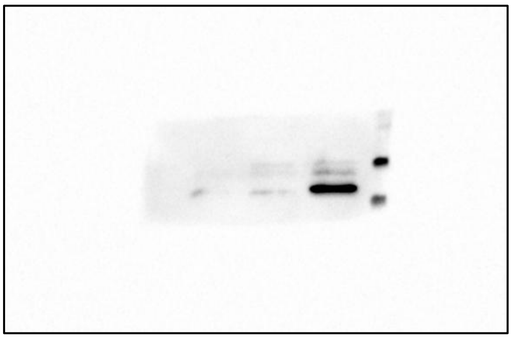

Input:OXCT1

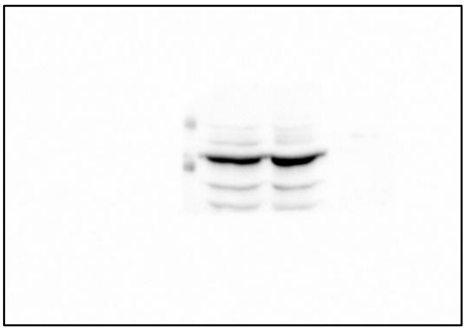

Fig 1B

IB:Kbhb

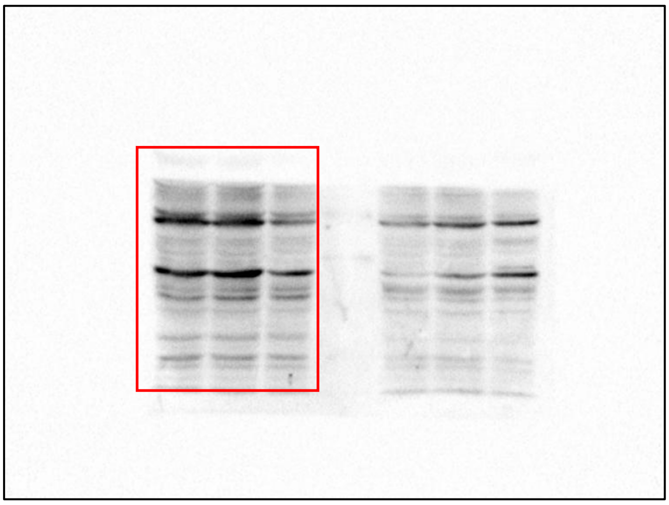

IB:OXCT1

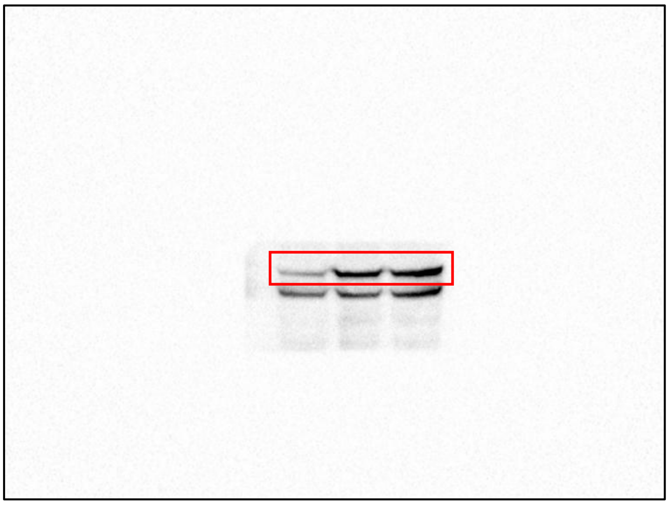

IB:α-tubulin

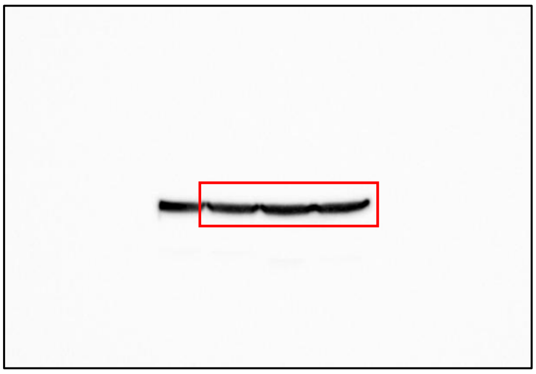

Fig 1D

IB:Kbhb

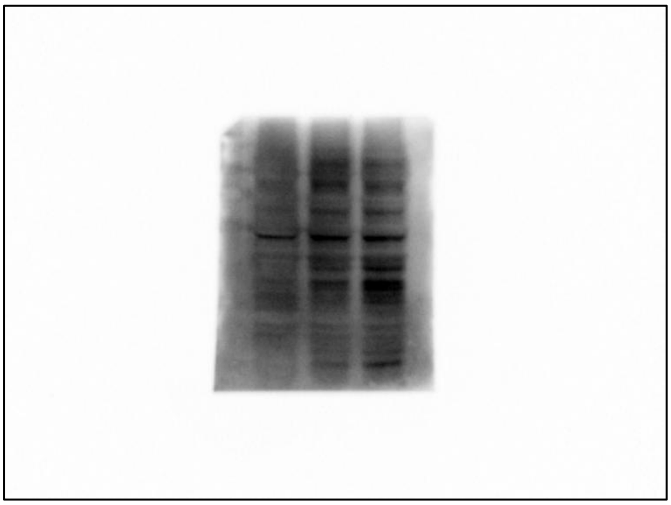

IB:OXCT1

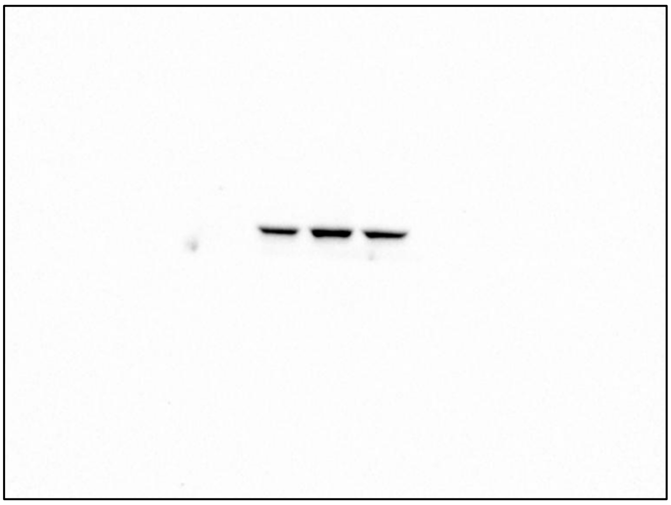

IB:β-actin

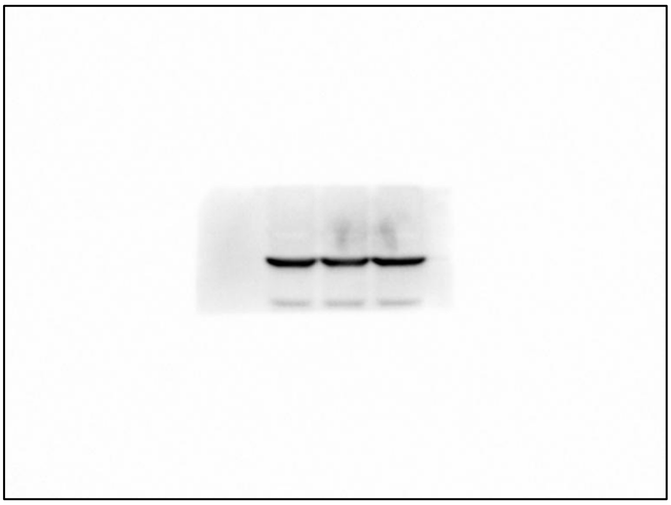

IB:Kbhb BEAS-2B

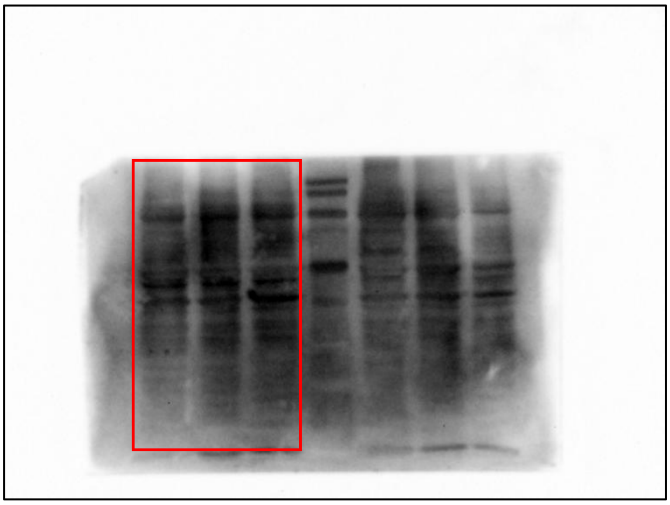

IB:OXCT1 BEAS-2B

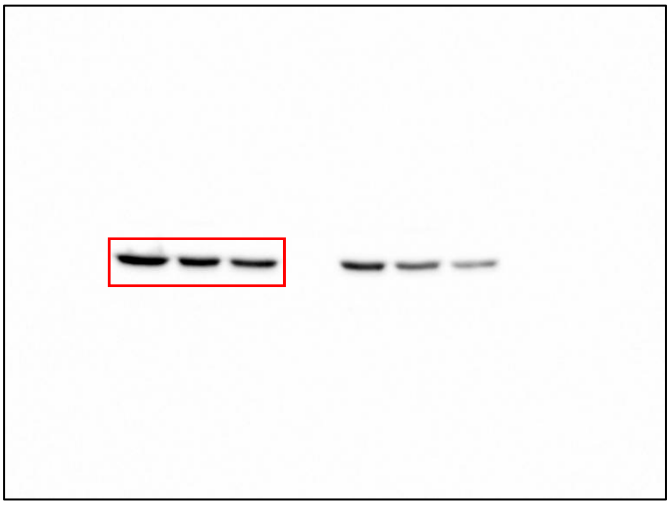

IB:β-actin BEAS-2B

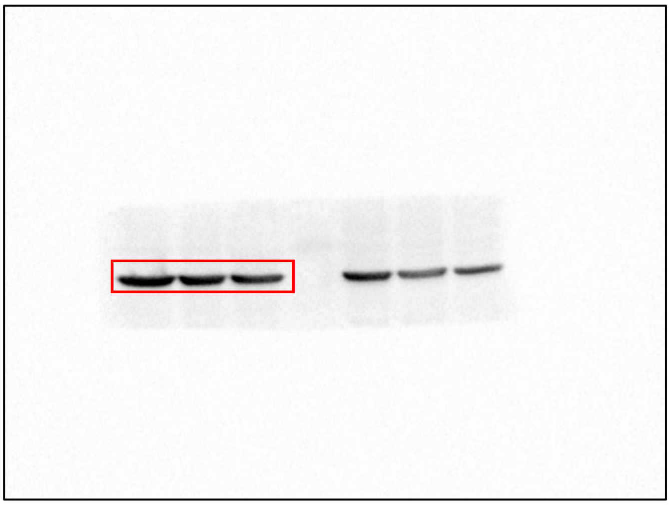

IB:Kbhb H1299

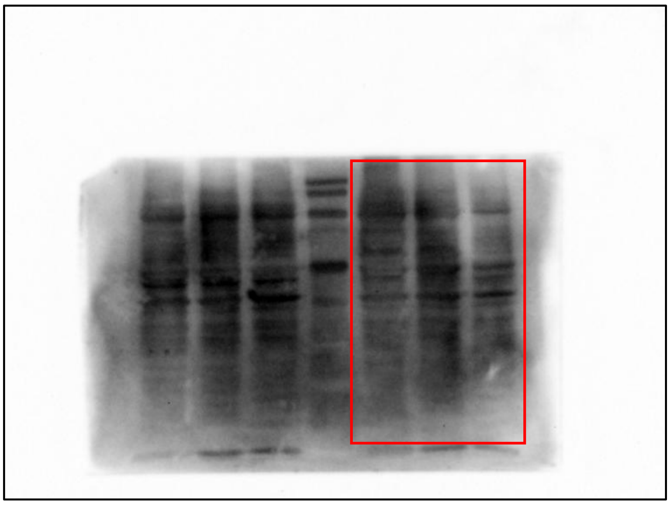

IB:OXCT1 H1299

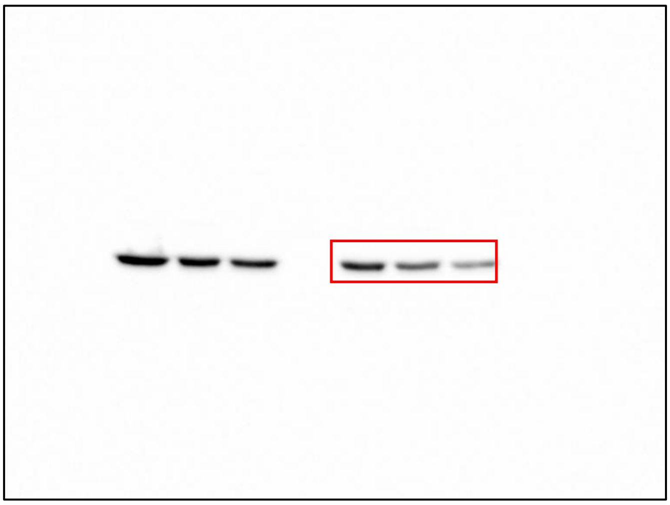

IB:β-actin H1299

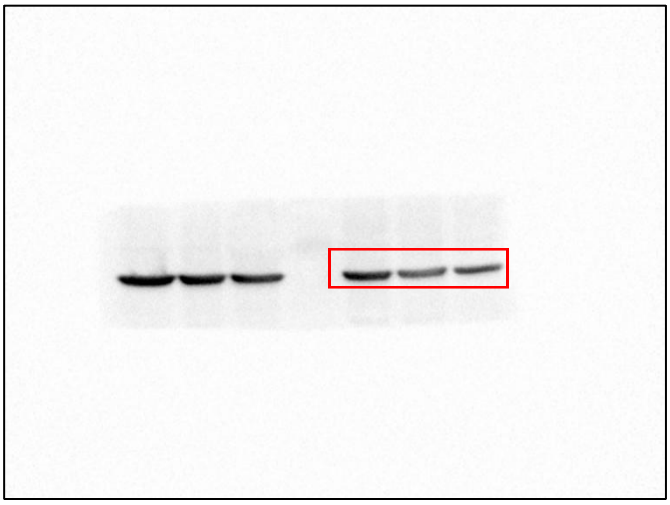

Fig 1E

IP:OXCT1 IB:Kbhb

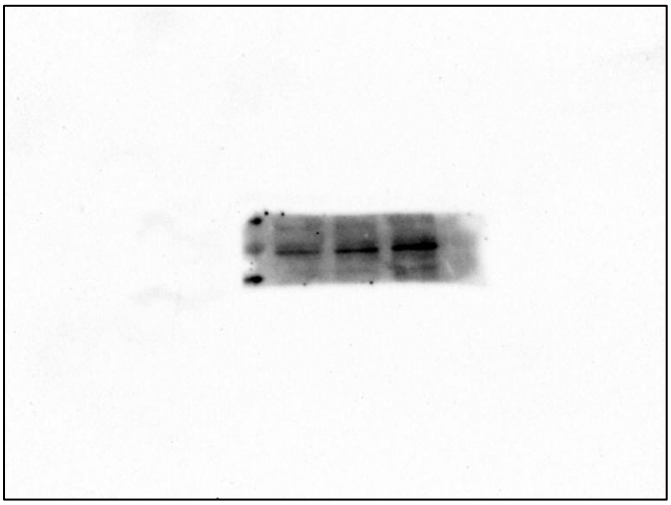

IP:OXCT1 IB:OXCT1

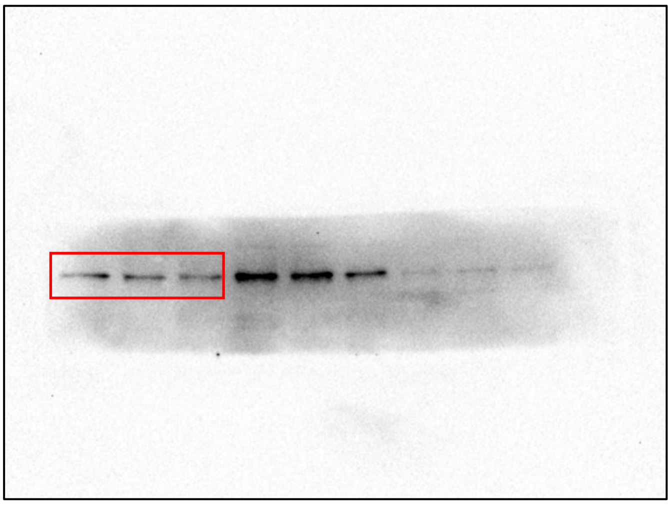

Input:OXCT1

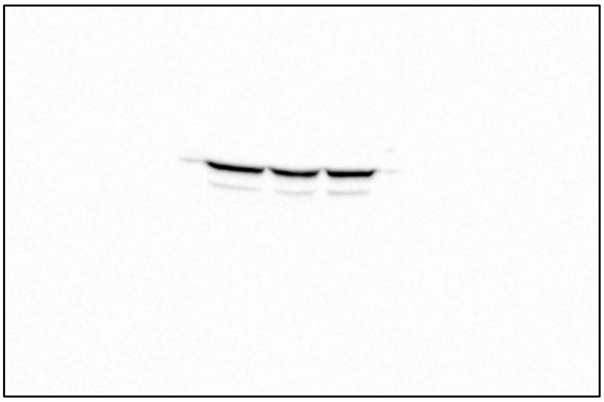

IP:OXCT1 IB:Kbhb BEAS-2B

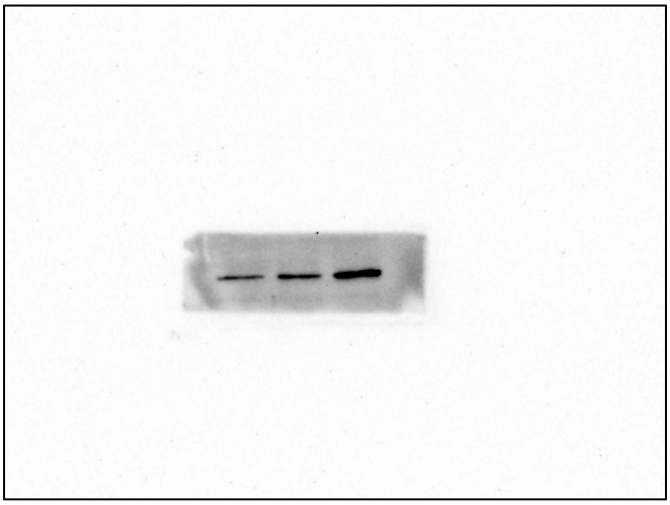

IP:OXCT1 IB: OXCT1 BEAS-2B

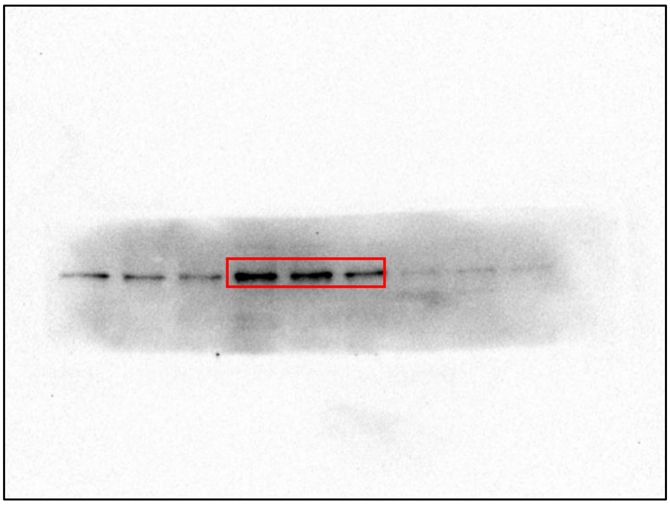

Input:OXCT1 BEAS-2B

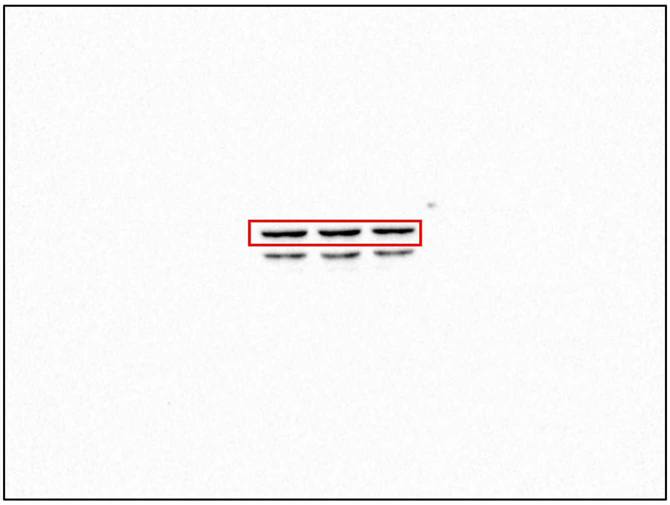

IP:OXCT1 IB:Kbhb H1299

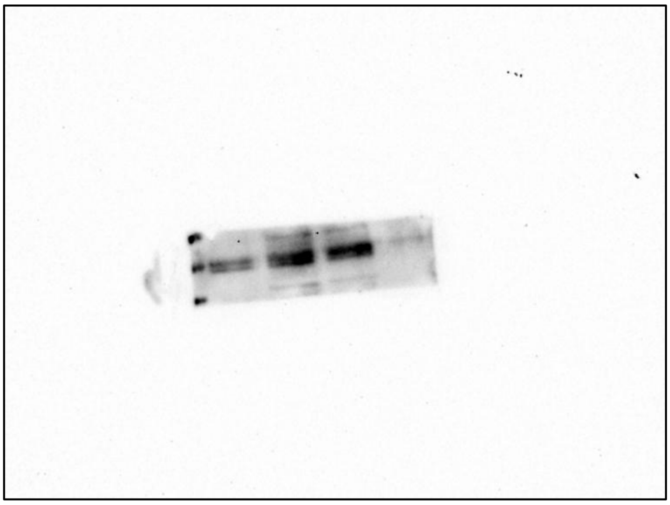

IP:OXCT1 IB: OXCT1 H1299

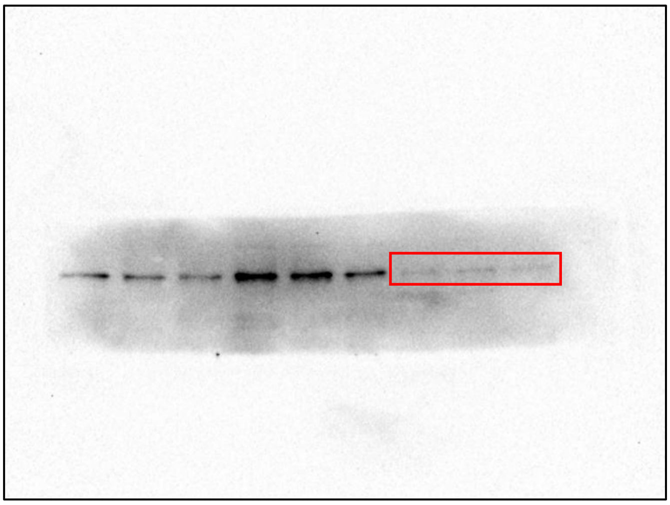

Input:OXCT1 H1299

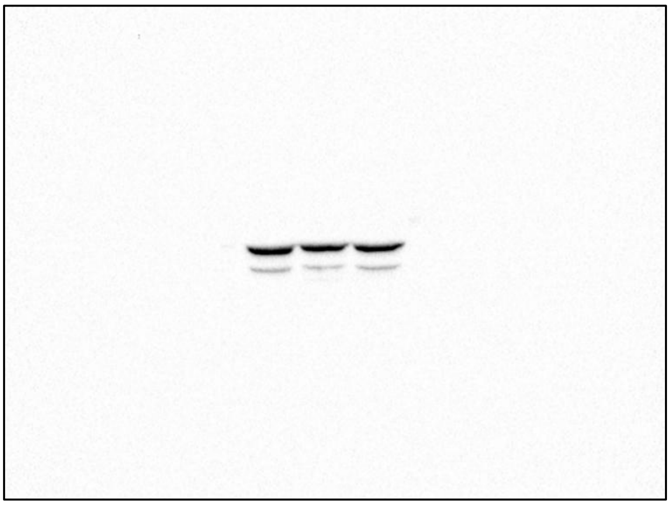

Fig 2A

IP:OXCT1 IB:Kbhb

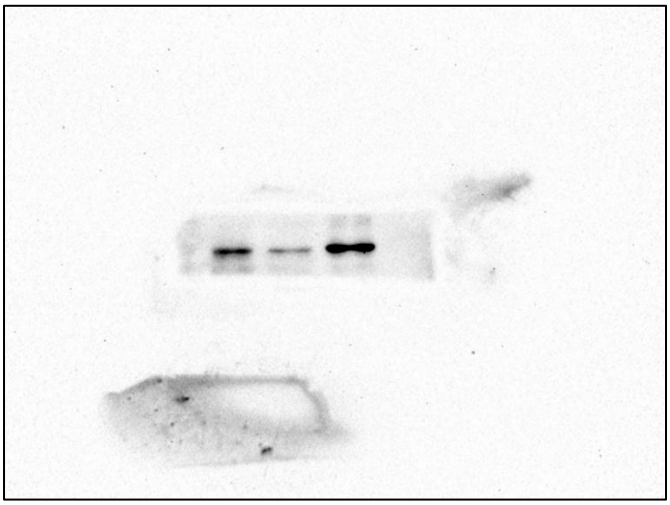

IP:OXCT1 IB:OXCT1

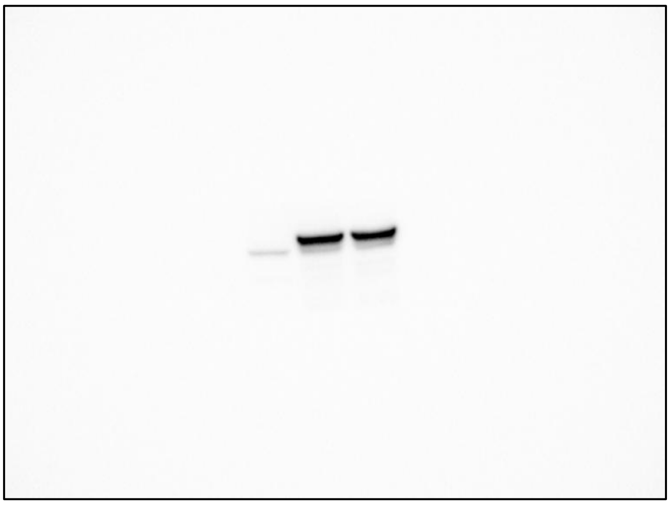

Input:OXCT1

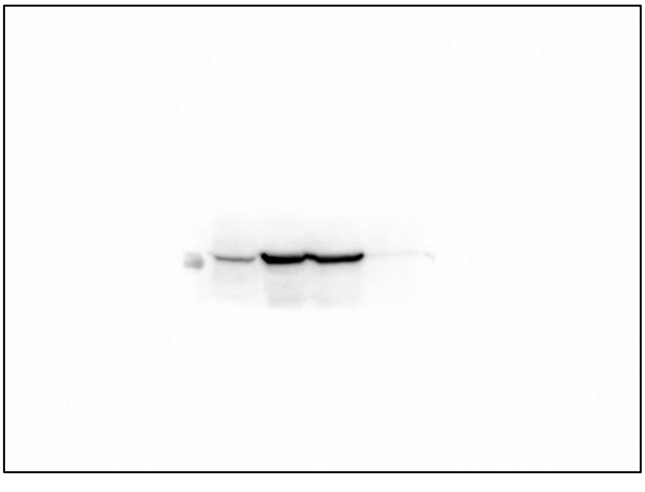

Fig 2B

IP:HMGCS2 IB:Kbhb

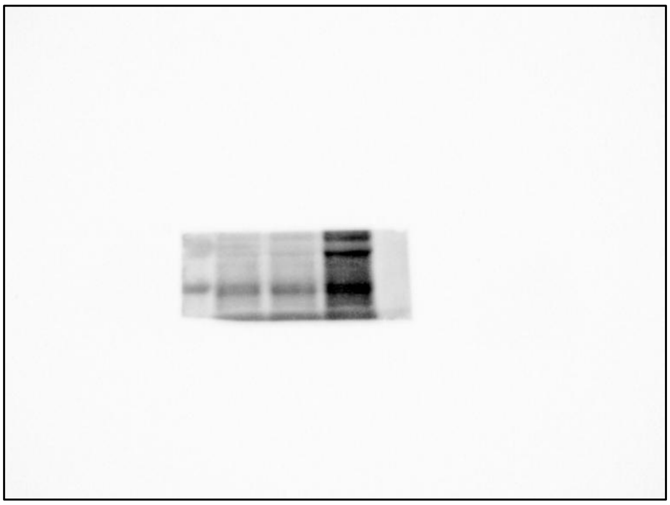

IP:HMGCS2 IB:HMGCS2

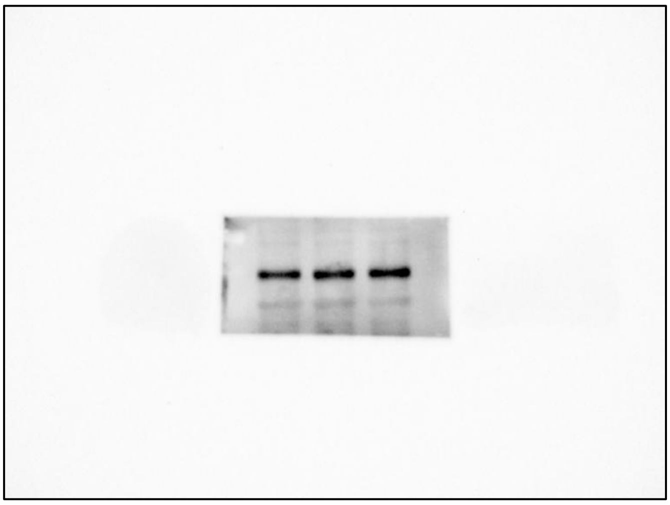

Input:HMGCS2

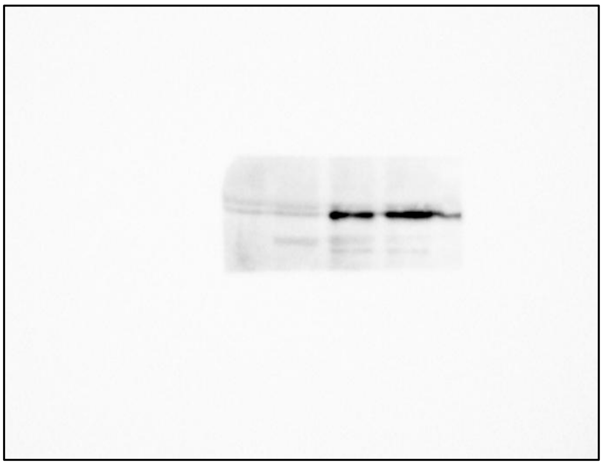

Fig 2C

IP:HMGCS2 IB:Kbhb

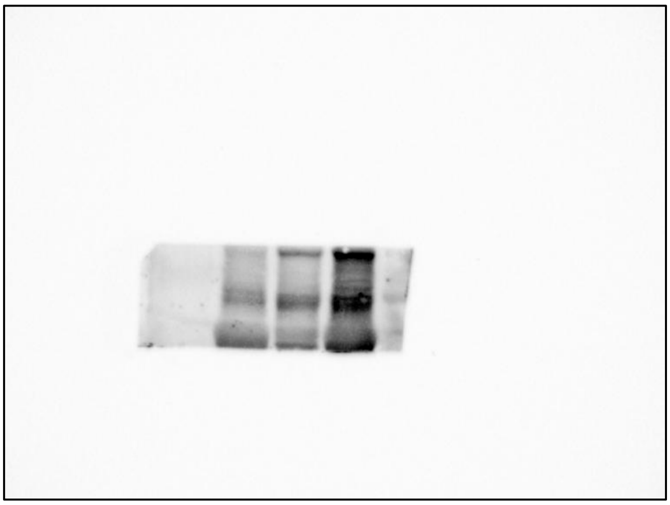

IP:HMGCS2 IB:HMGCS2

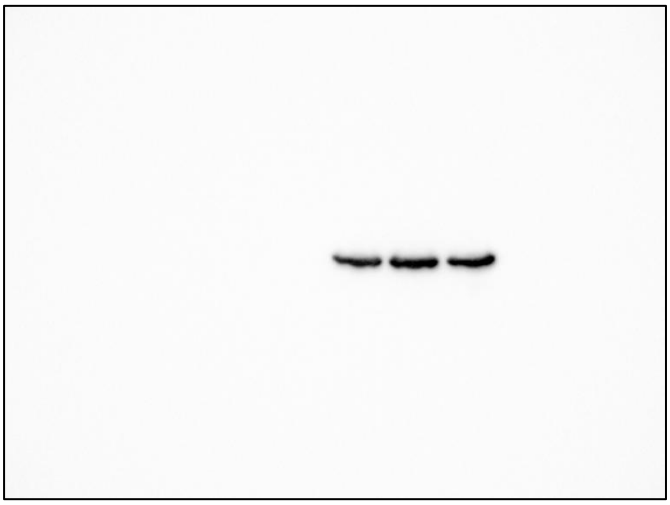

Input:HMGCS2

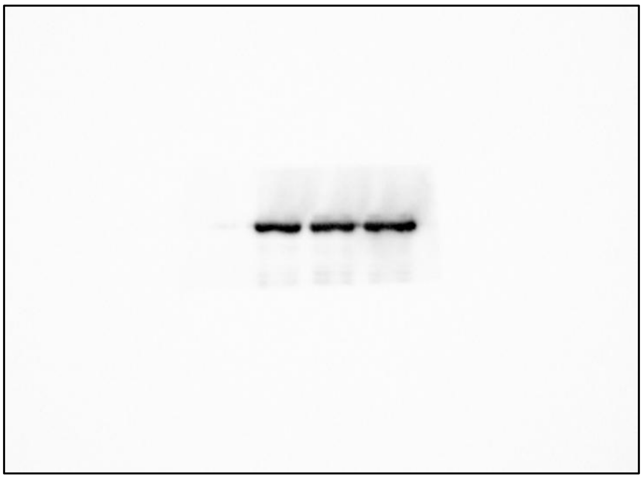

Fig 2E

Kbhb

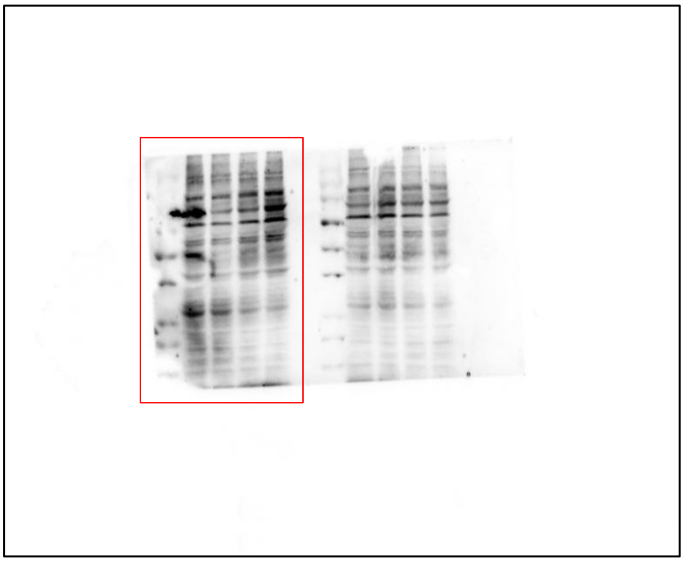

OXCT1

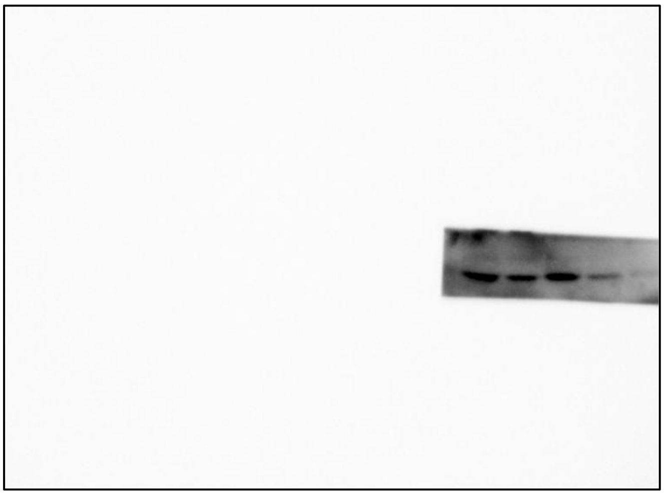

β-actin

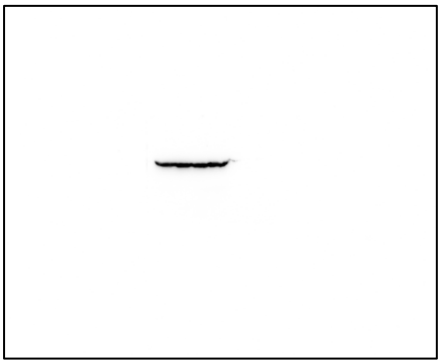

Fig 2F

Kbhb

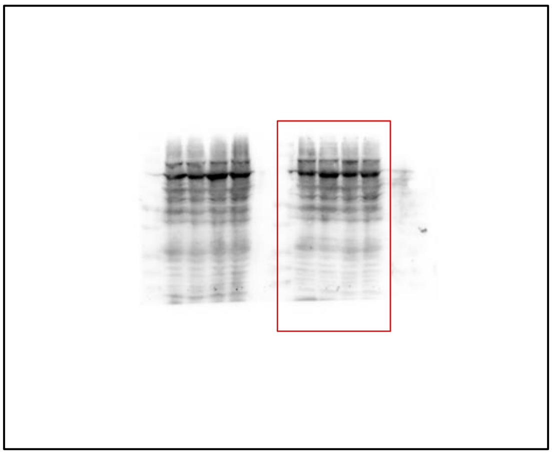

HMGCS2

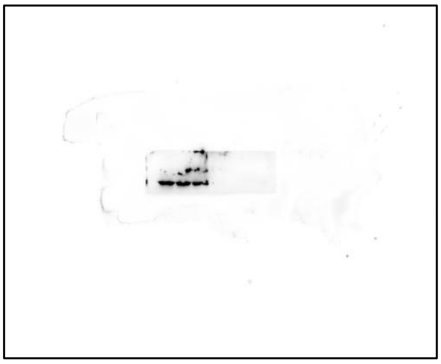

β-actin

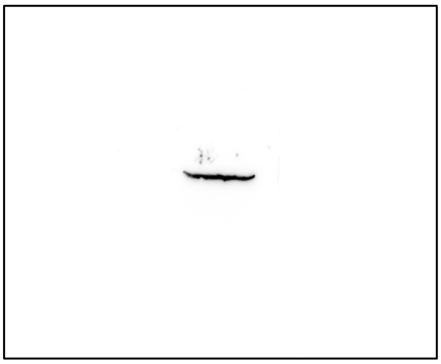

Fig 3E

IB:Kbhb Kidney

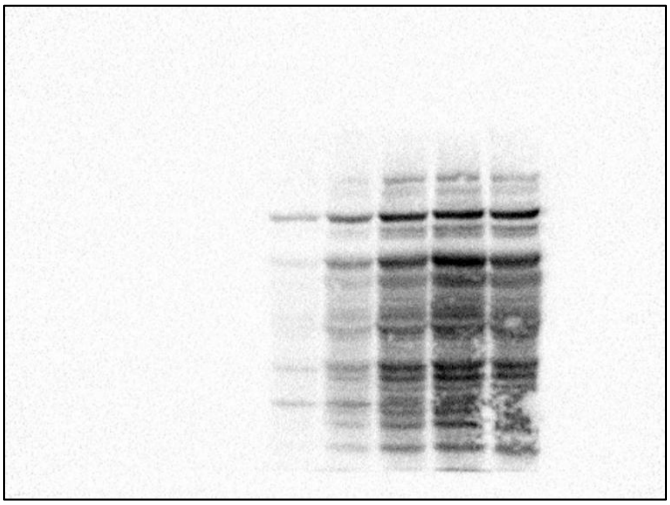

IB:OXCT1 Kidney

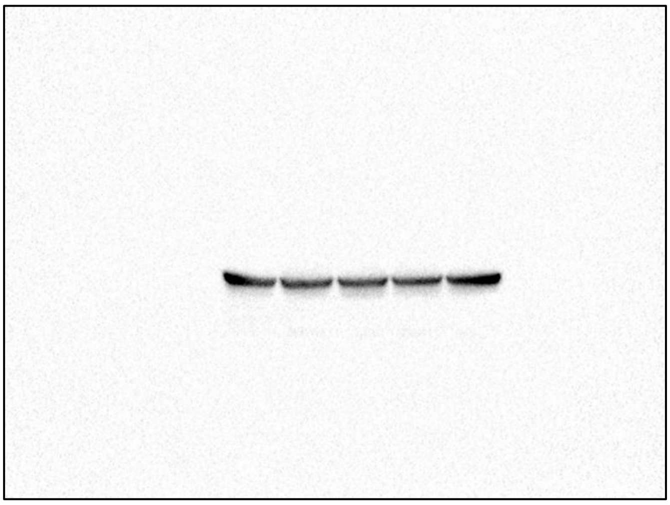

IB:α-tubulin Kidney

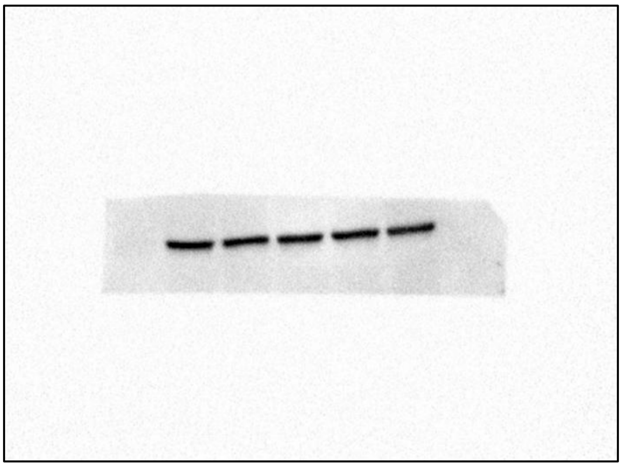

IB:Kbhb Heart

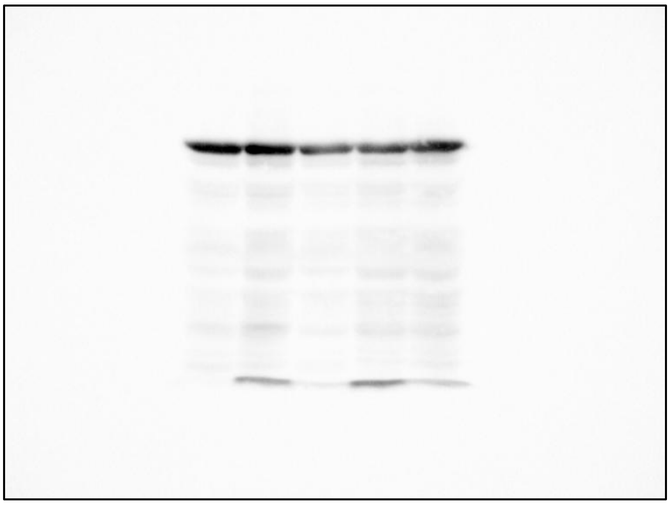

IB:OXCT1 Heart

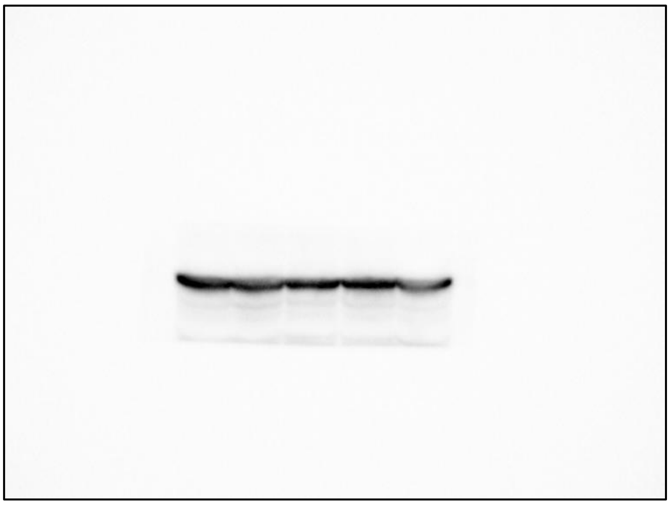

IB:α-tubulin Heart

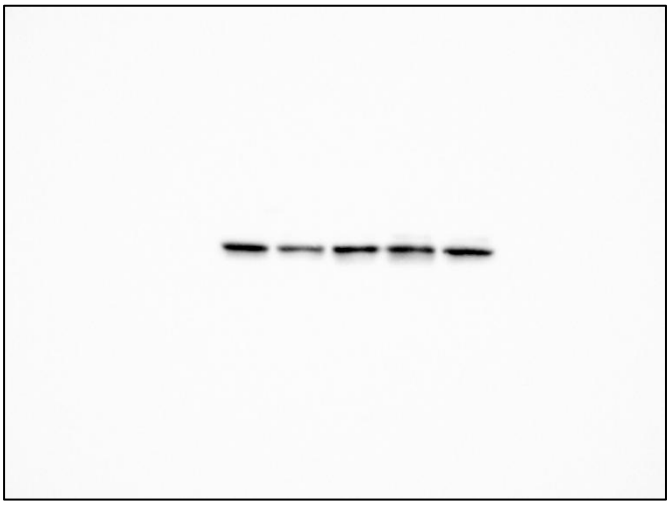

Fig 3F

IB:Kbhb Liver

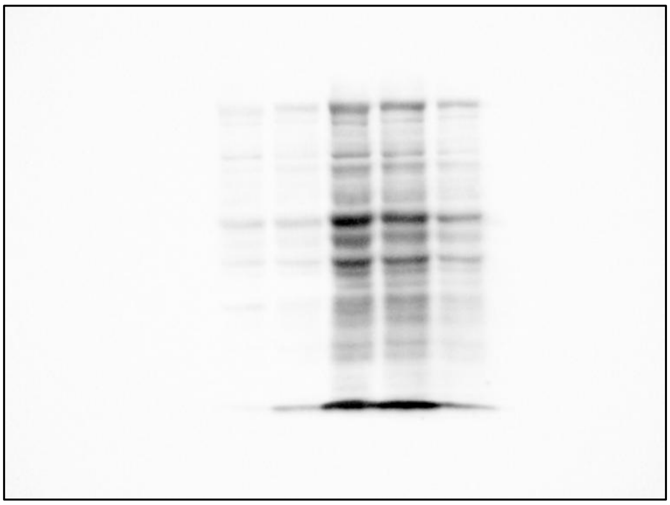

IB:OXCT1 Liver

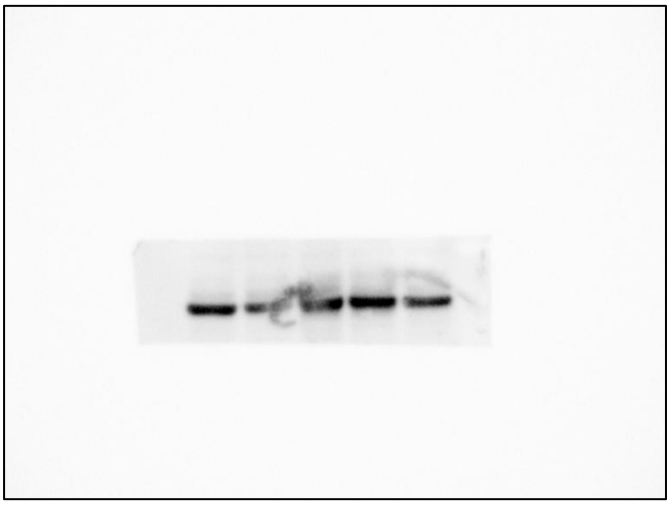

IB:α-tubulin Liver

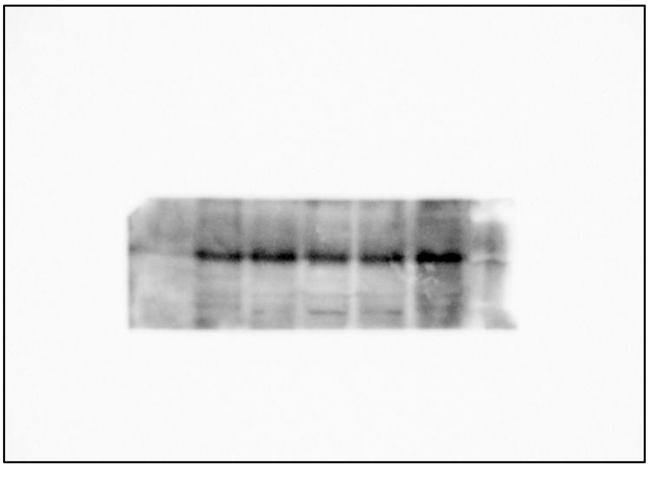

Fig 3G

IP:OXCT1 IB:Kbhb Kidney

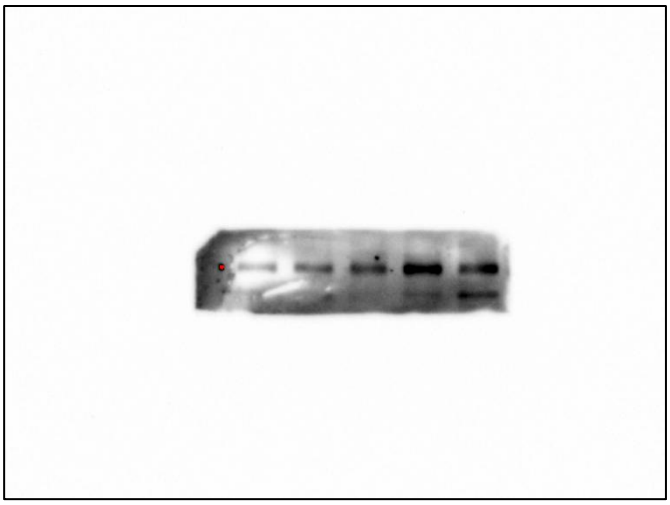

IP:OXCT1 IB:OXCT1 Kidney

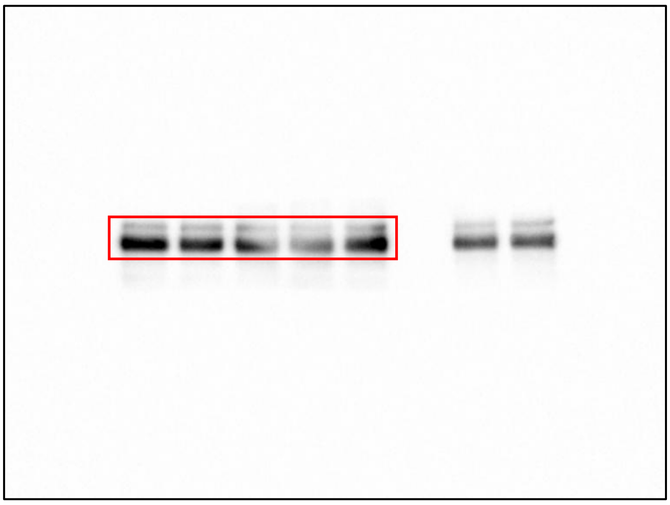

Input:OXCT1 Kidney

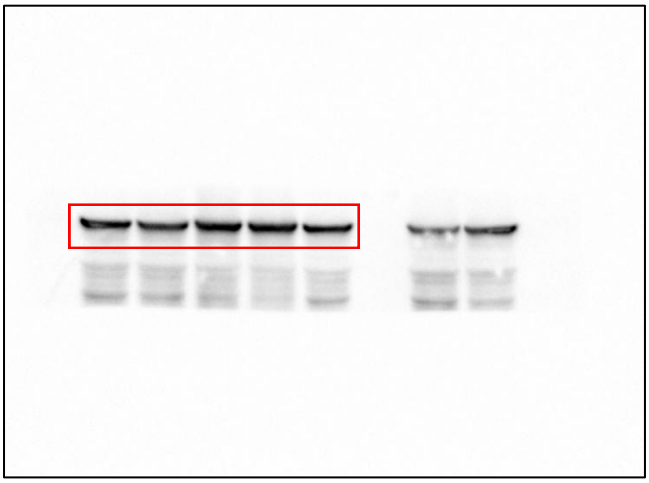

Fig 3H

IP:HMGCS2 IB:Kbhb Liver

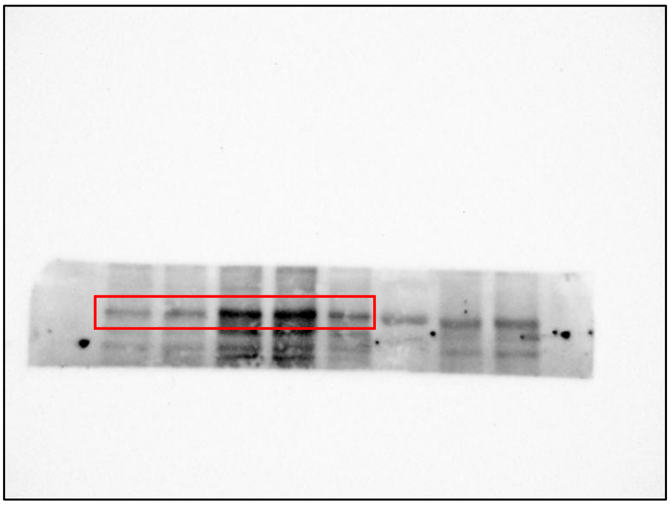

IP: HMGCS2 IB: HMGCS2 Liver

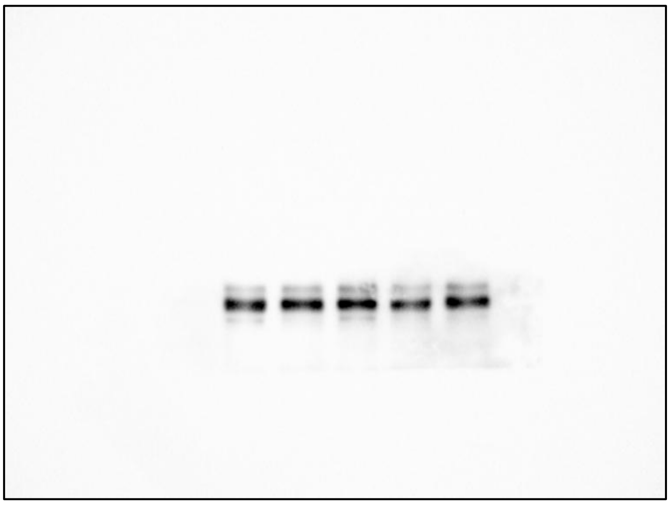

Input: HMGCS2 Liver

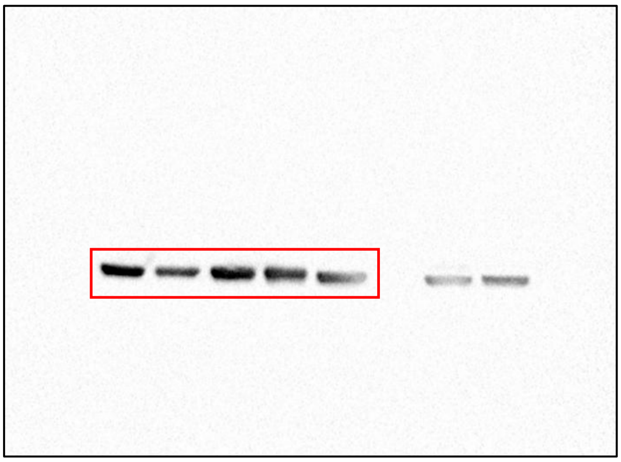

Fig 4C

IB:Kbhb Skeletal Muscle

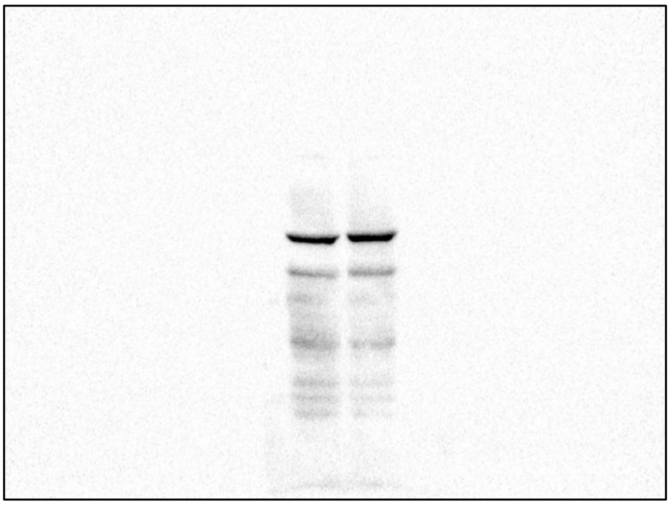

IB:OXCT1 Skeletal Muscle

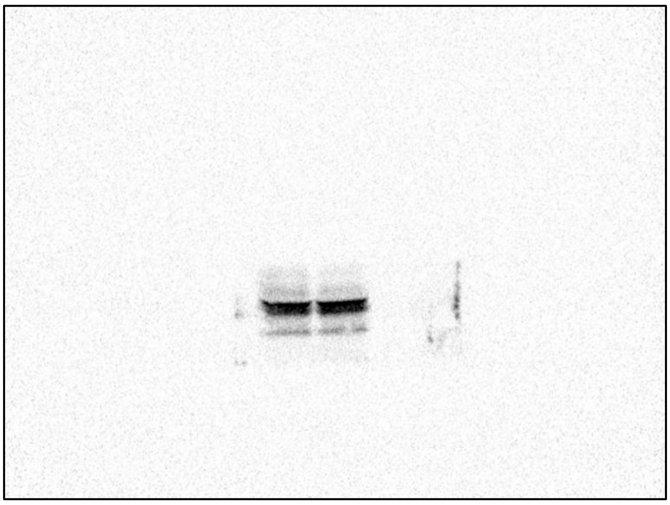

IB:α-tubulin Skeletal Muscle

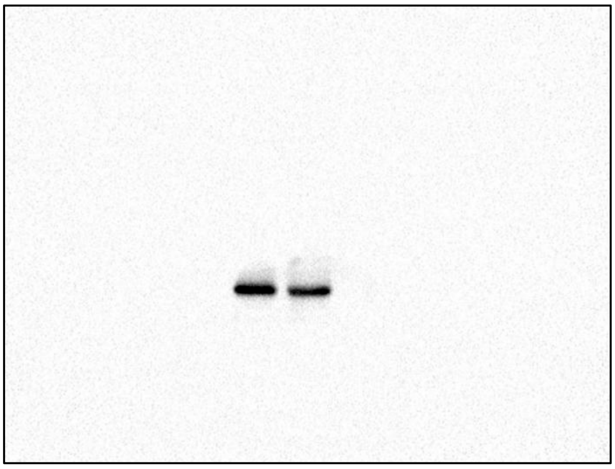

IB:Kbhb Brain

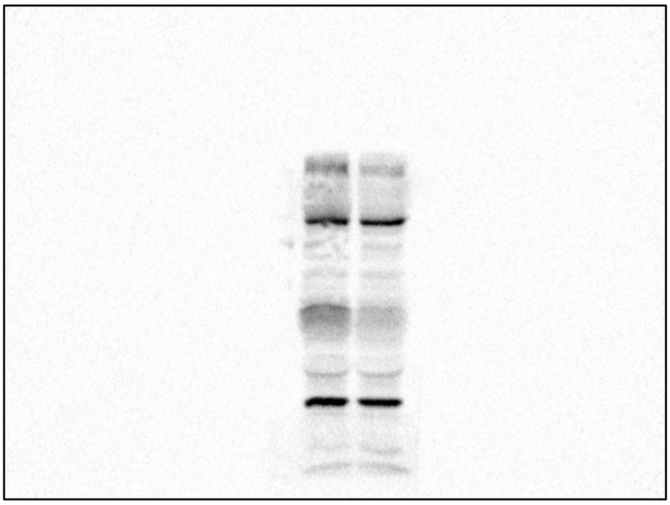

IB:OXCT1 Brain

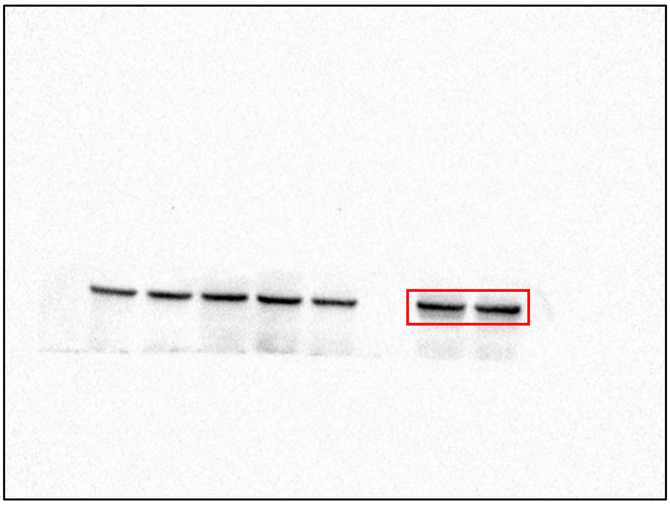

IB:α-tubulin Brain

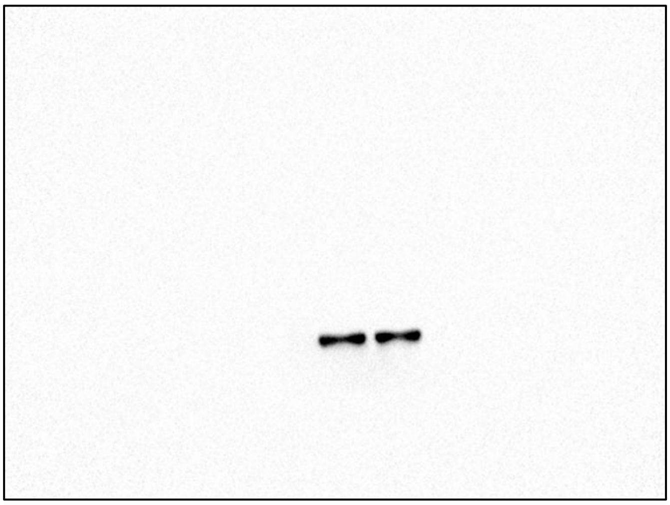

IB:Kbhb Kidney

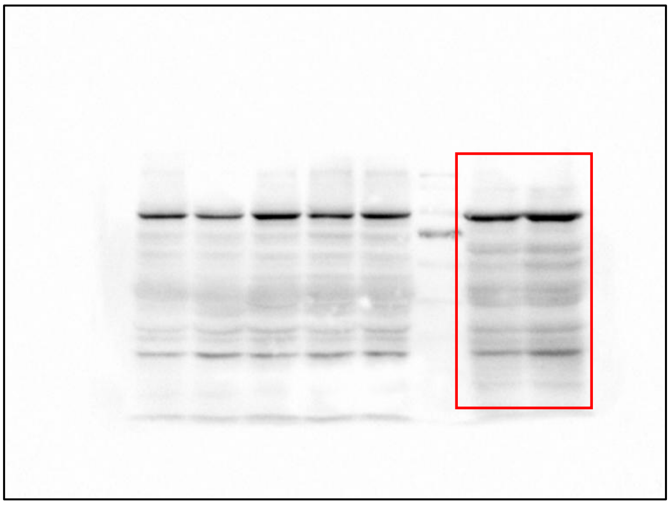

IB:OXCT1 Kidney

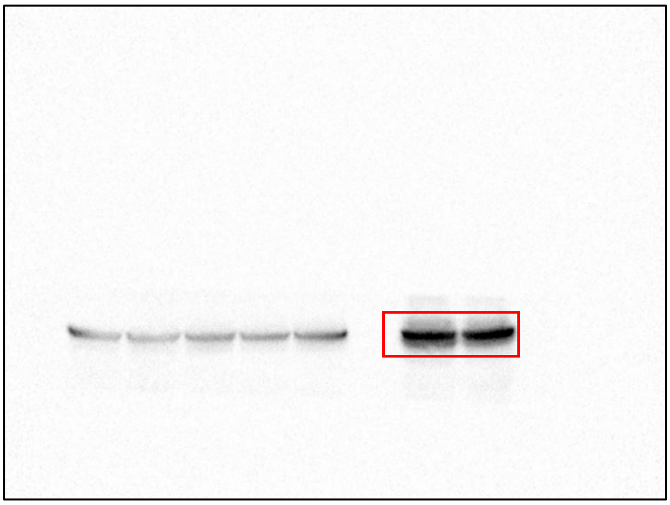

IB:α-tubulin Kidney

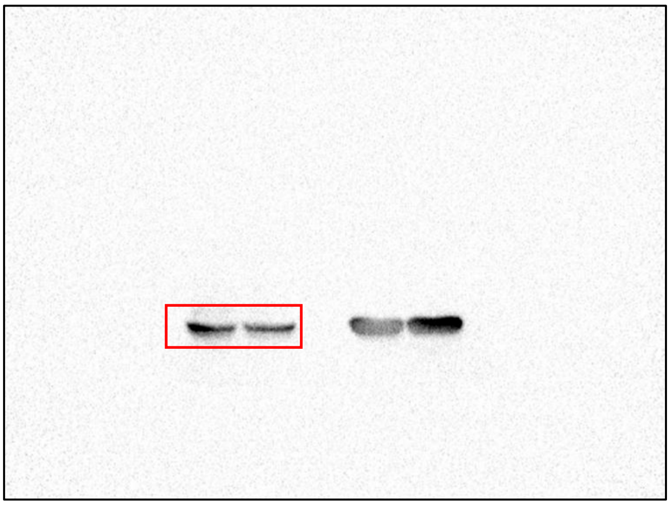

IB:Kbhb Heart

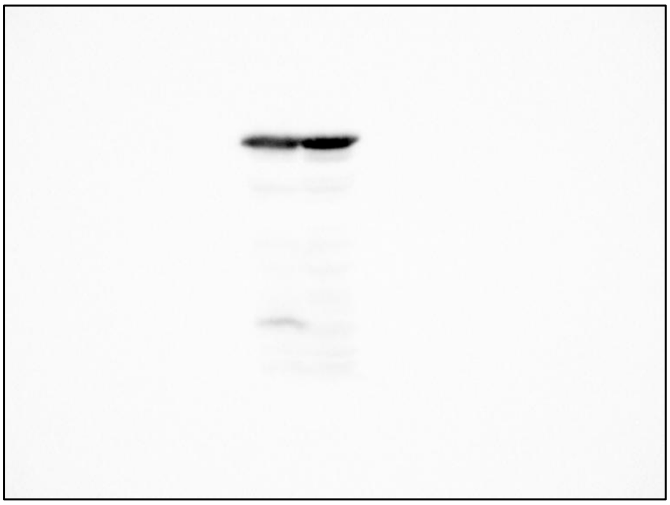

IB:OXCT1 Heart

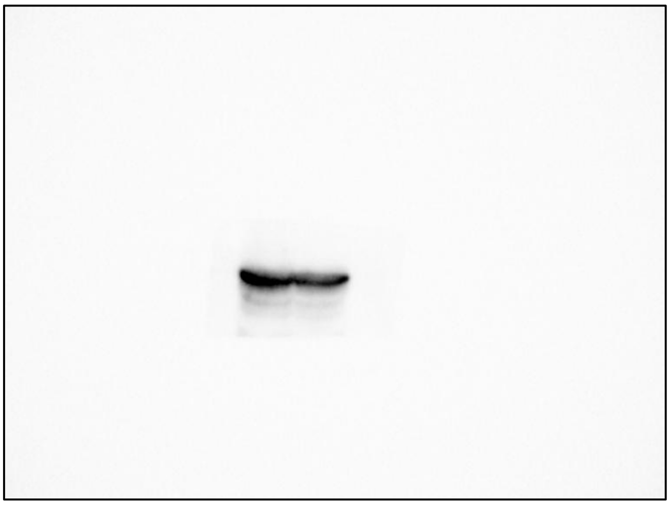

IB:α-tubulin Heart

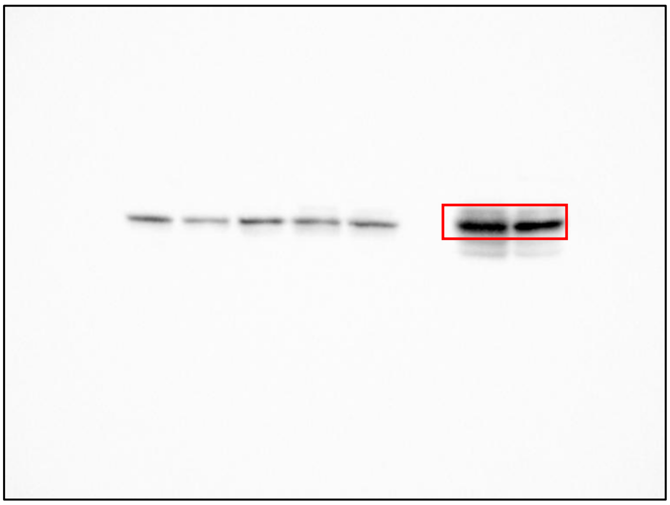

Fig 4D

IB:Kbhb Liver

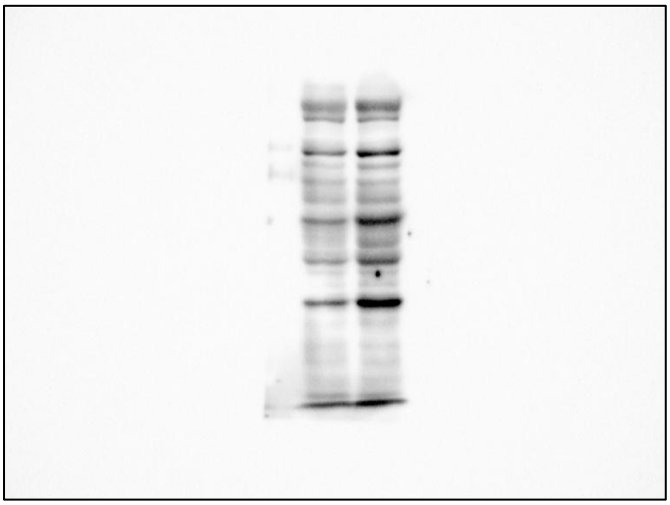

IB:OXCT1 Liver

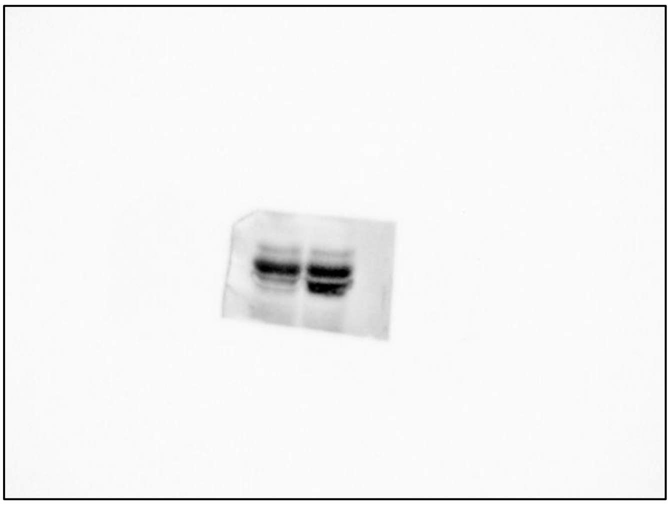

IB:α-tubulin Liver

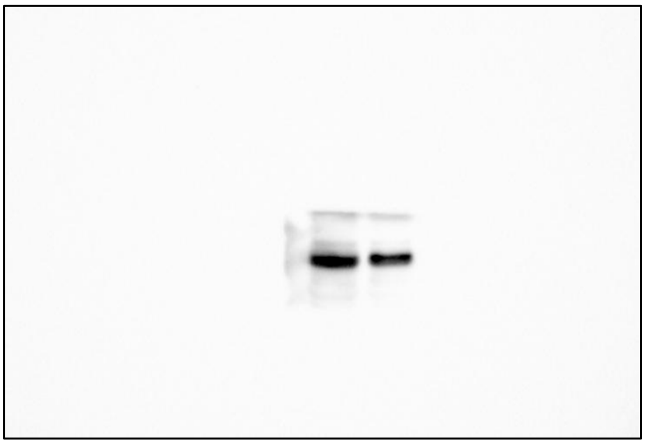

Fig 4E

IP:OXCT1 IB:Kbhb Skeletal Muscle

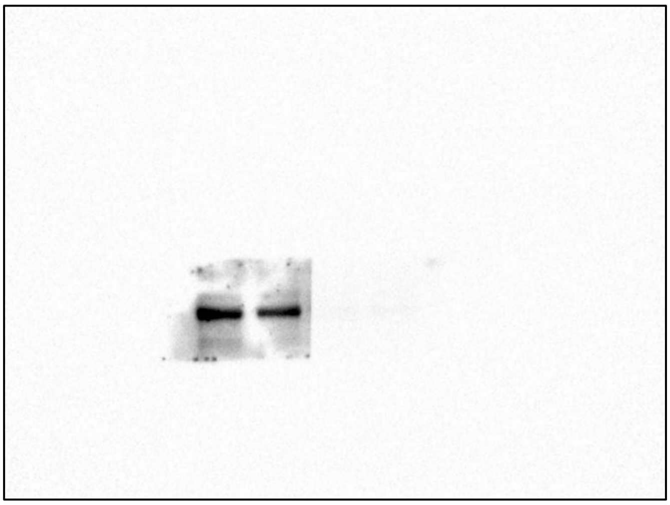

IP:OXCT1 IB:OXCT1 Skeletal Muscle

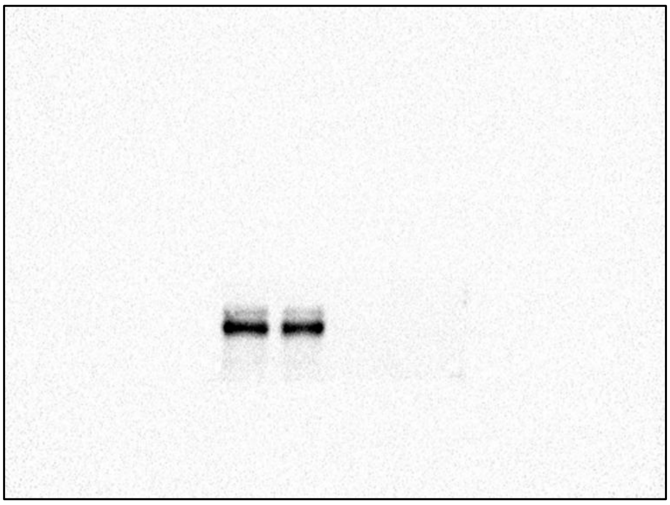

Input:OXCT1 Skeletal Muscle

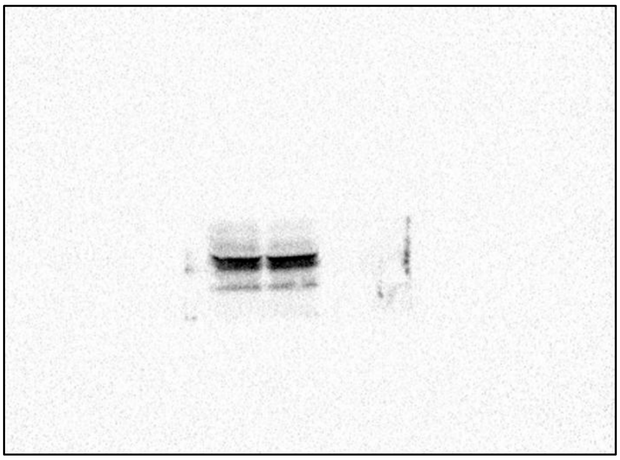

IP:OXCT1 IB:Kbhb Brain

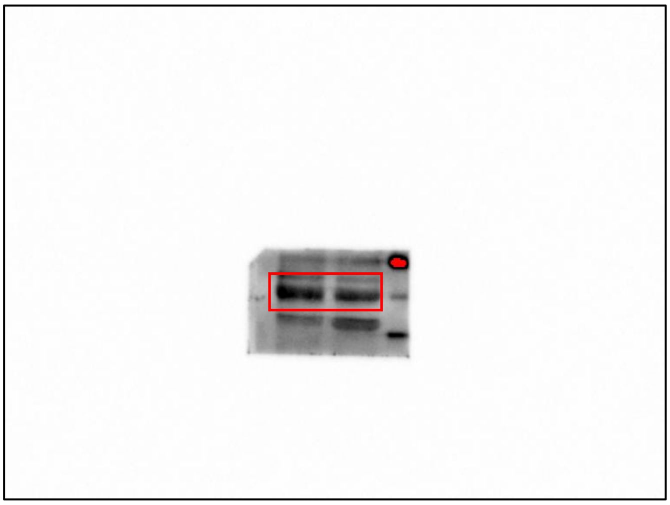

IP:OXCT1 IB:OXCT1 Brain

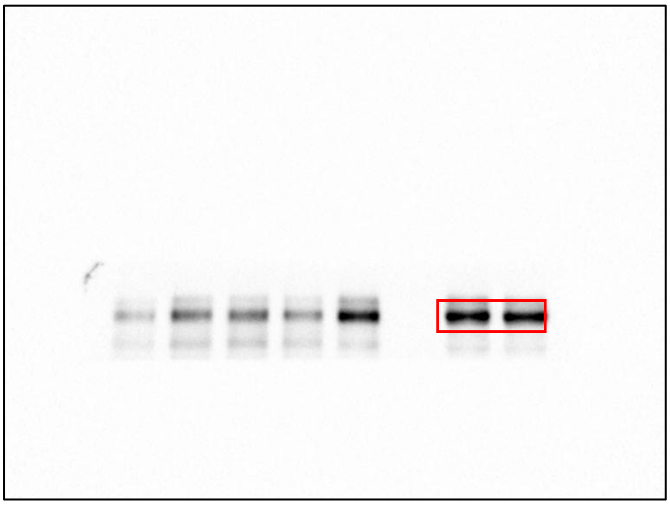

Input:OXCT1 Brain

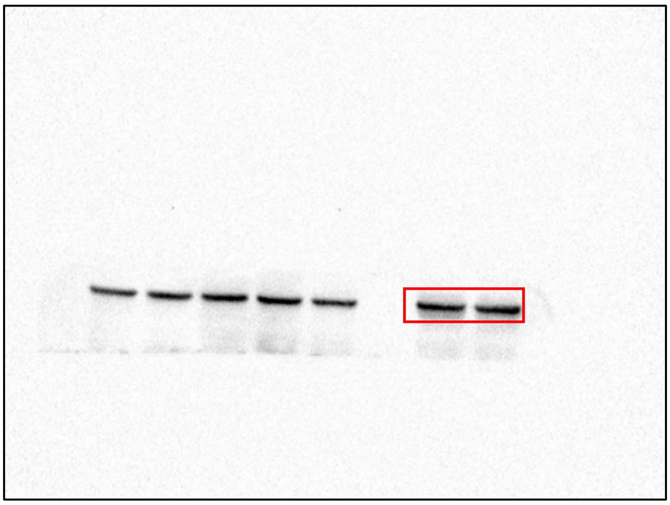

IP:OXCT1 IB:Kbhb Kidney

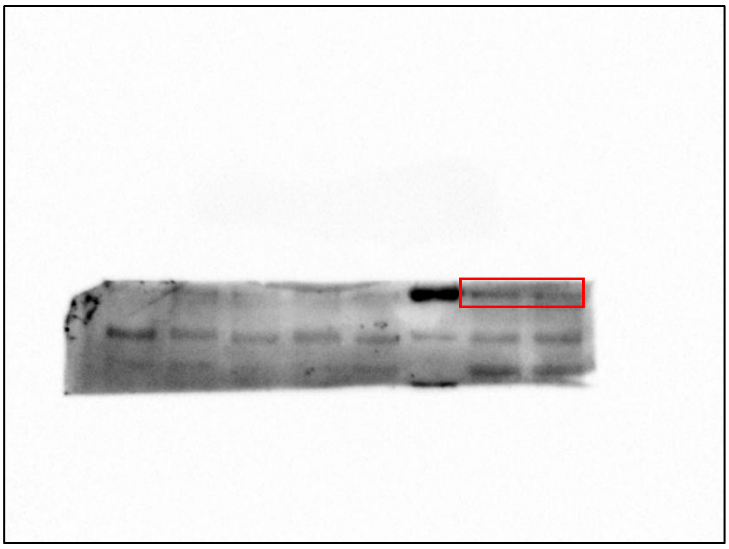

IP:OXCT1 IB:OXCT1 Kidney

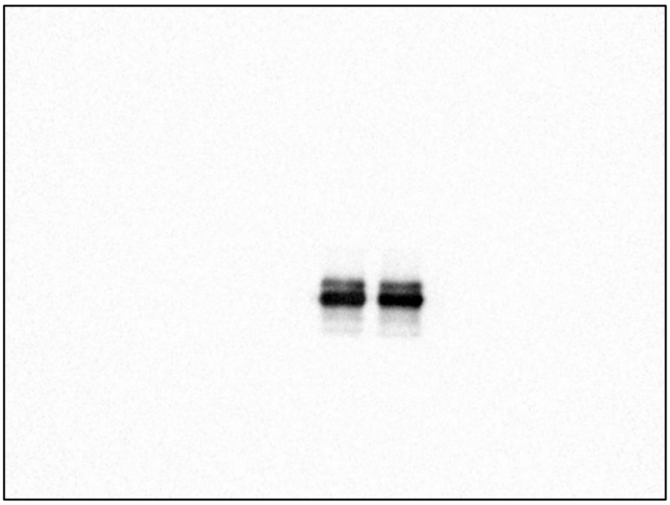

Input:OXCT1 Kidney

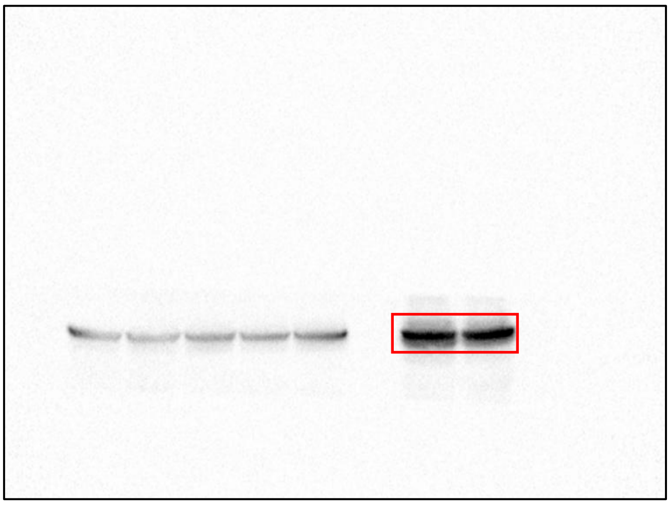

IP:OXCT1 IB:Kbhb Heart

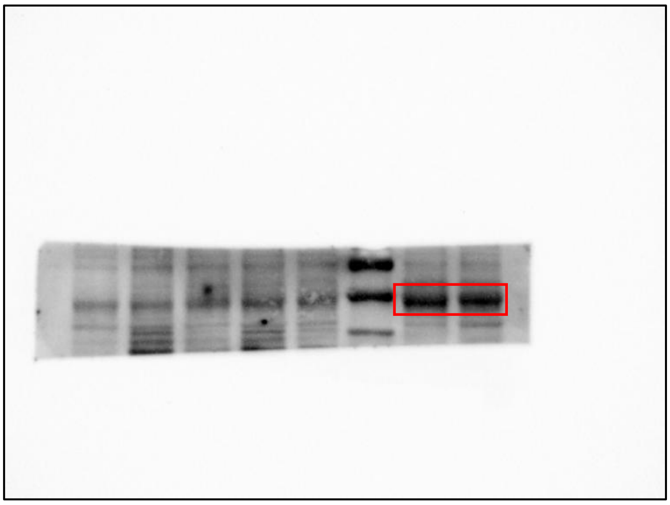

IP:OXCT1 IB:OXCT1 Heart

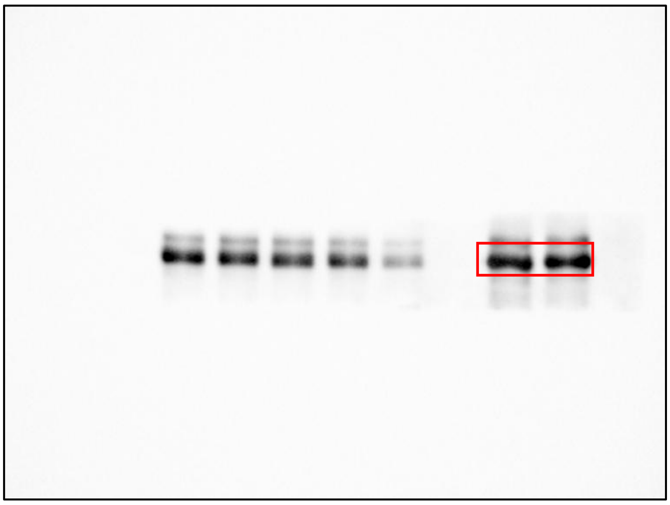

Input:OXCT1 Heart

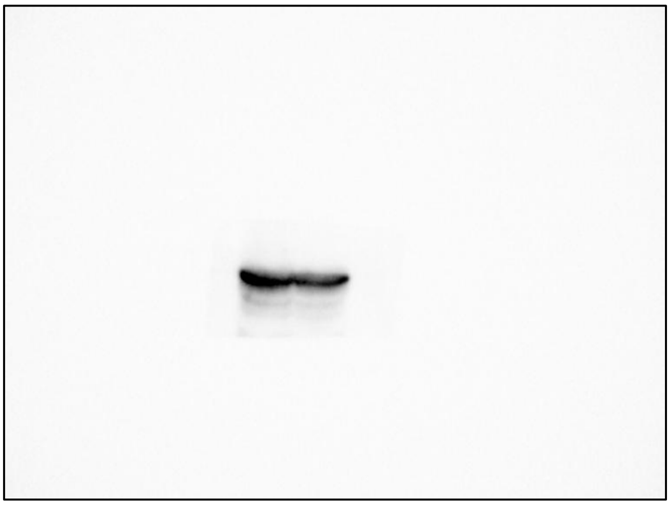

Fig 4F

IP:HMGCS2 IB:Kbhb Liver

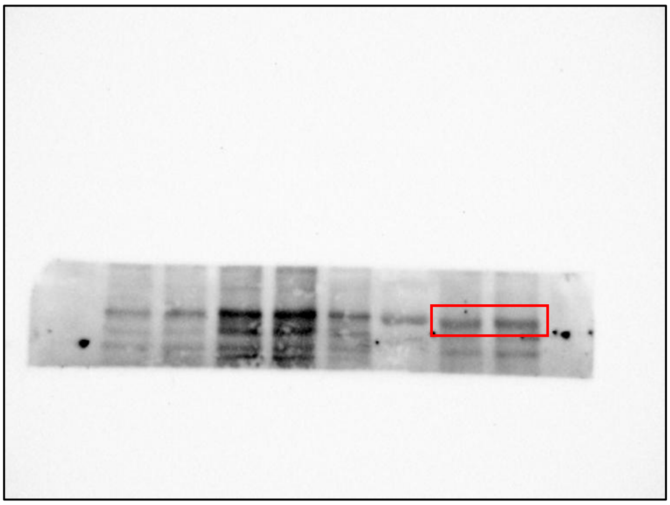

IP: HMGCS2 IB: HMGCS2 Liver

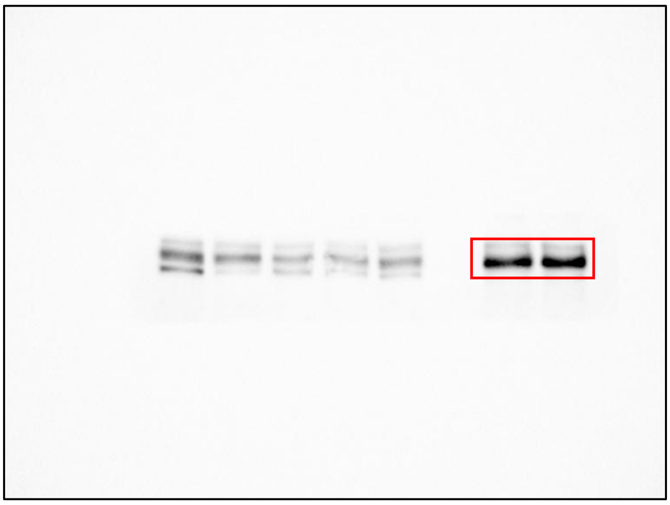

Input: HMGCS2 Liver

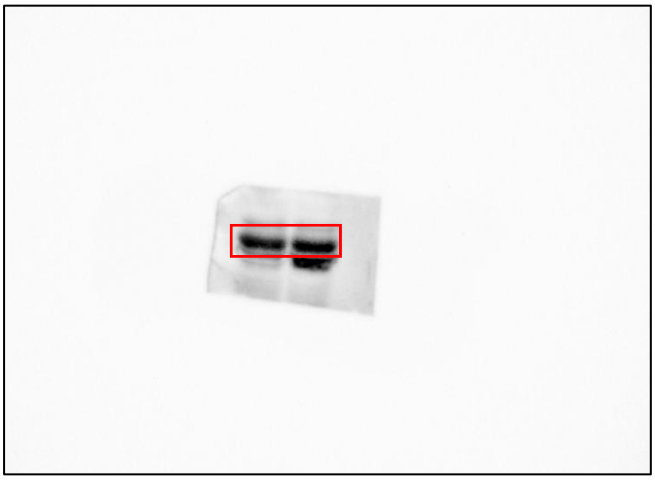

IP:HMGCS2 IB:Kbhb Kidney

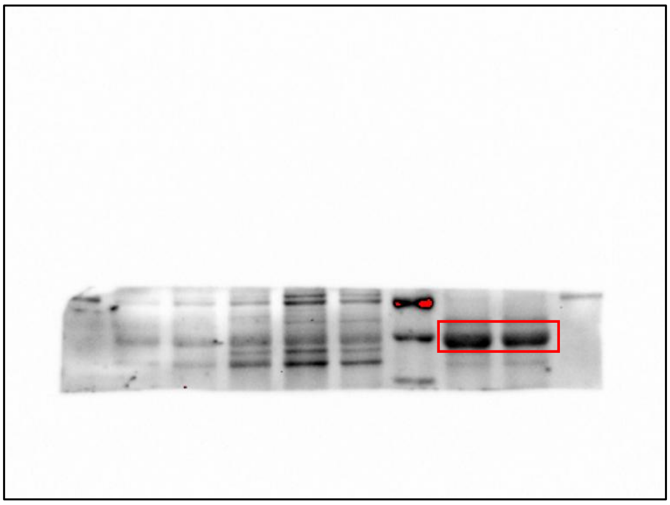

IP: HMGCS2 IB: HMGCS2 Kidney

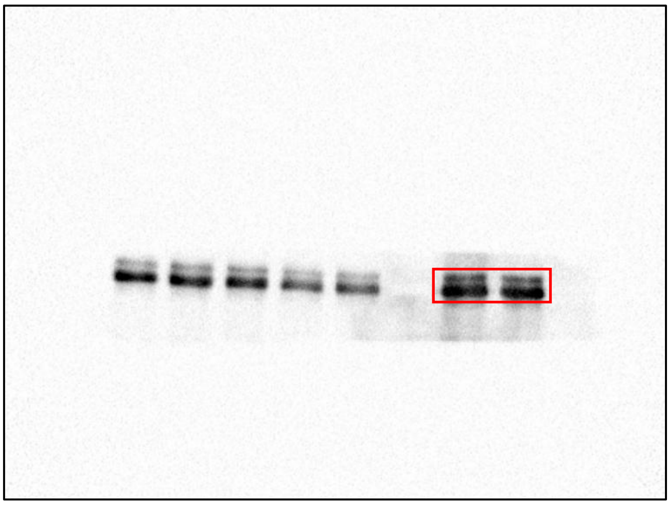

Input: HMGCS2 Kidney

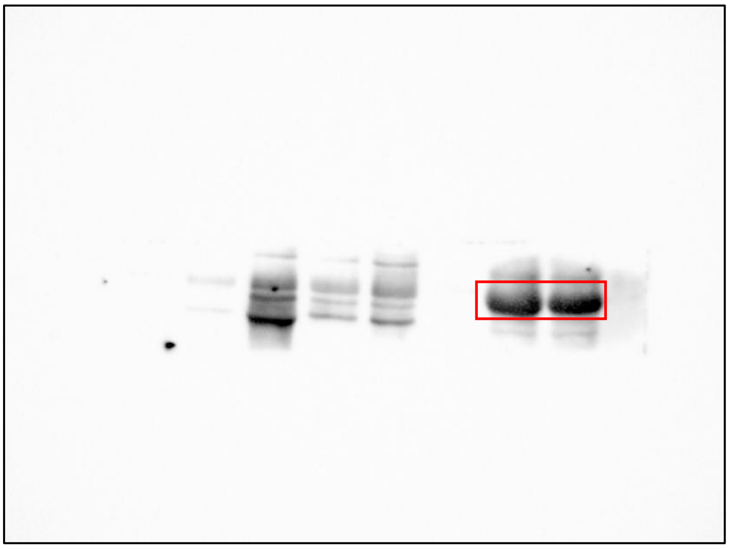

Fig 5C

IP:Flag IB:Kbhb

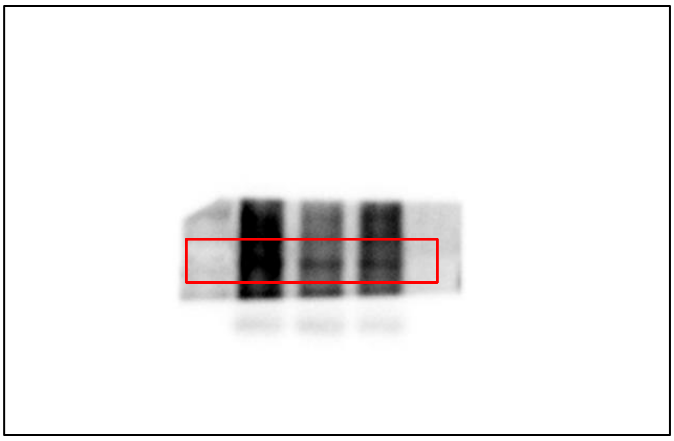

IP:Flag IB:OXCT1

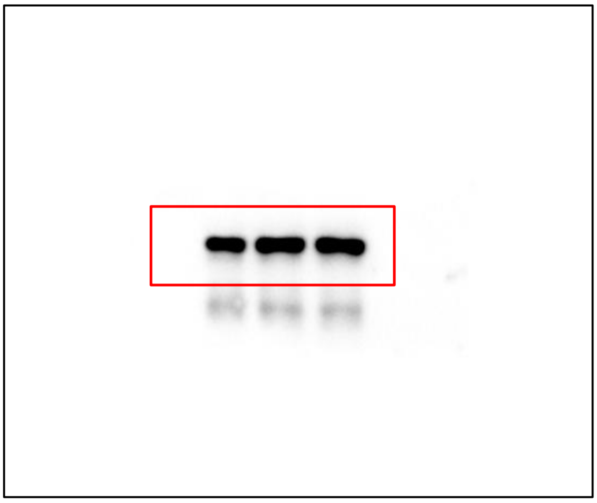

Fig 5D

IP:Flag IB:Kbhb

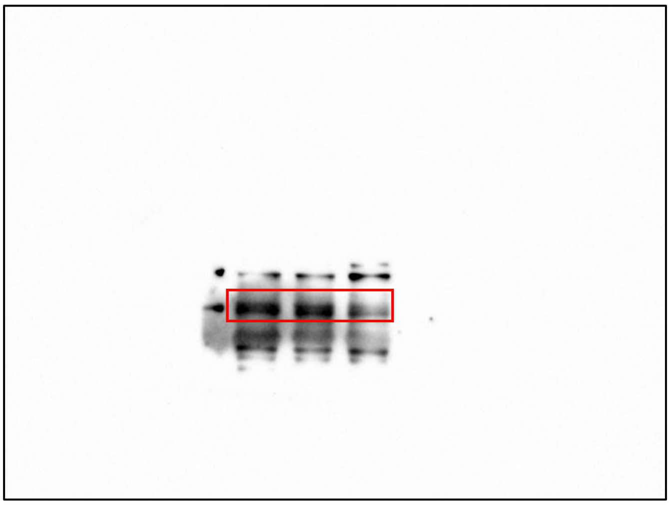

IP:Flag IB:Flag

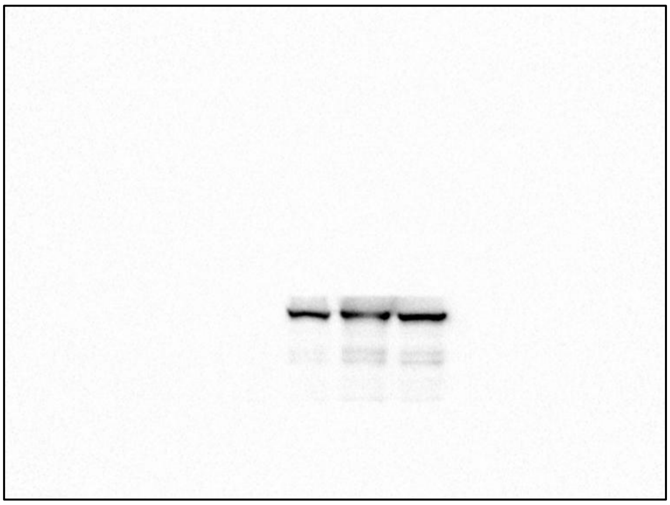

Input:Flag

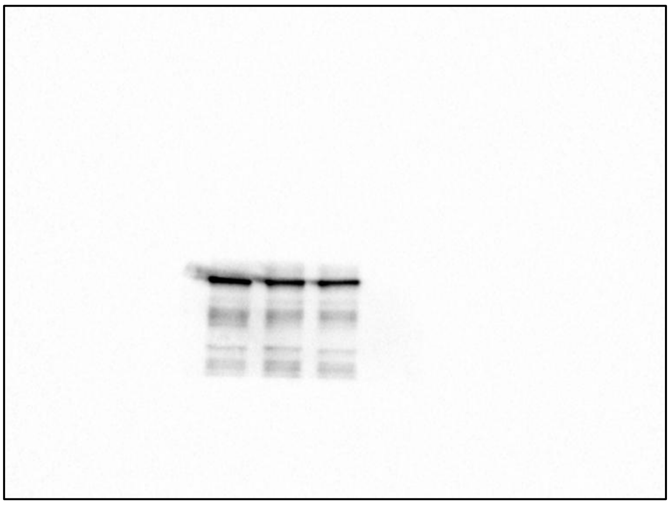

Fig 5F

IP:Flag IB:Kbhb

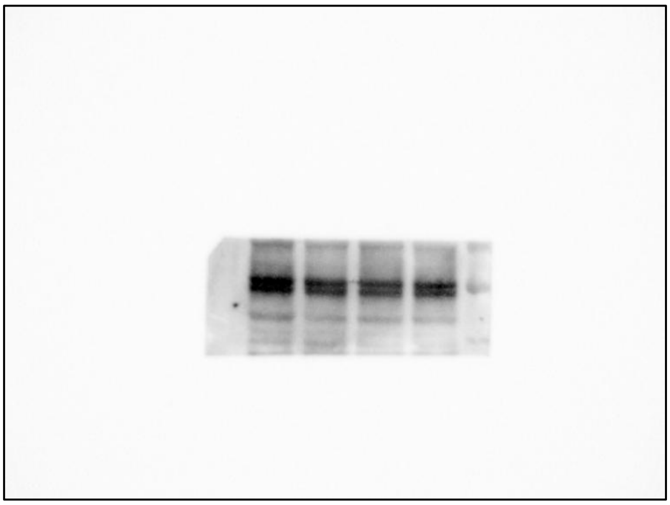

IP:Flag IB:Flag

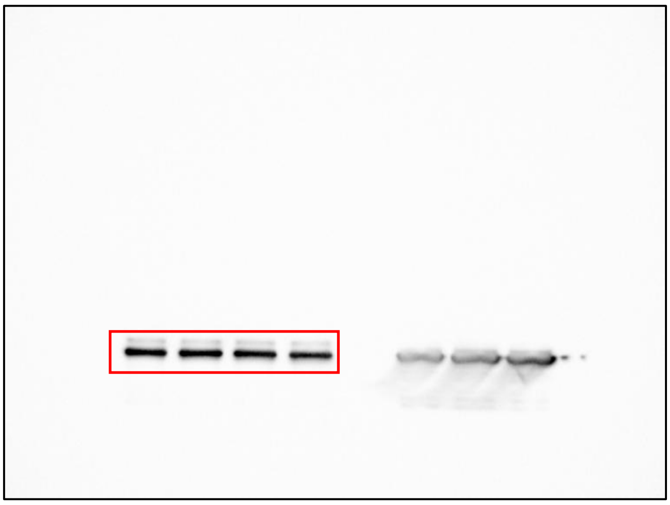

Input:Flag

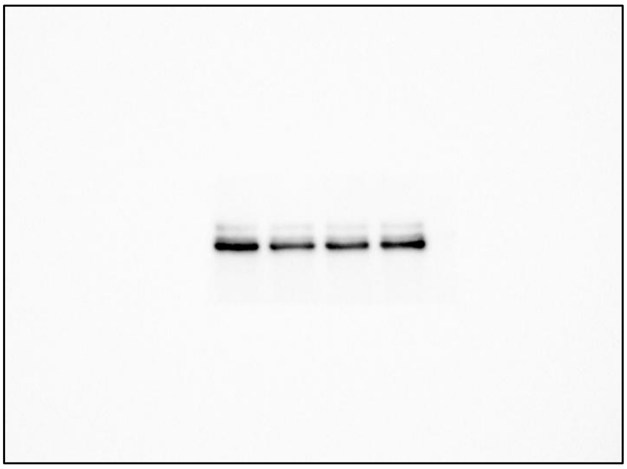

Fig 6A

IP:OXCT1 IB:Kbhb

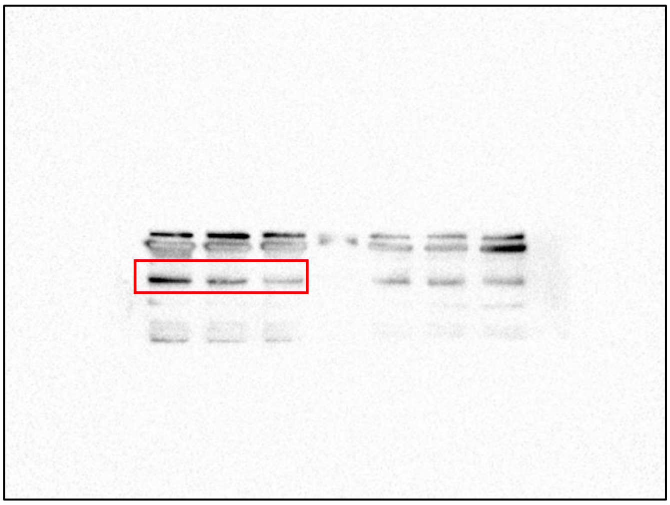

IP:OXCT1 IB:OXCT1

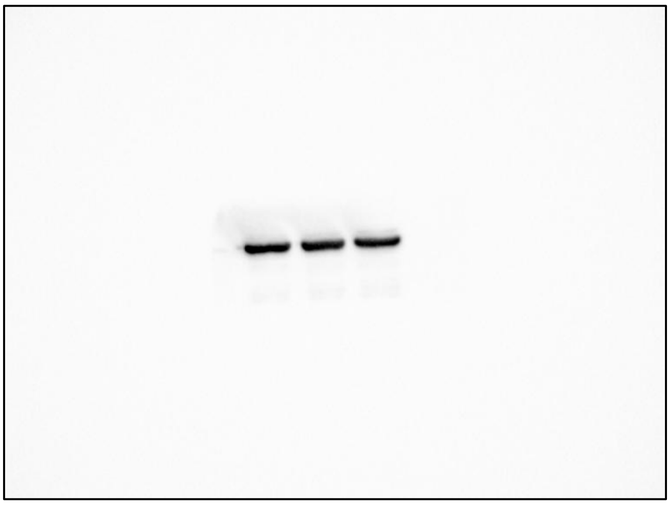

Input:OXCT1

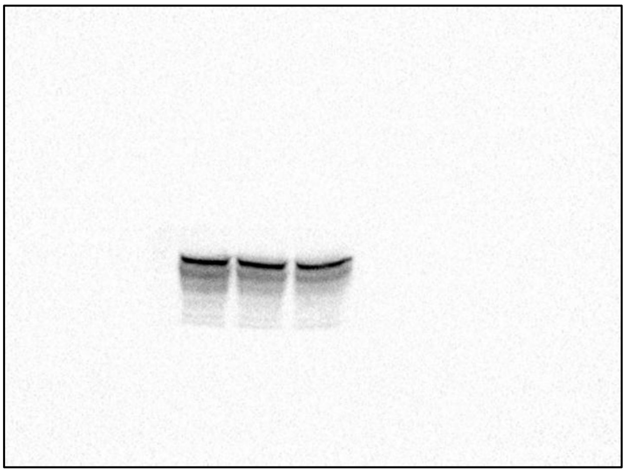

Fig 6B

IP:OXCT1 IB:Kbhb

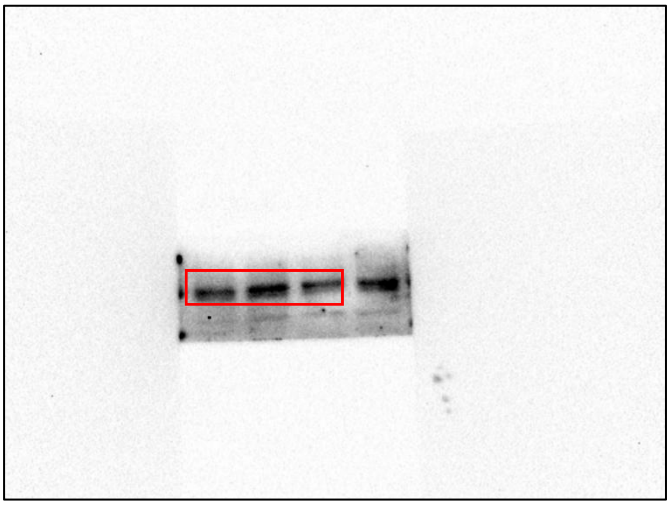

IP:OXCT1 IB:OXCT1

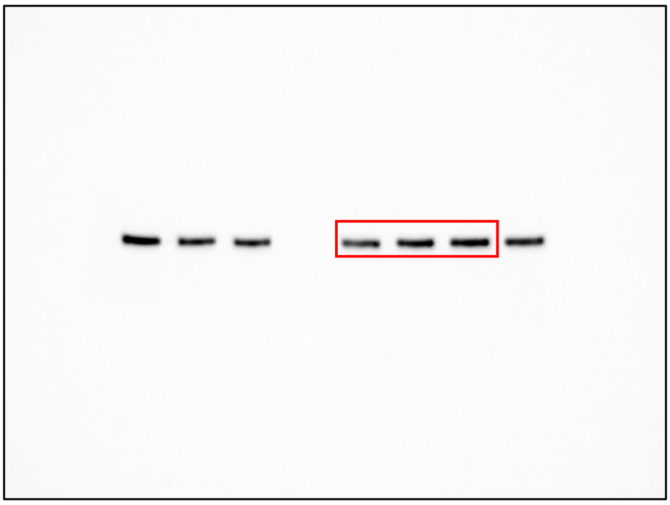

Input:OXCT1

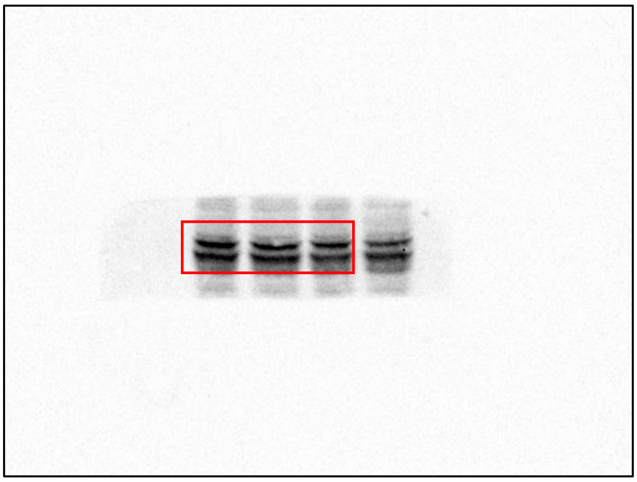

Fig 6C

IP:OXCT1 IB:Kbhb

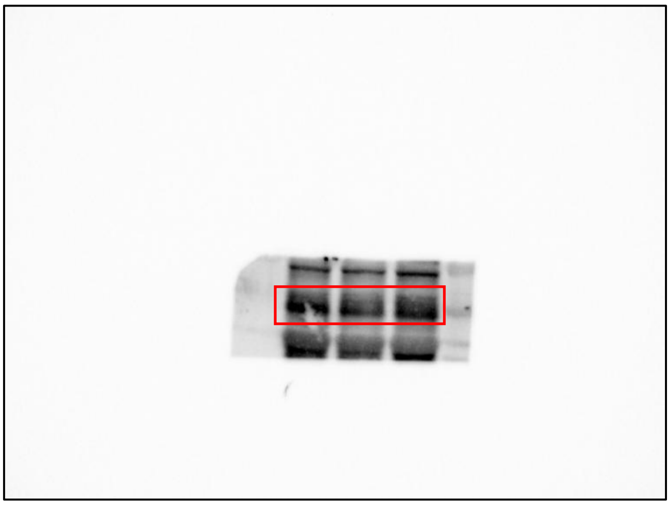

IP:OXCT1 IB:OXCT1

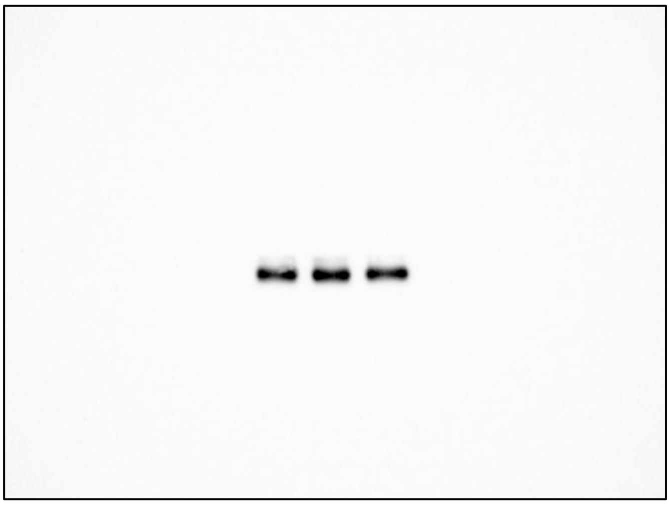

Input:OXCT1

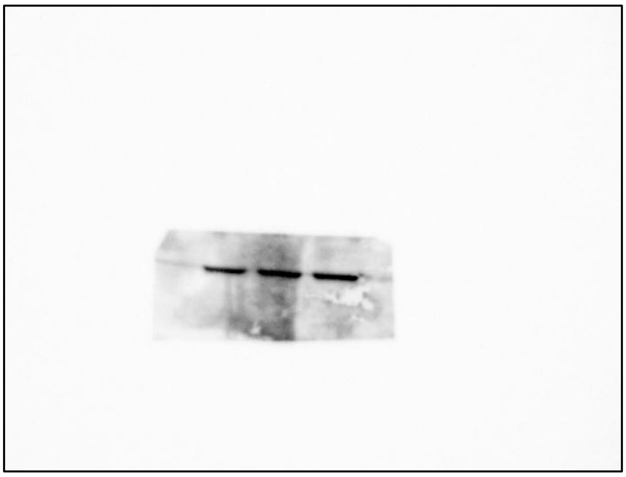

Fig 6D

IP:Flag IB:His

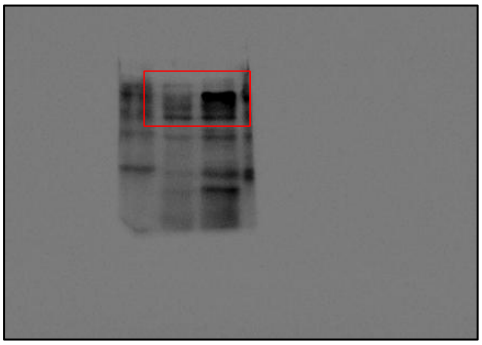

IP:Flag IB:Flag

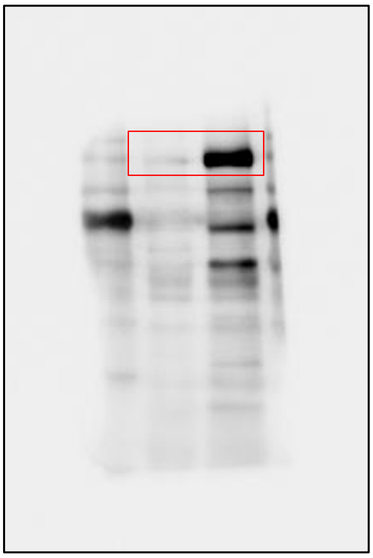

Input:His

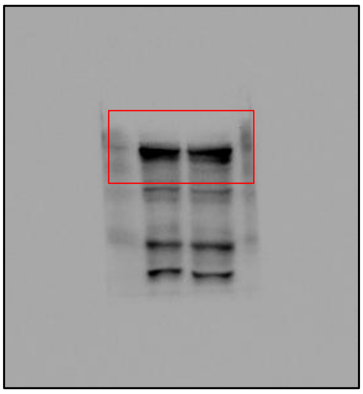

Input:Flag

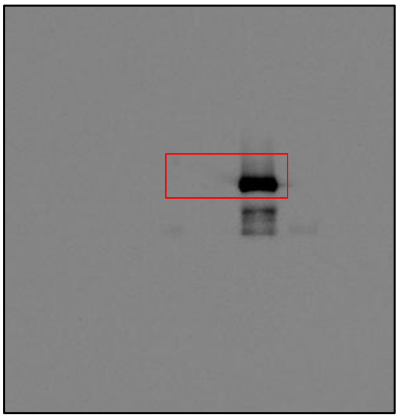

IP:His IB:Flag

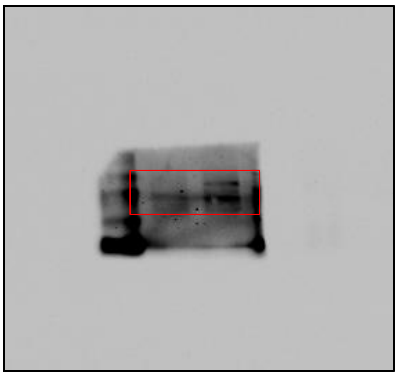

IP:His IB:OXCT1

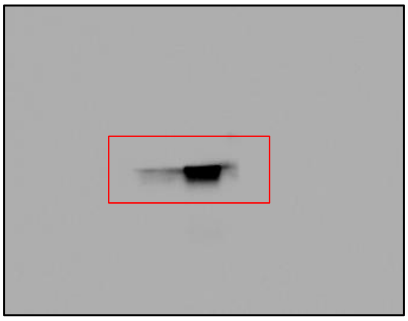

Input:Flag

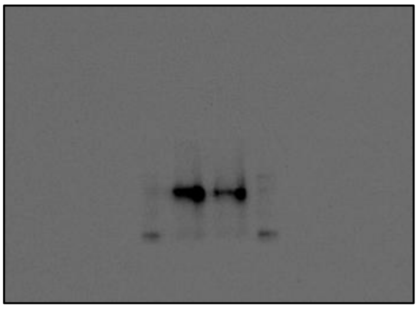

Input:OXCT1

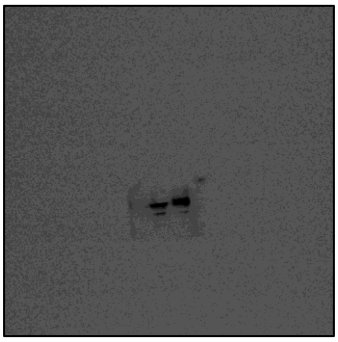

Fig 6E

IP:Flag IB:Kbhb

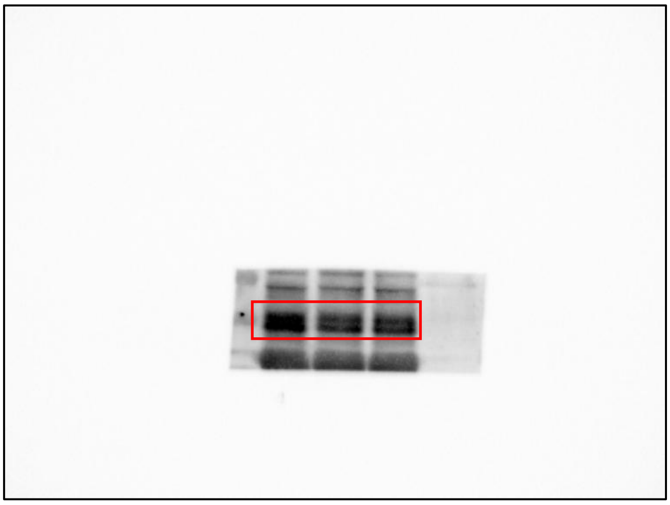

IP:Flag IB:Flag

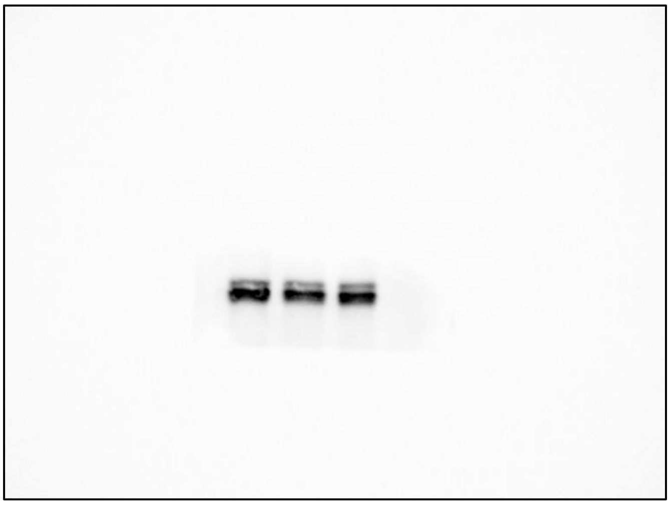

Input:Flag

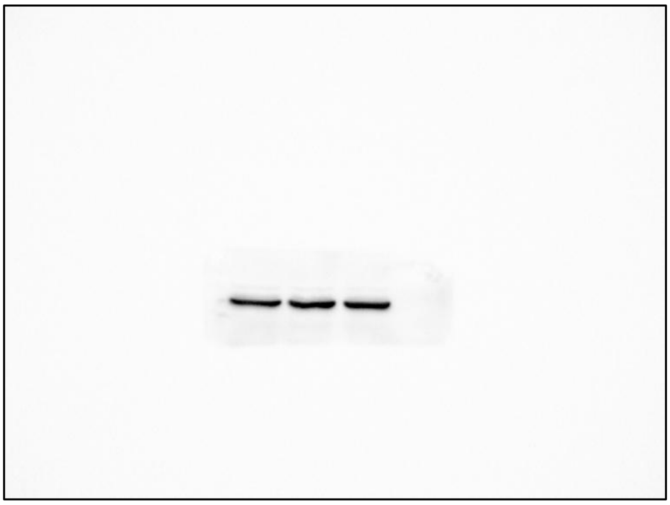

Fig 6F

IP:HMGCS2 IB:Kbhb

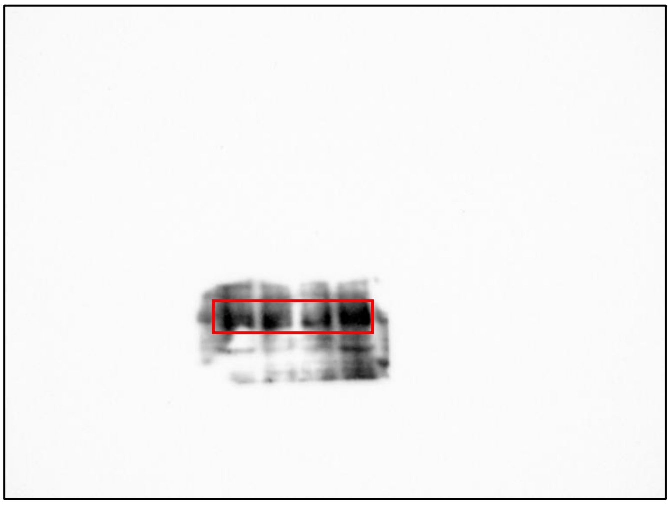

IP:HMGCS2 IB:HMGCS2

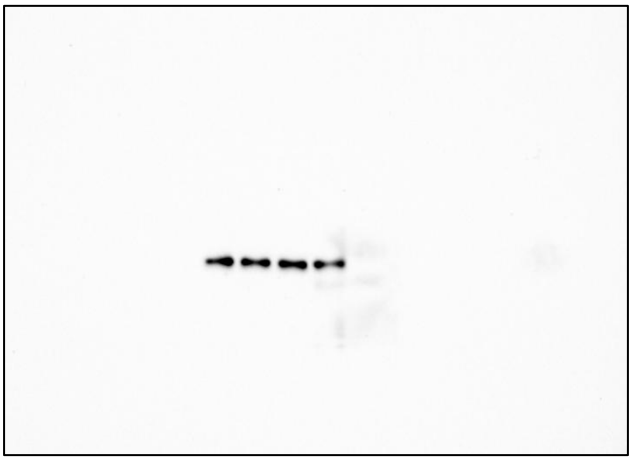

Input:HMGCS2

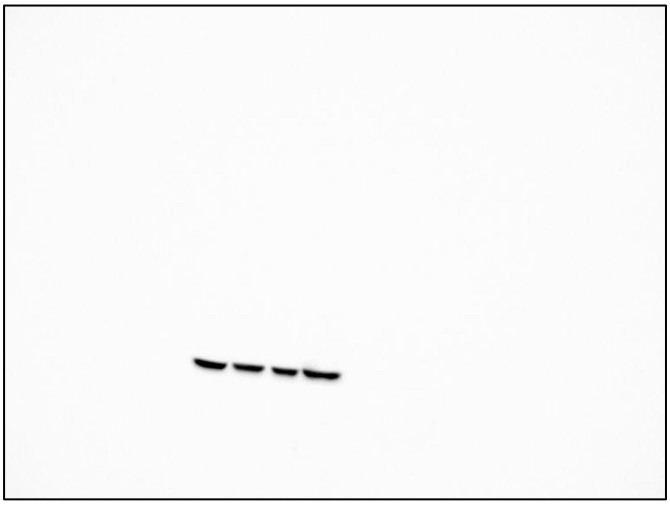

Fig7E

Kbhb (WCL)

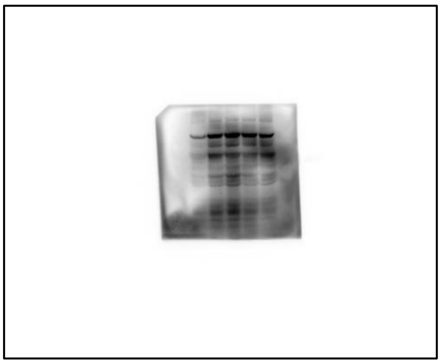

OXCT1(WCL)

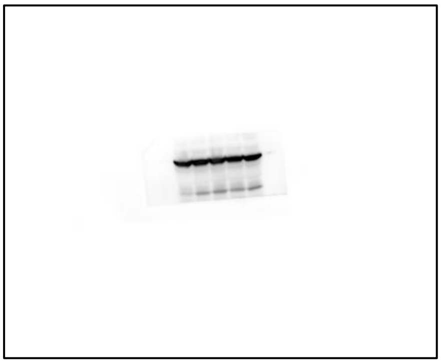

β-actin (WCL)

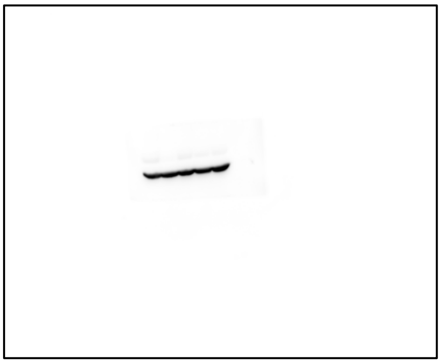

Kbhb (Co-IP)

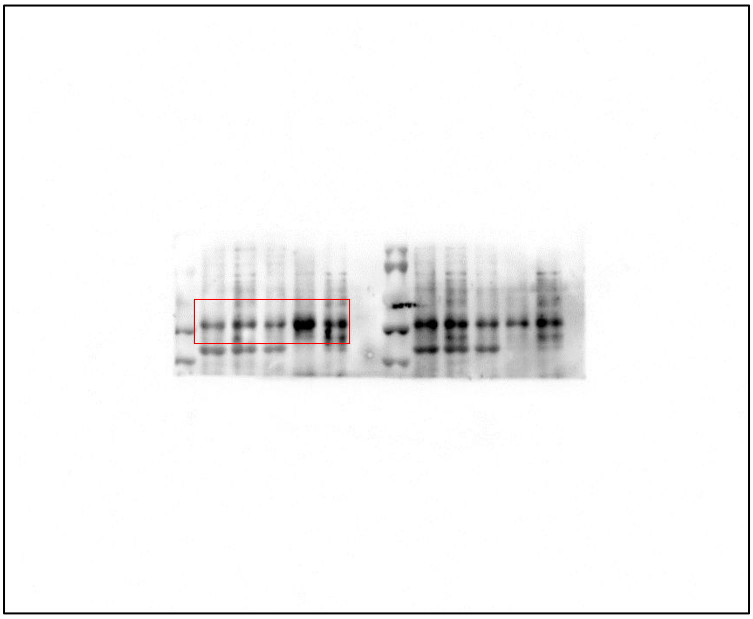

OXCT1(Co-IP)

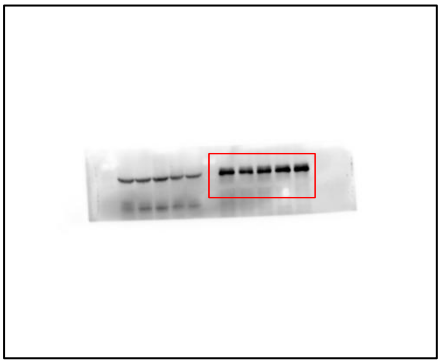

Fig S1A

IB:Kbhb Skeletal Muscle

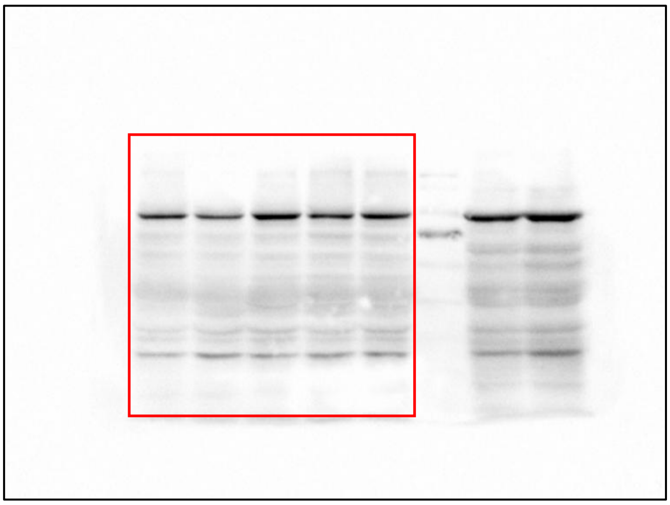

IB:OXCT1 Skeletal Muscle

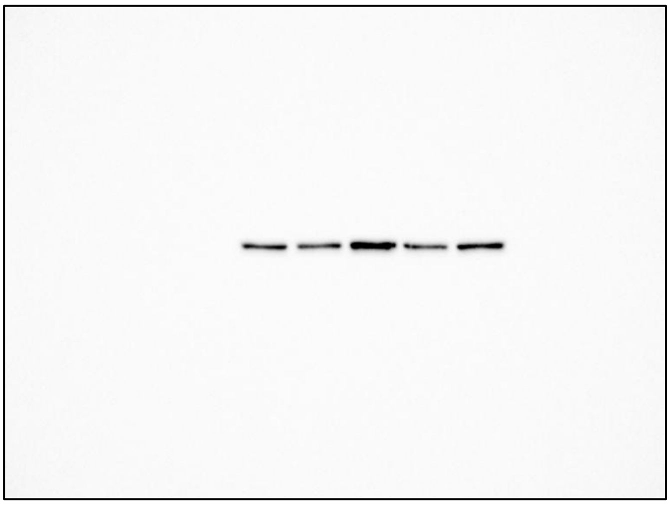

IB:α-tubulin Skeletal Muscle

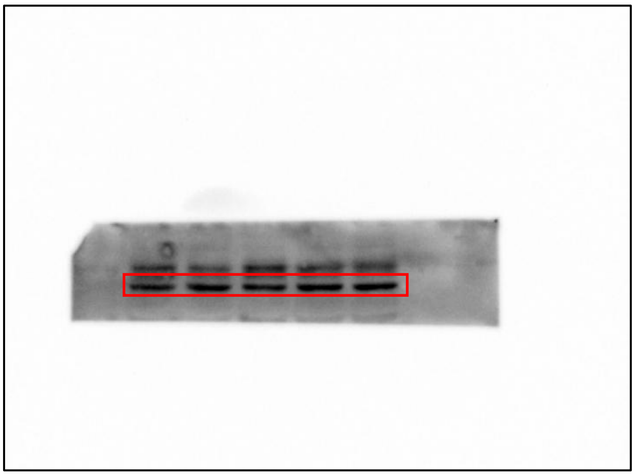

IB:Kbhb Brain

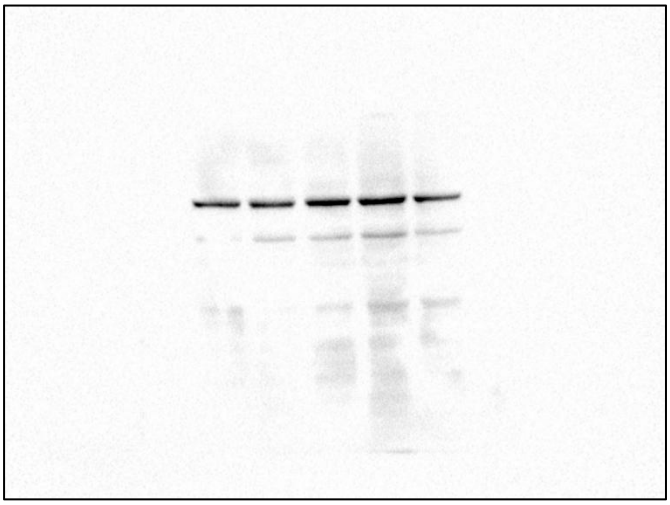

IB:OXCT1 Brain

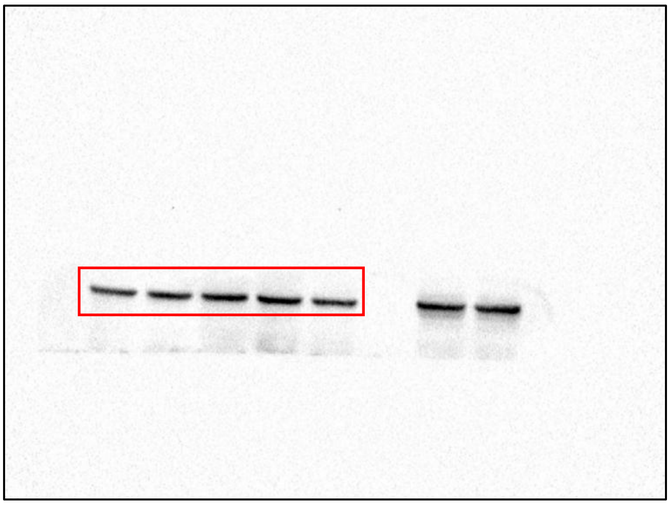

IB:α-tubulin Brain

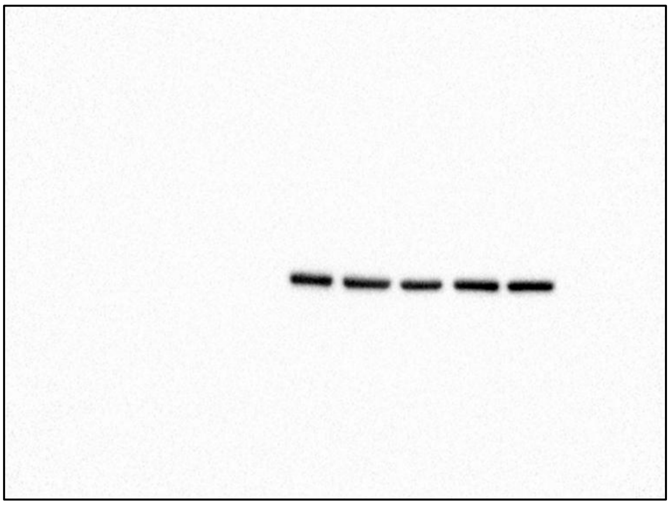

Fig S1B

IP:OXCT1 IB:Kbhb Skeletal Muscle

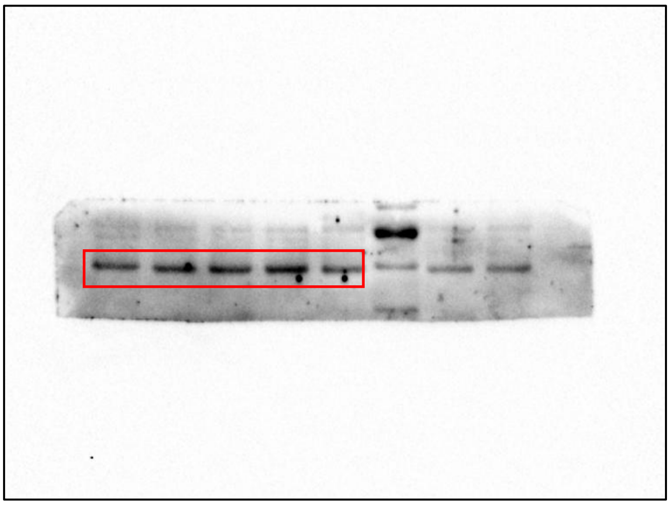

IP:OXCT1 IB:OXCT1 Skeletal Muscle

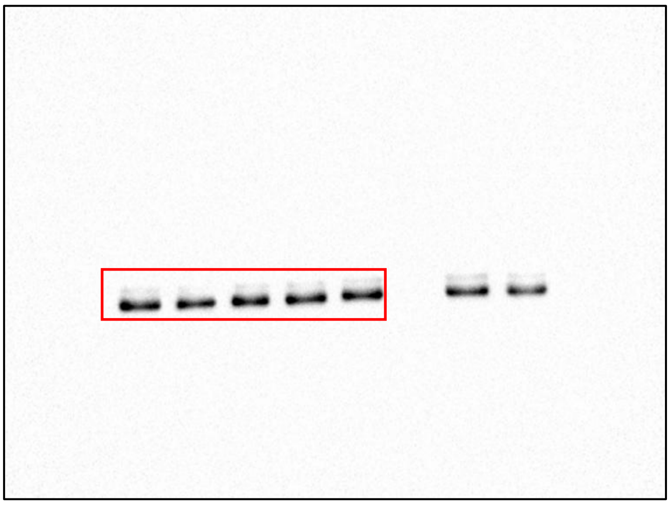

Input:OXCT1 Skeletal Muscle

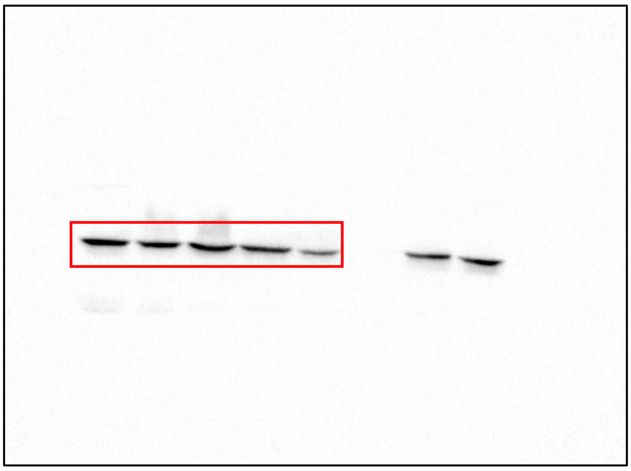

IP:OXCT1 IB:Kbhb Brain

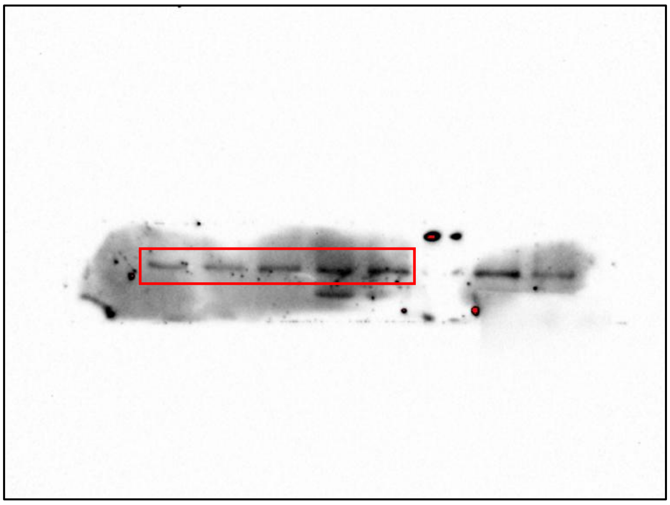

IP:OXCT1 IB:OXCT1 Brain

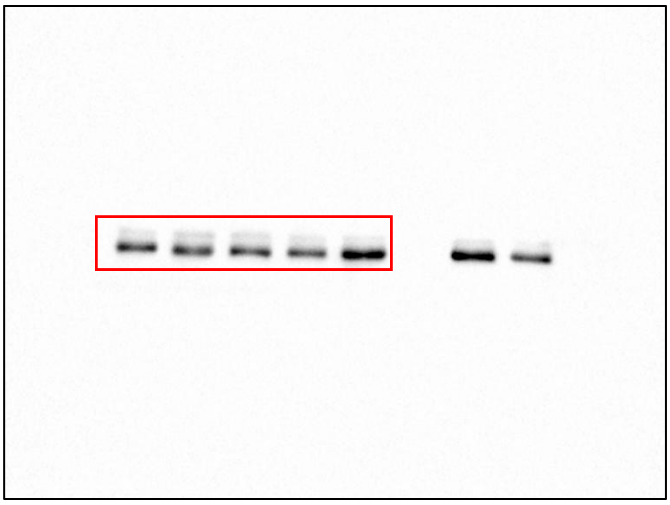

Input:OXCT1 Brain

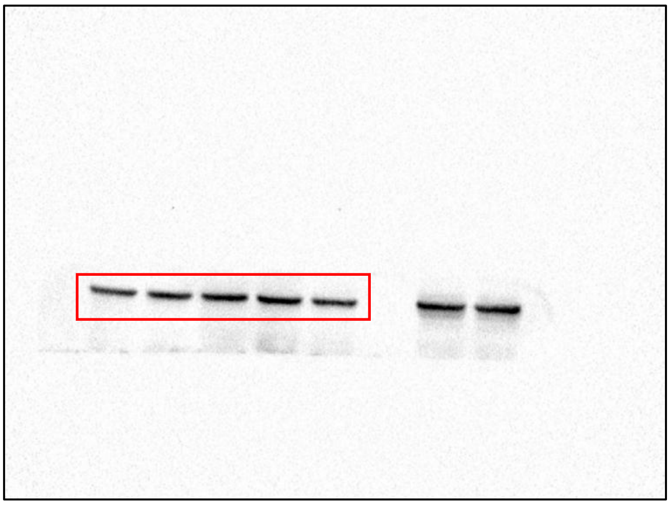

Supplement: Original images for Western blotting data [file mmc1.pdf]
