# Supplementary material for: β-hydroxybutyrate serves as a regulator in ketone body metabolism through lysine β-hydroxybutyrylation
Source: J Biol Chem. 2025 Apr 2;301(5):108475. doi: 10.1016/j.jbc.2025.108475 (PMC12147175; doi:10.1016/j.jbc.2025.108475)
Supplement: Supplementary Figures [file mmc2.docx]

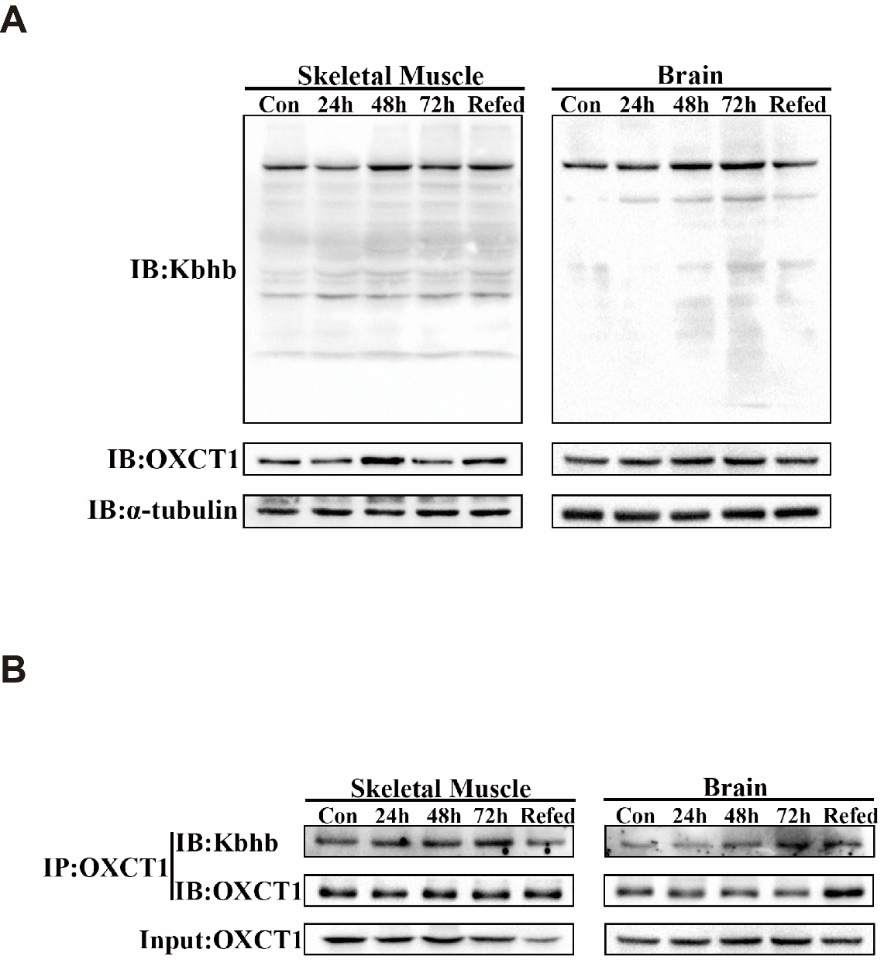


**Figure S1.** The levels of Kbhb modification in skeletal muscle and brain tissues were evaluated under different starvation conditions. (A) Global Kbhb modification levels and OXCT1 expression in mice's organs (Skeletal muscle and Brain) after starvation and refed. (B) OXCT1-specific Kbhb modification levels in organs (Skeletal muscle and Brain) after starvation and refed by co-immunoprecipitation assay. The whole cell lysate (WCL) samples of the brain used are identical to those in Figure S1A, and the co-immunoprecipitation assay was performed using these samples simultaneously. Therefore, the bands of OXCT1 for brain samples in Figure S1A were used in this panel. The bands for the Skeletal muscle sample were not reused because the global and OXCT1-specific Kbhb modifications were conducted at separate times.


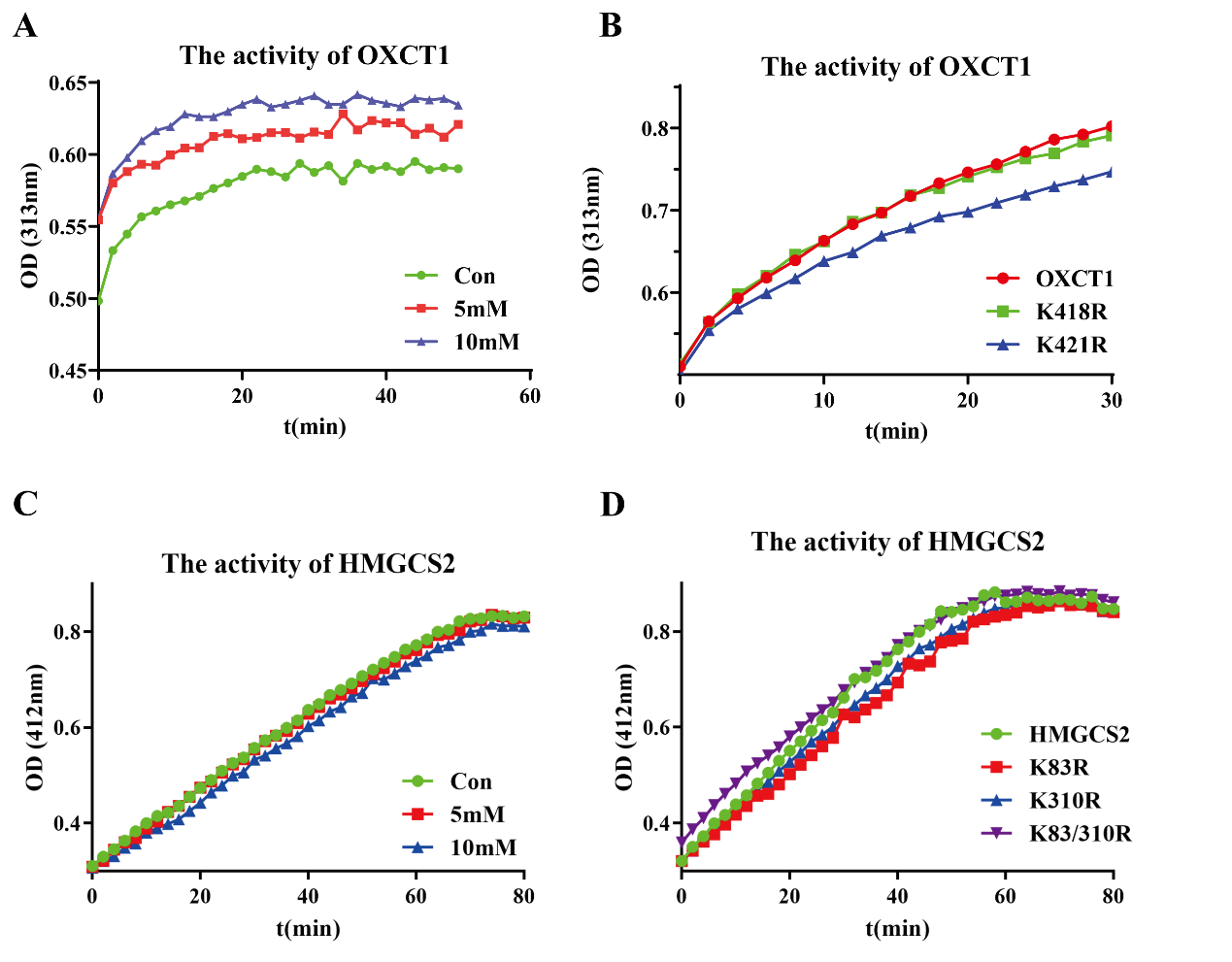


**Figure S2.** (A) The original data was collected every two minutes and lasted for 50 minutes to show the whole enzyme activity curve of OXCT1 in cells treated with different concentrations of β-HB (0, 5, and 10 mM). (B) The original data was collected every two minutes and lasted for 50 minutes to show the whole enzyme activity curve of OXCT1 in cells transfected with OXCT1 (wild type, K418R, and K421R) plasmids, respectively. (C) The original data was collected every two minutes and lasted for 80 minutes to show the whole enzyme activity curve of HMGCS2 in cells treated with different concentrations of β-HB (0, 5, and 10 mM). (D) The original data was collected every two minutes and lasted for 80 minutes to show the whole enzyme activity curve of HMGCS2 in cells transfected with HMGCS2 (wild type, K83R, K310R, and double-sites mutant K83/310R) plasmids, respectively.


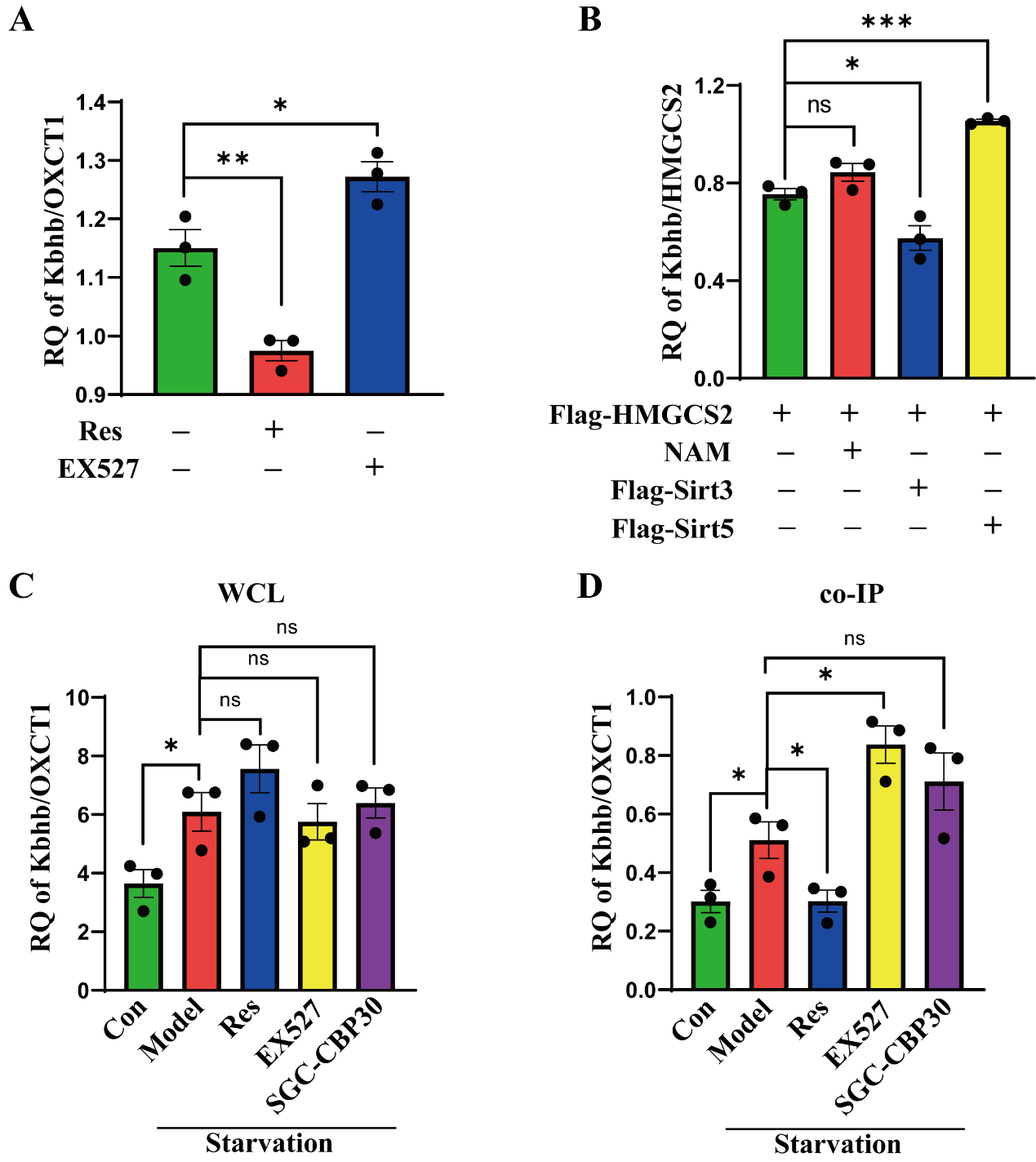


**Figure S3**. (A) The quantification of band intensity of Figure 6C. (B) The quantification of band intensity of Figure 6F. (C and D) The quantification of band intensity of WCL (Whole Cell Lysate) and Co-IP (co-immunoprecipitation) samples in Figure 7E.
