# Supplementary material for: β-hydroxybutyrate serves as a regulator in ketone body metabolism through lysine β-hydroxybutyrylation
Source: J Biol Chem. 2025 Apr 2;301(5):108475. doi: 10.1016/j.jbc.2025.108475 (PMC12147175; doi:10.1016/j.jbc.2025.108475)
Supplement: Supplementary Table S1 [file mmc3.docx]

**Table S1.** The details of siRNA sequences of human OXCT1 and HMGCS2.

| **Genes** | **Name** | **Sequence** |
| --- | --- | --- |
| hOXCT1 | siRNA-1 | GTACTTATCTGGTGAATTA |
|  | siRNA-2 | GGACACGTCGATCTGACAA |
|  | siRNA-3 | GGGAGGTGCTATGGATTTA |
| hHMGCS2 | siRNA-1 | CTTCACCCTTGACGATTTA |
|  | siRNA-2 | GGAGTACCCAATAGTGGAT |
|  | siRNA-3 | GCAGCATCGCCGAAAGTAT |
